# Supplementary material for: Genome-wide alterations of uracil distribution patterns in human DNA upon chemotherapeutic treatments
Source: eLife. 2020 Sep 21;9:e60498. doi: 10.7554/eLife.60498 (PMC7505663; doi:10.7554/eLife.60498)
Supplement: Supplementary file 1. — List of the investigated samples (table 1); list of applied tools (table 2); pre-processing including blacklisting and additional statistics (table 3); and methods to determine uracil enrichment pattern. All applied processing steps are given in generalized command lines. [file elife-60498-supp1.pdf]

Supplementary file 1

Detailed analysis pipeline – methods of U-DNA-Seq data analysis

Supplementary file 1-table 1. Description of the investigated samples.

abbreviation	description
WT	wild type HCT116 that is MMR deficient
NT_UGI	non-treated UGI-expressing HCT116
NT_UGI_MMR	non-treated UGI-expressing HCT116, MMR proficient variant
5FdUR_UGI	5FdUR treated UGI-expressing HCT116
5FdUR_UGI_MMR	5FdUR treated UGI-expressing HCT116, MMR proficient variant
RTX_UGI	RTX treated UGI-expressing HCT116
RTX_UGI_MMR	RTX treated UGI-expressing HCT116, MMR proficient variant
NT_UGI_ctr	empty bead control for U-DNA-IP in non-treated UGI-expressing HCT116
5FdUR_UGI_ctr	empty bead control for U-DNA-IP in 5FdUR treated UGI-expressing HCT116
NT_UGI_H3K36me3	ChIP-seq for H3K36me3 in non-treated UGI-expressing HCT116
RTX_UGI_H3K36me3	ChIP-seq for H3K36me3 in RTX treated UGI-expressing HCT116

Supplementary file 1-table 2. Details on the applied tools.

Program package	tool	purpose	Version	Link
FastQC		Quality checking	0.11.7	https://www.bioinformatics.babraham.ac.uk/projects/fastqc
Trimmomatic		Adapter and quality trimming	0.36	https://github.com/timflutre/trimmomatic (Bolger, Lohse, & Usadel, 2014)
BWA	MEM	*Burrows-Wheeler Aligner*	0.7.17	https://github.com/lh3/bwa (H. W. Li, 2013)
samtools	view	Filtering reads in bam files	1.9	https://github.com/samtools/samtools (H. Li et al., 2009)
	merge	Concatenating bam files		
	sort	Sorting reads in a bam file (required by most of the downstream application)		
	index	Indexing bam files (required by most of the downstream application)		
	idxstats	Reporting the numbers of mapped and unmapped reads in an indexed bam file along the chromosomes and scaffolds in the reference genome		
Picard Tools	MarkDuplicates	Filtering out reads corresponding to PCR or optical duplicates	1.95	http://broadinstitute.github.io/picard
deepTools	multiBamSummary	Genome-wide comparison of multiple bam files regarding the read coverage in defined sized bins	3.2.1	https://github.com/deeptools/deepTools/releases (Ramírez et al., 2016)
	bamCoverage	Calculating genome scaled read coverage tracks in databins and with the option of smoothing resulting in bedgaph or bigWig files		

	bigwigCompare	Comparing two bigWig files in many different ways e.g. log2 ratio or subtract		
	multiBigWigSummary	Genome-wide comparison of multiple bw files in defined sized bins		
	plotCorrelation	Calculating and plotting the correlation coefficients from the results of multiBigWigSummary or multiBamSummary		
bedtools2	merge	Merging intervals in files in many different ways	2.28.0	https://github.com/arq5x/bedtools2 (Quinlan & Hall, 2010)
	subtract	Subtracting intervals in files in different ways		
	complement	Taking the complement of an interval file comparing to a reference genome		
	intersect	Extracting overlapping fractions of interval files in many different ways		
	jaccard	Calculating Jaccard indices (ratio of base numbers in the intersect over the union of two interval files)		
	annotate	Comparing query interval file to a set of database interval files, and reporting overlap ratio and/or the number of overlapping intervals for each interval in the query bed file		
GIGGLE	sort_bed	A script utilizing also bgzip, to sort and compress bed files for giggle search	1.0	https://github.com/ryanlayer/giggle (Layer et al., 2018)
	Index	Special indexing applied for the library of the database interval files		
	search	Scoring colocalization between a query and indexed database interval files		
kentUtils	bigWigToWig	conversion tool from the binary coded bigWig to a text format Wiggle file		https://github.com/ucscGenomeBrowser/kent (Kuhn, Haussler, & Kent, 2013)
	bigWigAverageOverBed	Averaging scores in a bw files for the intervals given in a bed files		
	liftOver	Converting genomic coordinates in a bed file from one to another reference genome version		
MACS2	callpeak	Calling peaks of read coverage, standard tool in ChIP-seq data analysis	2.1.2	https://github.com/taoliu/MACS (Feng, Liu, & Zhang, 2011; Zhang et al., 2008)
Segway package	Genomedata load	Preparation of genomedata file for the Segway train and annotate	1.4.4	http://noble.gs.washington.edu/proj/genomedata/ (Hoffman, Buske, & Noble, 2010)
	Segway	Learning algorithm to define genomic segments with characteristic patterns, performing genome segmentation.	3.0	https://segway.hoffmanlab.org/ (Chan et al., 2017; Hoffman et al., 2012)
	Segtools	Calculating signal distribution and other features on the identified genomic segments, and preparing heatmaps and plots	1.2.4	http://noble.gs.washington.edu/proj/segtools (Buske, Hoffman, Ponts, Le Roch, & Noble, 2011)
Python	Seaborn, Matplotlib, Pandas	Seaborn is a library for making statistical graphics in Python. It is built on top of matplotlib and closely integrated with pandas data structures.	0.10.1	https://pypi.org/project/seaborn/ (Hunter, 2007; McKinney & others, 2010)
R		Environment for statistical computing	3.5.1	https://www.R-project.org/ (R Core Team, 2018)
Linux command-line utilities	awk	Text pattern scanning and processing tool to handle big data in text format in many different ways	4.0.2	Copyright © 2016 Free Software Foundation, Inc.
	sort	Sorting information of a text file in many different ways	8.22	
	grep	Handling and processing big data in text format in many different ways	2.20	

Preprocessing

Raw sequencing data for both input and enriched samples were first quality checked (using FastQC) and trimmed (using Trimmomatic (Bolger et al., 2014)), then aligned to the human reference genome (using BWA (H. W. Li, 2013)). The GRCh38.d1.vd1 reference genome sequence (basically the GCA_000001405.15_GRCh38_no_alt_analysis_set (Jensen, Ferretti, Grossman, & Staudt, 2017)) was selected that contains additional decoy segments (GenBank Accession GCA_000786075) and virus sequences to help eliminating potential contaminating reads from the core alignment (https://gdc.cancer.gov/about-data/data-harmonization-and-generation/gdc-reference-files (Gao et al., 2019)). Aligned reads were sorted (using samtools sort (H. Li et al., 2009)), and duplicates were marked (using Picard Tools) resulting in bam files (raw aligned reads). Reads with MAPQ=0 were removed from raw bam files using samtools view as follows.

```
$ samtools view -b -h -q 1 NAME.sorted.dedup.bam -L list_of_chr_bam.bed -o
NAME.MAPQfiltered.bam
```

# list_of_chr_bam.bed is a 3 column tab delimited text file with indication of the name of chromosomes, their starts and their ends within the applied reference genome assembly.

Hereafter, all applied command lines are provided in a generalized way, where „NAME" consists of the following indications: treatments_cellType_replicationNo_sampleType. In this study, „treatments" can be WT, NT_UGI, RTX_UGI, or 5FdUR_UGI; cellTypes can be HCT116, HCT116MMR (MMR proficient variant of HCT116) or K562; replicationNo can be rep1, rep2, or merged; sampleType can be IP (=enriched), son (=input), or combination of these in case of log2 ratio or other files derived from two samples (e.g. IP_vs_son). Where distinction is necessary, a note is inserted in brackets after the „NAME" (e.g. NAME(rep1)…), otherwise the command was applied on all of the samples. The names of the files deposited into the Gene Expression Omnibus (GEO, accession number GSE126822) also follow this scheme.

Cell type specific blacklists were created by combination of the universal DAC blacklist (https://www.encodeproject.org/files/ENCFF419RSJ) suggested for general use by ENCODE consortium (Amemiya, Kundaje, & Boyle, 2019) and a cell type specific blacklist defined based on Ultra High Signal (UHS) regions and low-mappability regions detected in the input sequencing data (Figure 2-figure supplement 2). This procedure involves deepTools (Ramírez et al., 2016), some tools from the kentUtils package of the UCSC (Kuhn et al., 2013), R and linux command-line utilities. The steps are as follows:

<u>Method to define Ultra High Signal (UHS) regions:</u>

(1) Compute coverage tracks without smoothing for input samples only.

```
$ bamCoverage -b NAME.sorted.dedup.bam -o NAME.bin100bp.no_smooth.RPGC.bw --binSize
100 --verbose --normalizeUsing RPGC --effectiveGenomeSize 2913022398 –p 16
```

(2) Compute histogram on coverage signals to define a threshold above which UHS regions are considered (Figure 2-figure supplement 2C).

```
$ bigWigToWig NAME.bin100bp.no_smooth.RPGC.bw NAME.bin100bp.no_smooth.RPGC.wig
```

In R:

```
> NAME <- read.delim("NAME.bin100bp.no_smooth.RPGC.wig", header=FALSE)
> hist(NAME$V4, breaks = 100)
> hist(NAME$V4, breaks = 3000, xlim = c(-0.2, 400), ylim = c(0, 5000))
```

A threshold at coverage signal = 50 was decided.

(3) Compute interval (bed) files describing UHS regions.

# deleting lines that are only for indication the bedGraph sections and then selecting data bins that are above the threshold 50

```
$ grep -vwF "bedGraph" NAME.bin100bp.no_smooth.RPGC.wig | awk ' $4 > 50 ' >
NAME.bin100bp.no_smooth.RPGC.UHS.bed
```

# merging neighboring data bins to a single interval, then sorting, then printing column 1, 2, and 3, and also the line number in each line of the bed file

```
$ bedtools merge -i NAME.bin100bp.no_smooth.RPGC.UHS.bed | sort -k1,1 -k2,2n | awk
'{print $1 "\t" $2 "\t" $3 "\t" NR}' > NAME.bin100bp.no_smooth.RPGC.UHS.numbered.bed
```

# calculating average log2 uracil enrichment value for the intervals in the bed file, it is added to the column 5

```
$ bigWigAverageOverBed -bedOut=NAME.bin100bp.no_smooth.RPGC.UHS.scored.bed
NAME.bin100bp.no_smooth.RPGC.bw NAME.bin100bp.no_smooth.RPGC.UHS.numbered.bed DEL.tab
```

# sorting, then printing again with the right format of the float numbers in the column 5

```
$ sort -k1,1 -k2,2n NAME.bin100bp.no_smooth.RPGC.UHS.scored.bed | awk '{printf "%s\t",
$1; printf "%s\t", $2; printf "%s\t", $3; printf "%s\t", $4; printf "%f\n", $5}' >
NAME.bin100bp.no_smooth.RPGC.UHS.scored2.bed
```

<u>Method to define low-mappability regions:</u>

(1) Compute coverage tracks without smoothing and also without normalizing for the input bam files, original and filtered ones (in filtered one, the MAPQ=0 reads were removed using samtools view, see above).

```
$ bamCoverage -b NAME.sorted.dedup.bam -o NAME.bin100bp.no_smooth.no_norm.bw --binSize
100 --verbose  --effectiveGenomeSize 2913022398 –p 16
```

```
$ bamCoverage -b NAME.MAPQfiltered.bam -o
NAME.MAPQfiltered.bin100bp.no_smooth.no_norm.bw --binSize 100 --verbose  --
effectiveGenomeSize 2913022398 –p 16
```

(2) Compute log2 ratio track of coverage original / filtered (using deepTools/BamCompare for bins 100bp).

```
$ bigwigCompare -b1 NAME.bin100bp.no_smooth.no_norm.bw -b2
NAME.MAPQfiltered.bin100bp.no_smooth.no_norm.bw -o NAME.original_vs_filtered.log2.bw -
of bigwig --binSize 100 --skipZeroOverZero --pseudocount 2 1 -v -p 16
```

(3) Compute histogram on log2 ratio signals (Figure 2-figure supplement 2D).

```
$ bigWigToWig NAME.original_vs_filtered.log2.bw NAME.original_vs_filtered.log2.wig
```

In R (R Core Team, 2018)*:*

```
> NAME_of <- read.delim("NAME.original_vs_filtered.log2.wig", header=FALSE)
> hist(NAME_of$V4, breaks = 100)
> hist(NAME_of$V4, breaks = 100, xlim = c(-0.2, 4), ylim = c(0, 1500000))
```

A threshold at log2 ratio signal = 1.0 was decided, that means that half of the reads in the given bin have MAPQ=0.

(4) Compute interval (bed) files that describe regions with more than 50% ambiguously mapped reads considered as low-mappability regions.

```
$  grep -vwF "bedGraph" NAME.original_vs_filtered.log2.wig | awk ' $4 > 1 ' >
NAME.original_vs_filtered.log2.blackMAPQ.bed

$ bedtools merge -i NAME.original_vs_filtered.log2.blackMAPQ.bed | sort -k1,1 -k2,2n |
awk '{print $1 "\t" $2 "\t" $3 "\t" NR}' >
NAME.original_vs_filtered.log2.blackMAPQ.numbered.bed

$ bigWigAverageOverBed -bedOut=NAME.original_vs_filtered.log2.blackMAPQ.scored.bed
NAME.original_vs_filtered.log2.bw
NAME.original_vs_filtered.log2.blackMAPQ.numbered.bed DEL.tab

$ sort -k1,1 -k2,2n NAME.original_vs_filtered.log2.blackMAPQ.scored.bed | awk '{printf
"%s\t", $1; printf "%s\t", $2; printf "%s\t", $3; printf "%s\t", $4; printf "%f\n",
$5}' > NAME.original_vs_filtered.log2.blackMAPQ.scored2.bed
```

Cell type specific blacklists were then created by merging the DAC blacklist (ENCFF419RSJ), the UHS and the low-mappability regions using bedtools merge with the parameter - d500 to avoid 500 bases or shorter gaps with obviously no biological meaning (cf. purple and black intervals on IGV view at Figure 2-figure supplement 2B). For HCT116 cell line specific blacklist, all the corresponding input samples were used and the derived intervals were merged together.

```
$ cat NAME1.bin100bp.no_smooth.RPGC.UHS.scored2.bed
NAME2.bin100bp.no_smooth.RPGC.UHS.scored2.bed {…}
```

```
NAMEn.bin100bp.no_smooth.RPGC.UHS.scored2.bed | sort -k1,1 -k2,2n >
united_sorted_UHS_HCT116.bed
```

```
$ bedtools merge -i united_sorted_UHS_HCT116.bed > UHS_HCT116.bed
```

```
$ cat NAME1.original_vs_filtered.log2.blackMAPQ.scored2.bed
NAME2.original_vs_filtered.log2.blackMAPQ.scored2.bed {…}
NAMEn.original_vs_filtered.log2.blackMAPQ.scored2.bed | sort -k1,1 -k2,2n >
united_sorted_blackMAPQ_HCT116.bed
```

```
$ bedtools merge -i united_sorted_blackMAPQ_HCT116.bed > blackMAPQ_HCT116.bed
```

```
$ cat ENCFF419RSJ.bed UHS_HCT116.bed blackMAPQ_HCT116.bed | sort -k1,1 -k2,2n >
united_sorted_blacklist_HCT116.bed
```

```
$ bedtools merge -i united_sorted_blacklist_HCT116.bed -d 500 > blacklist_HCT116.bed
```

The effective genome size was calculated by subtracting the blacklisted and the originally masked regions of the reference genome.

```
$ bedtools subtract -a list_of_chr_bam.bed -b blacklist_HCT116.bed >
not_blacklisted_HCT116.bed
```

```
$ bedtools nuc -fi GRCh38.d1.vd1.fa -bed not_blacklisted_HCT116.bed >
not_blacklisted_HCT116_nuc.bed
```

```
$ awk '{(sum1+=$6) (sum2+=$9) (sum3+=$7) (sum4+=$8) (sum5+=$10) (sum6+=$11)
(sum7+=$12)} END {print sum1 "\t" sum2"\t" sum3 "\t" sum4 "\t" sum5 "\t" sum6 "\t"
sum7}' not_blacklisted_HCT116_nuc.bed
```

```
825630405    826937345    570444697    570830493    165010872    99    2958853872
```

# Note that awk will sum up the number from the head line too – so column number has to be subtracted.

number of A	number of T	number of C	number of G	number of N	Nº other	length
825630399	826937336	570444690	570830485	165010862	88	2958853860

Thereby, the effective genome size was calculated for the analysis of the HCT116 samples as 2793842910 (length – number of N – Nº other). For the MMR proficient HCT116 cells, a separate blacklist was calculated. Accordingly, the effective genome size has been changed to 2804512581.

GC content for the effective part of the reference genome was found to be 40.85% for both MMR deficient and proficient HCT116 cells. This was calculated according to the formula: (number of C + number of G) / effective genome size.

This cell type specific united blacklist was applied in samtools view to BAM files that were also filtered for MAPQ=0 reads previously.

```
$ samtools view -b -h NAME.MAPQfiltered.bam -L blacklist_HCT116.bed -o
NAME.blacklist.bam -U NAME.filtered_blacklisted.bam

$ samtools index NAME.filtered_blacklisted.bam

$ samtools idxstats NAME.filtered_blacklisted.bam >
NAME.filtered_blacklisted.bam.idxstats.csv
```

Supplementary file 1-table 3. Number of reads in samples during the pre-processing steps. All samples and replicates are shown here that were sequenced in the frame of the present publication. Number of raw reads means read number before starting alignment (the sum of the mapped and unmapped read numbers). Uniquely mapped read means that MAPQ is not zero. The samples are as follows: non-treated wild-type (WT), non-treated UGI-expressing (NT_UGI), 5FdUR treated UGI-expressing (5FdUR_UGI), RTX treated UGI-expressing (RTX_UGI) HCT116 cells; non-treated UGI-expressing (NT_UGI MMR), 5FdUR treated UGI-expressing (5FdUR_UGI MMR), RTX treated UGI-expressing (RTX_UGI MMR) MMR proficient version of HCT116 cells, and non-treated wild-type K562 cells (K562). Genomic DNA was isolated and sonicated to about 300 kb fragments (input), uracil-DNA was enriched by immunoprecipitation via FLAG-tagged U-DNA sensor (enriched). Here, we included K562 data too that was addressed to have a kind of reference point to the previously published dU-seq data (Shu et al., 2018) with which detailed comparison is also made in the Appendix 1.

sample	replicates	number of raw reads	number of mapped reads	unmapped reads		uniquely mapped reads		uniquely mapped reads after blacklisting	
				number	%	number	%	number	%
**WT input**	WT1_son	138283424	138113944	169480	0.12	131604925	95.17	126302380	91.34
	WT2_son	185174607	184959442	215165	0.12	175302618	94.67	168698159	91.10
**WT enriched**	WT1_IP	144612745	144094135	518610	0.36	138611548	95.85	131765827	91.12
	WT2_IP	159514985	159314208	200777	0.13	152796029	95.79	145489972	91.21
**NT_UGI input**	NT1_son	164023406	163757733	265673	0.16	156045404	95.14	149734348	91.29
	NT2_son	173254485	173088530	165955	0.10	165373102	95.45	158978819	91.76
**NT_UGI enriched**	NT1_IP	260763674	260300247	463427	0.18	251164014	96.32	239327438	91.78
	NT2_IP	136148357	134759365	1388992	1.02	129486254	95.11	123064064	90.39
**5FdUR_UGI input**	5FdUR1_son	128706895	128669770	37125	0.03	122476766	95.16	118558597	92.12
	5FdUR1_son	201926203	201560665	365538	0.18	193086643	95.62	184756297	91.50
**5FdUR_UGI enriched**	5FdUR1_IP	150596242	150522522	73720	0.05	144554269	95.99	141582874	94.01
	5FdUR2_IP	138651760	138410833	240927	0.17	133200761	96.07	128584894	92.74
**RTX_UGI input**	RTX1_son	145920877	145775676	145201	0.10	139168642	95.37	133567232	91.53
	RTX2_son	147882518	147674678	207840	0.14	141097936	95.41	135259752	91.46
**RTX_UGI enriched**	RTX1_IP	166544868	166305588	239280	0.14	160567280	96.41	155171205	93.17
	RTX2_IP	151875638	151666578	209060	0.14	146619425	96.54	141987664	93.49
**NT_UGI MMR input**	NT1MMR_son	176769886	176519499	250387	0.14	168384253	95.26	162316924	91.82
	NT2MMR_son	158422442	158204670	217772	0.14	150145829	94.78	144327780	91.10
**NT_UGI MMR enriched**	NT1MMR_IP	206717745	206322712	395033	0.19	198774470	96.16	189830957	91.83
	NT2MMR_IP	181222656	180978162	244494	0.13	174061142	96.05	167043578	92.18
**5FdUR_UGI MMR input**	5FdUR0MMR_son	225701020	225256603	444417	0.20	215868115	95.64	206203797	91.36
	5FdUR1MMR_son	161595292	161314811	280481	0.17	153899974	95.24	147556558	91.31
	5FdUR2MMR_son	168394046	168247239	146807	0.09	160551391	95.34	153742056	91.30
**5FdUR_UGI MMR enriched**	5FdUR0MMR_IP	163350647	163119913	230734	0.14	156865033	96.03	152306214	93.24
	5FdUR1MMR_IP	165059439	164692746	366693	0.22	157148267	95.21	152505075	92.39
	5FdUR2MMR_IP	161660950	161500117	160833	0.10	154724245	95.71	150031196	92.81
**RTX_UGI MMR input**	RTX1MMR_son	182107737	181930877	176860	0.10	173346807	95.19	165477472	90.87
	RTX2MMR_son	216039165	215831688	207477	0.10	204582815	94.70	195579694	90.53
**RTX_UGI MMR enriched**	RTX1MMR_IP	142816751	142519961	296790	0.21	136944112	95.89	133722720	93.63
	RTX2MMR_IP	166796548	166485737	310811	0.19	159559070	95.66	155107383	92.99
**K562 input**	K562_son	106137622	105875437	262185	0.25	100326105	94.52	97429855	91.8
**K562 enriched**	K562_IP	109490393	109306854	183539	0.17	105310296	96.18	102013265	93.17

Correlation was calculated among bam files using multiBamSummary and plotCorrelation tools of the deepTools package (Ramírez et al., 2016). Pearson correlation coefficients were calculated with 5000 bases bin size between uniquely mapped reads of samples after blacklisting as follows:

```
$ multiBamSummary bins --binSize 5000 -b NAME1.filtered_blacklisted.bam
NAME2.filtered_blacklisted.bam {…} NAMEn.filtered_blacklisted.bam  -o
multiBamSummary_bin5000.npz --scalingFactors
scalingFactors_from_multiBamSummary_bin5000.txt --outRawCounts
raw_counts_from_multiBamSummary_bin5000.csv --ignoreDuplicates --maxFragmentLength
2000 --extendReads -v -p 16
```

```
$ plotCorrelation --corData multiBamSummary_bin5000.npz --corMethod pearson --
whatToPlot heatmap -o multiBamSummary_bin5000_heatmap.png -T multiBamSummary_bin5000 -
-skipZeros --removeOutliers --plotNumbers --colorMap RdPu
```

Pearson correlation coefficients between replicates were measured as follows: WT enriched: 0.92, input: 0.89; NT_UGI enriched: 0.79, input: 0.82; 5FdUR_UGI enriched: 0.87, input: 0.88; RTX_UGI enriched: 0.97, input: 0.89. NT_UGI_MMR enriched: 0.92, input: 0.84; 5FdUR_UGI_MMR enriched: 0.88, input: 0.78; RTX_UGI_MMR enriched: 0.95, input: 0.93. All further data processing and analysis steps were done on the two biological replicates separately, as well as on merged bam files of corresponding replicates. All the results were in good agreement between replicates, so hereafter, in the main figures, we show results for the merged data.

Merging replicates were performed at the level of cleaned aligned reads (filtered_blacklisted.bam files) using samtools merge (H. Li et al., 2009).

```
$ samtools merge -r -1 -c --threads 16
NAME(merged).filtered_blacklisted.non_sorted.bam NAME(rep1).filtered_blacklisted.bam
NAME(rep2).filtered_blacklisted.bam
```

```
$ samtools sort -l1 -o NAME(merged).filtered_blacklisted.bam -O BAM -@16
NAME(merged).filtered_blacklisted.non_sorted.bam
```

```
$ samtools index NAME(merged).filtered_blacklisted.bam
```

Comparison of the samples at the level of merged, filtered and blacklisted bam files (Figure 2-figure supplement 3) shows clear differences among input and enriched files, as well as treated and non-treated samples. All input files belong to the HCT116 cell line are quite similar, while the input sample of K562 cells shows significant difference that is another argument for cell type specific blacklisting.

Determination of uracil enrichment: log2 ratio track and derived regions versus peaks called by MACS2 tool.

Uracil enrichment should be determined from the increased coverage of enriched data versus the input using cleaned aligned reads (filtered_blacklisted.bam files), as it is also recommended by the current

ENCODE standard (https://www.encodeproject.org/chip-seq/histone/#restrictions). For that, basically two main ways are available: 1) conventional peak calling algorithms (e.g. MACS2 (Feng et al., 2011; Zhang et al., 2008)), especially if relatively intense and sharp peaks of enrichment are expected; 2) calculation and comparison of genome scaled coverage tracks for both enriched and input sequencing data e.g. in the form of log2 ratio tracks (Figure 3-figure supplement 1). This latter option results in more detailed information on the enrichment in the format of bedGraph or bigwig (bw). However, such log2 ratio tracks (bw files) can hardly be used to screen large databases for colocalizing genomic features or factor binding profiles (cf. Figure 2-figure supplement 1).

In case of the present samples (either non-treated or treated by thymidylate biosynthesis inhibitors), we found broad genomic regions with elevated log2 signals rather than intense sharp peaks (Figure 3A, Figure 3-figure supplement 1, Figure 4-figure supplement 2). Hence, we decided to derive interval (bed) files from the log2 ratio tracks (bw) using a threshold reasonable based on log2 ratio signal histograms (cf. Figure 3C, and Figure 3-figure supplement 4). These intervals might be able to describe such broad regions of uracil enrichment better than the peak calling results (cf. Figure 3-figure supplement 1), and simultaneously allow efficient screening of large datasets for colocalizing features.

To further access the appropriate approach of data processing and extracting information on genomic uracil enrichment, we performed both 1) broad peak calling, and 2) extraction of even broader regions based on log2 ratio tracks. Hereafter, the two terms ’peak’ and ’region’ will be consequently applied for the results of these two approaches, respectively.

1) Peak calling was performed using broad peak option in MACS2 at two different broad-cutoff values (grey intervals at Figure 3-figure supplement 1). Note that --cutoff-analysis option can also be used to estimate the number and length of the peaks at different q and p cutoff values.

```
$ MACS2 callpeak -t NAME(IP).filtered_blacklisted.bam -c
NAME(son).filtered_blacklis.bam --broad -g 2793842910 --broad-cutoff 0.05 -n NAME.0p05
--outdir {PATH} --nomodel -f BAMPE
```

```
$ MACS2 callpeak -t NAME(IP).filtered_blacklisted.bam -c
NAME(son).filtered_blacklis.bam --broad -g 2793842910 --broad-cutoff 0.5 -n NAME.0p5 -
-outdir {PATH} --nomodel -f BAMPE
```

2) Determination of broad regions based on log2 ratio tracks was performed as follows using bamCoverage and bigwigCompare tools of deepTools package (Ramírez et al., 2016), some tools from the kentUtils package of the UCSC (Kuhn et al., 2013), R and linux command-line utilities.

```
$ bamCoverage -b NAME.filtered_blacklisted.bam -o NAME.bin100bp.smooth5000.RPGC.bw --
binSize 100 --verbose --smoothLength 5000 --normalizeUsing RPGC --effectiveGenomeSize
2793842910 -p 16 --extendReads
```

```
$ bigwigCompare -b1 NAME(IP).bin100bp.smooth5000.RPGC.bw -b2
NAME(son).bin100bp.smooth5000.RPGC.bw -o NAME.bin100bp.smooth5000.RPGC.log2.bw -of
bigwig --binSize 100 -v -p 16
```

```
$ bigWigToWig NAME.bin100bp.smooth5000.RPGC.log2.bw
NAME.bin100bp.smooth5000.RPGC.log2.wig
```

In R (Figure 3C, and Figure 3-figure supplement 4):

```
> NAME(short) <- read.delim("NAME.bin100bp.smooth5000.RPGC.log2.wig", header=FALSE)
> hist(NAME(short)$V4, breaks = 100, xlim = c(-1.5, 1.5), ylim = c(0, 2500000))
```

The histograms are shown in Figure 3C, and Figure 3-figure supplement 4, and data are provided in the corresponding source data files. The applied thresholds are shown in Figure 3-figure supplement 2A and also indicated in the corresponding source data files.

Extraction of the data bins with log2 ratio signal higher than the threshold was done as follows:

# deleting lines that is only for indication the bedGraph sections and then selecting data bins that are above the threshold (in this example, it is 0.2)

```
$ grep -vwF "bedGraph" NAME.bin100bp.smooth5000.RPGC.log2.wig | awk ' $4 > 0.2 ' >
NAME.bin100bp.smooth5000.RPGC.log2.0p2.bed
```

# merging neighboring data bins to a single interval, then sorting, then printing column 1, 2, and 3, and also the line number in each line of the bed file

```
$ bedtools merge -i NAME.bin100bp.smooth5000.RPGC.log2.0p2.bed | sort -k1,1 -k2,2n |
awk '{print $1 "\t" $2 "\t" $3 "\t" NR}' >
NAME.bin100bp.smooth5000.RPGC.log2.0p2.numbered.bed
```

# calculating average log2 uracil enrichment value for the intervals in the bed file, it is added to the column 5

```
$ bigWigAverageOverBed -bedOut=NAME.bin100bp.smooth5000.RPGC.log2.0p2.scored.bed
NAME.bin100bp.smooth5000.RPGC.log2.bw
NAME.bin100bp.smooth5000.RPGC.log2.0p2.numbered.bed DEL.tab
```

# sorting, then printing again with the right format of the float numbers in the column 5

```
$ sort -k1,1 -k2,2n NAME.bin100bp.smooth5000.RPGC.log2.0p2.scored.bed | awk '{printf
"%s\t", $1; printf "%s\t", $2; printf "%s\t", $3; printf "%s\t", $4; printf "%f\n",
$5}'  > NAME.bin100bp.smooth5000.RPGC.log2.0p2.region.bed
```

# only if top ranked intervals have to be selected: sorting by average log2 uracil enrichment scores in decreasing order, then selecting the top 50000 intervals (other numbers of top intervals can be defined as it is desired), then sorting back in alphabetic order (that is required by several possible further applications e.g. bedtools)

```
$ sort -k 5 -nr NAME.bin100bp.smooth5000.RPGC.log2.0p2.region.bed | head -n 50000 |
sort -k1,1 -k2,2n > NAME.bin100bp.smooth5000.RPGC.log2.0p2.top50k.bed
```

We argue that peak calling using MACS2 is suboptimal for description of distribution of genomic uracil, even if broad peak calling is applied (Figure 3-figure supplement 1). Based on theoretical expectations (cf. main text) as well as on the initial processing of the actual U-DNA-Seq data, we recommend to use the log2 ratio of the genome scaled coverage tracks and the derived regions of uracil enrichment rather than the peak calling approach.

To further strengthen this choice, we made a detailed comparison on the defined regions of uracil enrichment (based on log2 ratio tracks) and the peak calling results (Figure 3-figure supplement 2). A statistics, including the applied thresholds, Jaccard indices between replicates, and the extent of the regions, are shown for region.bed files derived from the log2 ratio tracks (Figure 3-figure supplement 2A). Regarding peak calling, we found, that using the same broad-cutoff parameter, the numbers of called peaks are extremely different (from 35 000 to 250 000) among the samples, even between parallels. This difference in peak numbers does not seem to correlate with the elevated uracil level in treated samples (cf. higher number of peaks in WT and NT_UGI samples than in the treated ones). Using the „--cutoff-analysis" option in MACS2, we tried to harmonize the number of called peaks in different samples using sometimes very different broad-cutoff parameters (Figure 3-figure supplement 2B). Comparing the two statistics for the two approaches, the reproducibility of peak calling was still much worse (cf. Jaccard index values between replicates, in case of peak calling (Figure 3-figure supplement 2B) versus log2 regions of uracil enrichment (Figure 3-figure supplement 2A)). Lower reproducibility of peak calling results in lower descriptive value for the uracil distribution, as it is also reflected in comparison of drug-treated and non-treated samples (Figure 3-figure supplement 2D vs C).

Overlapping bases and Jaccard indices were calculated for the interval files by bedtools jaccard tool as follows:

```
$ bedtools jaccard -a NAME1.bin100bp.smooth5000.RPGC.log2.0p2.region.bed -b
NAME2.bin100bp.smooth5000.RPGC.log2.0p2.region.bed
```

In the QC report of sequencing from Novogene, the GC contents of the sequenced samples were documented. All samples, except for the non-treated enriched ones, were around 42% characteristic for the human genome. However, in case of non-treated enriched samples, the GC content was consequently decreased to around 37%. We were curious, if such difference might occur due to different GC content of the regions enriched in uracils in the non-treated versus drug-treated samples. Indeed, GC contents of regions were decreased to around 33% and increased to about 44-46% in case of non-treated and drug-treated samples, respectively (Figure 3-figure supplement 2A). For comparison, GC content of the not blacklisted and non-masked part of the reference genome was 40.85% ((number of C + number of G) / effective genome size).

GC% was calculated for the interval files of each sample using bedtools nuc tool and awk as follows:

```
$ bedtools nuc -fi GRCh38.d1.vd1.fa -bed
NAME.bin100bp.smooth5000.RPGC.log2.0p2.region.bed | awk '{(sum1+=$8) (sum2+=$11)
(sum3+=$9) (sum4+=$10)} END {print sum1 "\t" sum2"\t" sum3 "\t" sum4}' >>
summary.region.bed.nuc.csv
```

```
$ bedtools nuc -fi GRCh38.d1.vd1.fa -bed NAME1.0p05_peaks.broadPeak | awk
'{(sum1+=$12) (sum2+=$15) (sum3+=$13) (sum4+=$14)} END {print sum1 "\t" sum2"\t" sum3
"\t" sum4}' >> summary.peaks.bed.nuc.csv
```

Based on the comparison reported in Figure 3-figure supplement 2, we decided that log2 ratio tracks and the derived interval files will be used for further analysis. For visualization, IGV views are shown for all the samples (replicates were merged) in a selected genomic region (Figure 3A), as well as for all the chromosomes (Supplementary file 2).

Furthermore, we used multiBigwigSummary and plotCorrelation to show Pearson correlation on log2 ratio tracks (see the command lines below). Heatmaps for individual replicates (Figure 3-figure supplement 3) and also for merged replicates (Figure 3B) revealed that the treated and non-treated enriched samples are well separated in terms of global uracil distribution pattern.

```
$ multiBigwigSummary bins -b NAME1.filtered_blacklisted.bw
NAME2.filtered_blacklisted.bw {…} NAMEn.filtered_blacklisted.bw -o
mbws_filtered_blacklisted_bw_data.npz -v -p 16
```

```
$ plotCorrelation --corData mbws_filtered_blacklisted_bw_data.npz --corMethod pearson
--whatToPlot heatmap -o mbws_filtered_blacklisted_bw_heatmap.png -T
mbws_filtered_blacklisted_bw --skipZeros --removeOutliers --plotNumbers --colorMap
RdPu
```

For the negative control IP samples, genome-scaled coverage tracks were also calculated in the same way as described above. Then the control signal tracks were normalized according to the amounts of the pulled down DNA (measured by Qubit assay, Figure 1-figure supplement 2A), and were subtracted from their corresponding U-DNA-IP tracks as follows.

```
$ bigwigCompare -b1 5FdUR_UGI_IP.bin100bp.smooth5000.RPGC.bw -b2
5FdUR_UGI_ctr.bin100bp.smooth5000.RPGC.bw --operation subtract -o
5FdUR_UGI_IP_subtract_ctr. bin100bp.smooth5000.RPGC.bw -of bigwig --binSize 100 --
scaleFactors 1:0.109 -v -p 32
```

These corrected coverage tracks were then combined with their input to calculate log2 enrichment tracks (cf. Figure 1-figure supplement 2).

```
$ bigwigCompare -b1 5FdUR_UGI_IP_subtract_ctr.bin100bp.smooth5000.RPGC.bw -b2
5FdUR_UGI son.bin100bp.smooth5000.RPGC.bw -o
5FdUR_UGI_ctr_subtracted.bin100bp.smooth5000.RPGC.log2.bw -of bigwig --binSize 100 -v
-p 32
```

References

Amemiya, H. M., Kundaje, A., & Boyle, A. P. (2019). The ENCODE Blacklist: Identification of Problematic Regions of the Genome. *Scientific Reports*, *9*(1), 9354. https://doi.org/10.1038/s41598-019-45839-z

Bolger, A. M., Lohse, M., & Usadel, B. (2014). Trimmomatic: a flexible trimmer for Illumina sequence data. *Bioinformatics (Oxford, England)*, *30*(15), 2114–2120. https://doi.org/10.1093/bioinformatics/btu170

Buske, O. J., Hoffman, M. M., Ponts, N., Le Roch, K. G., & Noble, W. S. (2011). Exploratory analysis of genomic segmentations with Segtools. *BMC Bioinformatics*, *12*(1), 415. https://doi.org/10.1186/1471-2105-12-415

Chan, R. C. W., Libbrecht, M. W., Roberts, E. G., Bilmes, J. A., Noble, W. S., & Hoffman, M. M. (2017). Segway 2.0: Gaussian mixture models and minibatch training. *Bioinformatics*, *34*(4), 669–671. https://doi.org/10.1093/bioinformatics/btx603

Feng, J., Liu, T., & Zhang, Y. (2011). Using MACS to Identify Peaks from ChIP-Seq Data. *Current Protocols in Bioinformatics*, *34*(1), 2.14.1-2.14.14. https://doi.org/10.1002/0471250953.bi0214s34

Gao, G. F., Parker, J. S., Reynolds, S. M., Silva, T. C., Wang, L.-B., Zhou, W., … Noble, M. S. (2019). Before and After: Comparison of Legacy and Harmonized TCGA Genomic Data Commons' Data. *Cell Systems*, *9*(1), 24-34.e10. https://doi.org/10.1016/j.cels.2019.06.006

Hoffman, M. M., Buske, O. J., & Noble, W. S. (2010). The Genomedata format for storing large-scale functional genomics data. *Bioinformatics (Oxford, England)*, *26*(11), 1458–1459. https://doi.org/10.1093/bioinformatics/btq164

Hoffman, M. M., Buske, O. J., Wang, J., Weng, Z., Bilmes, J. A., & Noble, W. S. (2012). Unsupervised pattern discovery in human chromatin structure through genomic segmentation. *Nature Methods*, *9*(5), 473–476. https://doi.org/10.1038/nmeth.1937

Hunter, J. D. (2007). Matplotlib: A 2D graphics environment. *Computing in Science & Engineering*, *9*(3), 90–95.

Jensen, M. A., Ferretti, V., Grossman, R. L., & Staudt, L. M. (2017). The NCI Genomic Data Commons as an engine for precision medicine. *Blood*, *130*(4), 453–459. https://doi.org/10.1182/blood-2017-03-735654

Kuhn, R. M., Haussler, D., & Kent, W. J. (2013). The UCSC genome browser and associated tools. *Briefings in Bioinformatics*, *14*(2), 144–161. https://doi.org/10.1093/bib/bbs038

Layer, R. M., Pedersen, B. S., DiSera, T., Marth, G. T., Gertz, J., & Quinlan, A. R. (2018). GIGGLE: a search engine for large-scale integrated genome analysis. *Nature Methods*, *15*(2), 123–126. https://doi.org/10.1038/nmeth.4556

Li, H., Handsaker, B., Wysoker, A., Fennell, T., Ruan, J., Homer, N., … Durbin, R. (2009). The Sequence Alignment/Map format and SAMtools. *Bioinformatics*, *25*(16), 2078–2079. https://doi.org/10.1093/bioinformatics/btp352

Li, H. W. (2013). Aligning sequence reads, clone sequences and assembly contigs with BWA-MEM. *ArXiv:1303.3997v1 [q-Bio.GN]*. Retrieved from https://www.semanticscholar.org/paper/Aligning-sequence-reads%2C-clone-sequences-and-with-Li/0ee3a1f7a363b16ceda8f1053a8172f051fd8d4c

McKinney, W., & others. (2010). Data structures for statistical computing in python. In *Proceedings of the 9th Python in Science Conference* (Vol. 445, pp. 51–56).

Quinlan, A. R., & Hall, I. M. (2010). BEDTools: a flexible suite of utilities for comparing genomic features.

Bioinformatics, *26*(6), 841–842. https://doi.org/10.1093/bioinformatics/btq033

R Core Team. (2018). R: A language and environment for statistical computing. *R Foundation for Computing, Vienna, Austria. URL Https://Www.R-Project.Org/*.

Ramírez, F., Ryan, D. P., Grüning, B., Bhardwaj, V., Kilpert, F., Richter, A. S., … Manke, T. (2016). deepTools2: a next generation web server for deep-sequencing data analysis. *Nucleic Acids Research*, *44*(W1), W160-5. https://doi.org/10.1093/nar/gkw257

Shu, X., Liu, M., Lu, Z., Zhu, C., Meng, H., Huang, S., … Yi, C. (2018). Genome-wide mapping reveals that deoxyuridine is enriched in the human centromeric DNA. *Nature Chemical Biology*, *14*(7), 680–687. https://doi.org/10.1038/s41589-018-0065-9

Zhang, Y., Liu, T., Meyer, C. A., Eeckhoute, J., Johnson, D. S., Bernstein, B. E., … Liu, X. S. (2008). Model-based Analysis of ChIP-Seq (MACS). *Genome Biology*, *9*(9), R137. https://doi.org/10.1186/gb-2008-9-9-r137
