# Supplementary material for: Genome-wide alterations of uracil distribution patterns in human DNA upon chemotherapeutic treatments
Source: eLife. 2020 Sep 21;9:e60498. doi: 10.7554/eLife.60498 (PMC7505663; doi:10.7554/eLife.60498)
Supplement: Supplementary file 2. [file elife-60498-supp2.pdf]

Supplementary file 2

IGV views of log2 ratio and regions of uracil enrichment on all the chromosomes

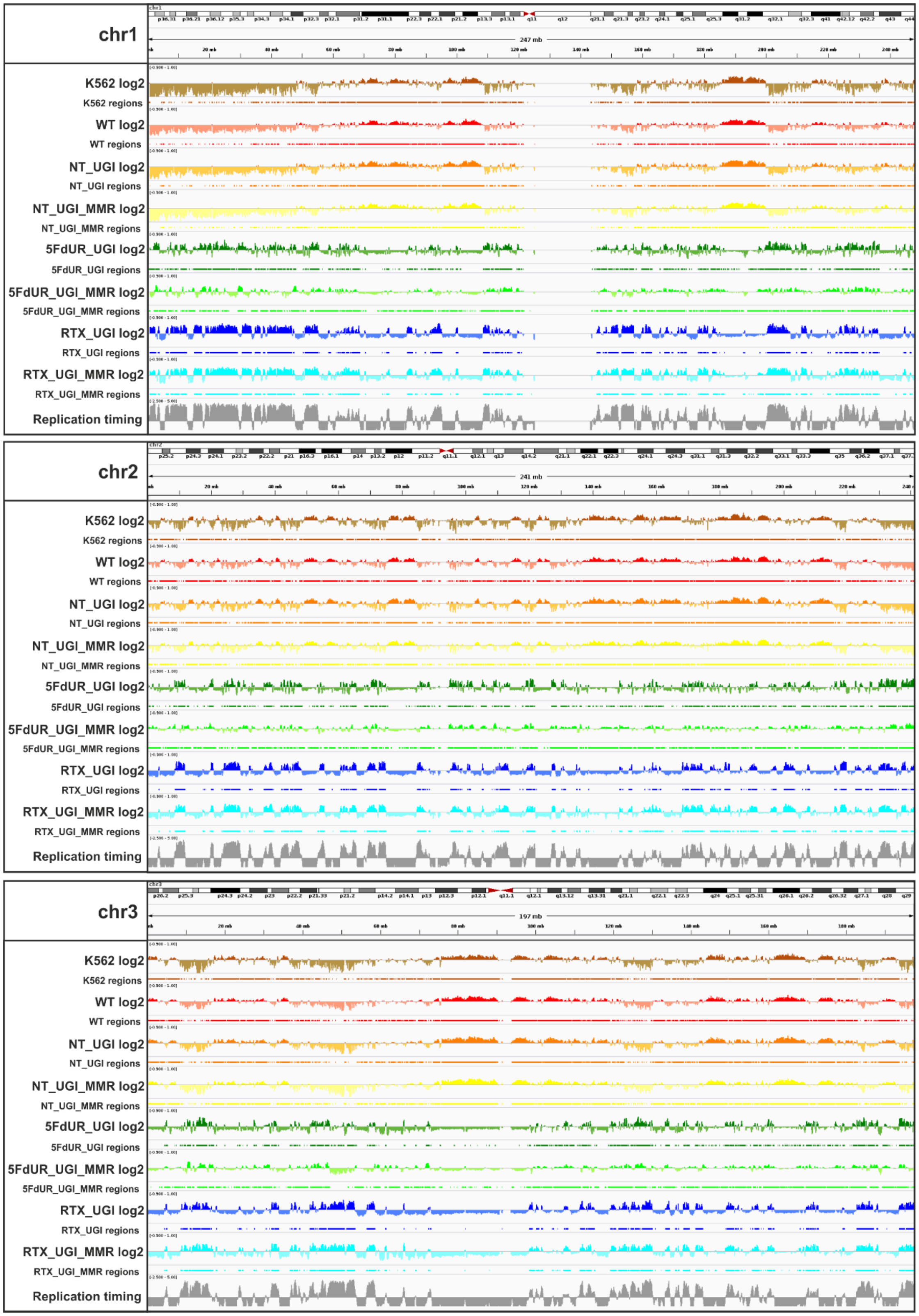

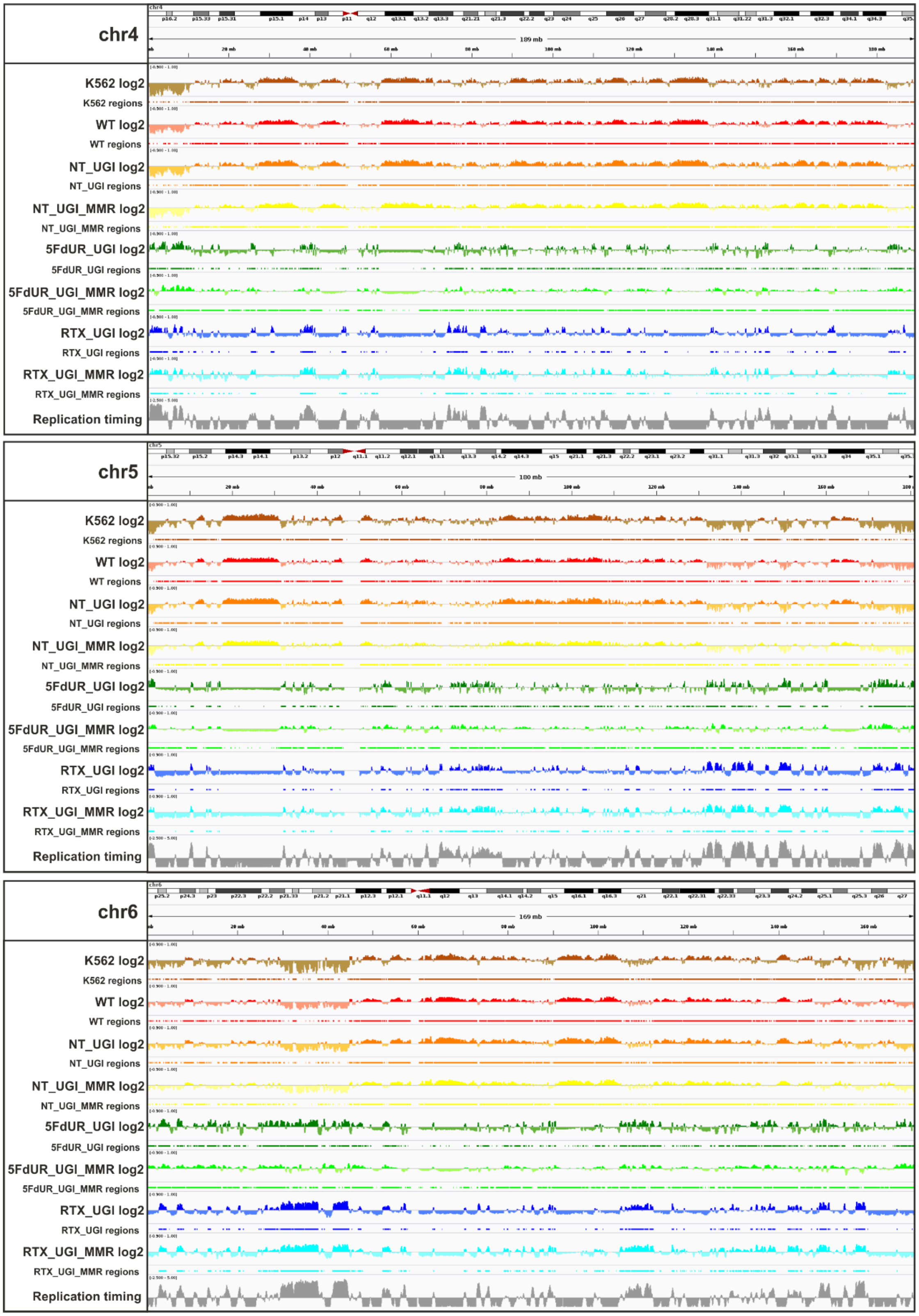
chr4
K562 log2
K562 regions
WT log2
WT regions
NT_UGI log2
NT_UGI regions
NT_UGI_MMR log2
NT_UGI_MMR regions
5FdUR_UGI log2
5FdUR_UGI regions
5FdUR_UGI_MMR log2
5FdUR_UGI_MMR regions
RTX_UGI log2
RTX_UGI regions
RTX_UGI_MMR log2
RTX_UGI_MMR regions
Replication timing
chr5
K562 log2
K562 regions
WT log2
WT regions
NT_UGI log2
NT_UGI regions
NT_UGI_MMR log2
NT_UGI_MMR regions
5FdUR_UGI log2
5FdUR_UGI regions
5FdUR_UGI_MMR log2
5FdUR_UGI_MMR regions
RTX_UGI log2
RTX_UGI regions
RTX_UGI_MMR log2
RTX_UGI_MMR regions
Replication timing
chr6
K562 log2
K562 regions
WT log2
WT regions
NT_UGI log2
NT_UGI regions
NT_UGI_MMR log2
NT_UGI_MMR regions
5FdUR_UGI log2
5FdUR_UGI regions
5FdUR_UGI_MMR log2
5FdUR_UGI_MMR regions
RTX_UGI log2
RTX_UGI regions
RTX_UGI_MMR log2
RTX_UGI_MMR regions
Replication timing

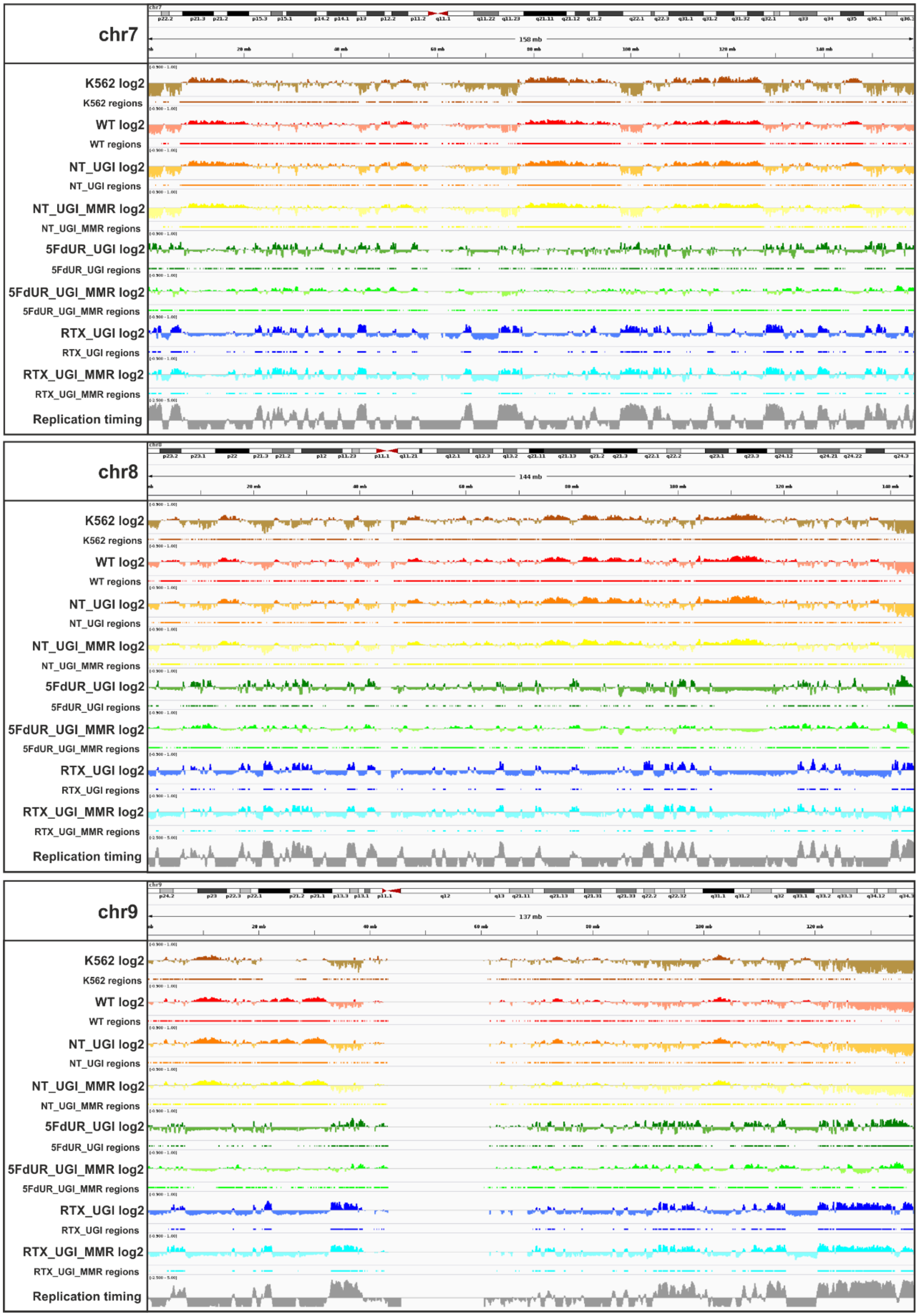
chr7
K562 log2
K562 regions
WT log2
WT regions
NT_UGI log2
NT_UGI regions
NT_UGI_MMR log2
NT_UGI_MMR regions
5FdUR_UGI log2
5FdUR_UGI regions
5FdUR_UGI_MMR log2
5FdUR_UGI_MMR regions
RTX_UGI log2
RTX_UGI regions
RTX_UGI_MMR log2
RTX_UGI_MMR regions
Replication timing
chr8
K562 log2
K562 regions
WT log2
WT regions
NT_UGI log2
NT_UGI regions
NT_UGI_MMR log2
NT_UGI_MMR regions
5FdUR_UGI log2
5FdUR_UGI regions
5FdUR_UGI_MMR log2
5FdUR_UGI_MMR regions
RTX_UGI log2
RTX_UGI regions
RTX_UGI_MMR log2
RTX_UGI_MMR regions
Replication timing
chr9
K562 log2
K562 regions
WT log2
WT regions
NT_UGI log2
NT_UGI regions
NT_UGI_MMR log2
NT_UGI_MMR regions
5FdUR_UGI log2
5FdUR_UGI regions
5FdUR_UGI_MMR log2
5FdUR_UGI_MMR regions
RTX_UGI log2
RTX_UGI regions
RTX_UGI_MMR log2
RTX_UGI_MMR regions
Replication timing

6

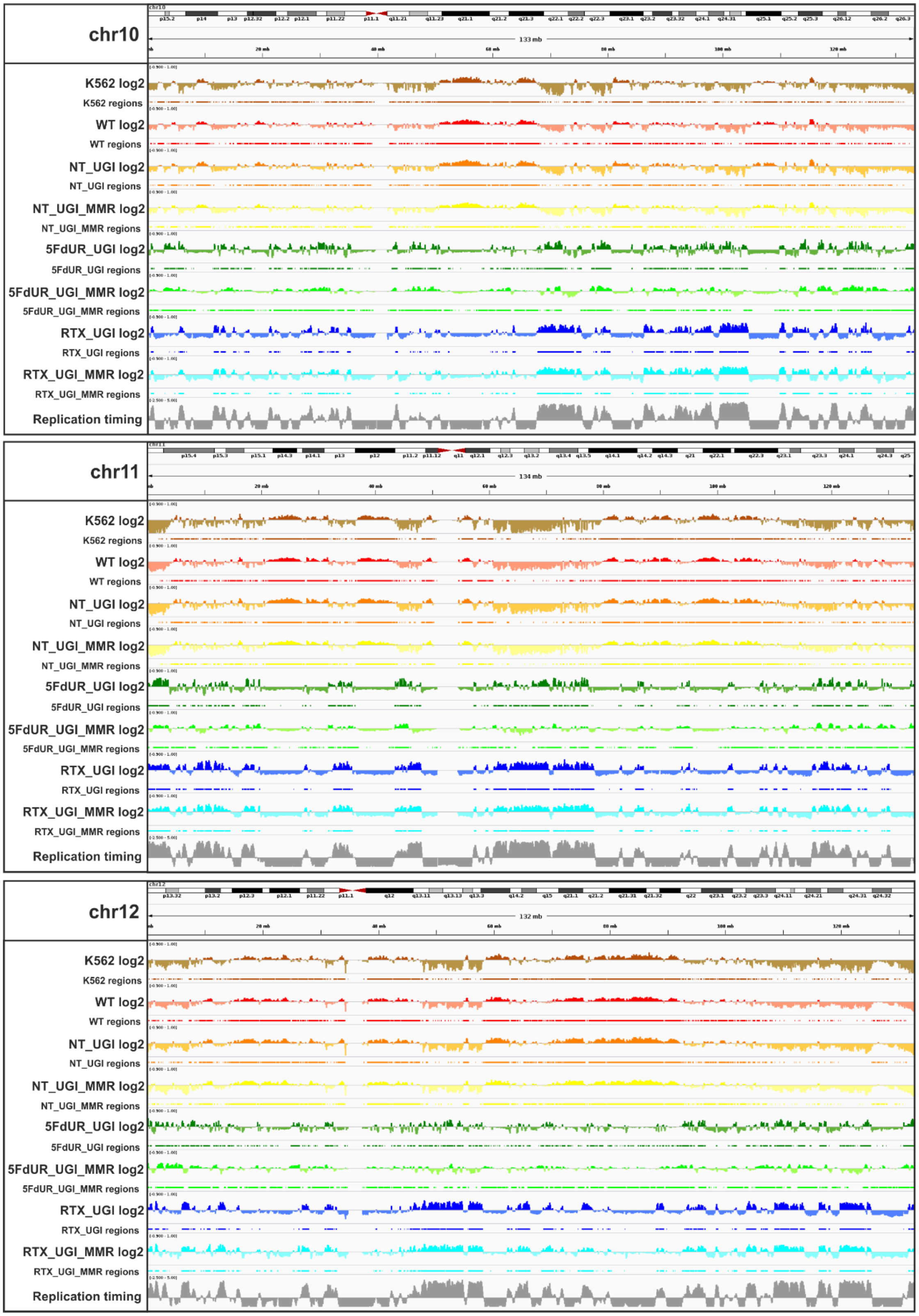
chr10
133 mb
20 mb
40 mb
60 mb
80 mb
100 mb
120 mb
K562 log2
K562 regions
WT log2
WT regions
NT_UGI log2
NT_UGI regions
NT_UGI_MMR log2
NT_UGI_MMR regions
5FdUR_UGI log2
5FdUR_UGI regions
5FdUR_UGI_MMR log2
5FdUR_UGI_MMR regions
RTX_UGI log2
RTX_UGI regions
RTX_UGI_MMR log2
RTX_UGI_MMR regions
Replication timing
chr11
134 mb
20 mb
40 mb
60 mb
80 mb
100 mb
120 mb
K562 log2
K562 regions
WT log2
WT regions
NT_UGI log2
NT_UGI regions
NT_UGI_MMR log2
NT_UGI_MMR regions
5FdUR_UGI log2
5FdUR_UGI regions
5FdUR_UGI_MMR log2
5FdUR_UGI_MMR regions
RTX_UGI log2
RTX_UGI regions
RTX_UGI_MMR log2
RTX_UGI_MMR regions
Replication timing
chr12
132 mb
20 mb
40 mb
60 mb
80 mb
100 mb
120 mb
K562 log2
K562 regions
WT log2
WT regions
NT_UGI log2
NT_UGI regions
NT_UGI_MMR log2
NT_UGI_MMR regions
5FdUR_UGI log2
5FdUR_UGI regions
5FdUR_UGI_MMR log2
5FdUR_UGI_MMR regions
RTX_UGI log2
RTX_UGI regions
RTX_UGI_MMR log2
RTX_UGI_MMR regions
Replication timing

7

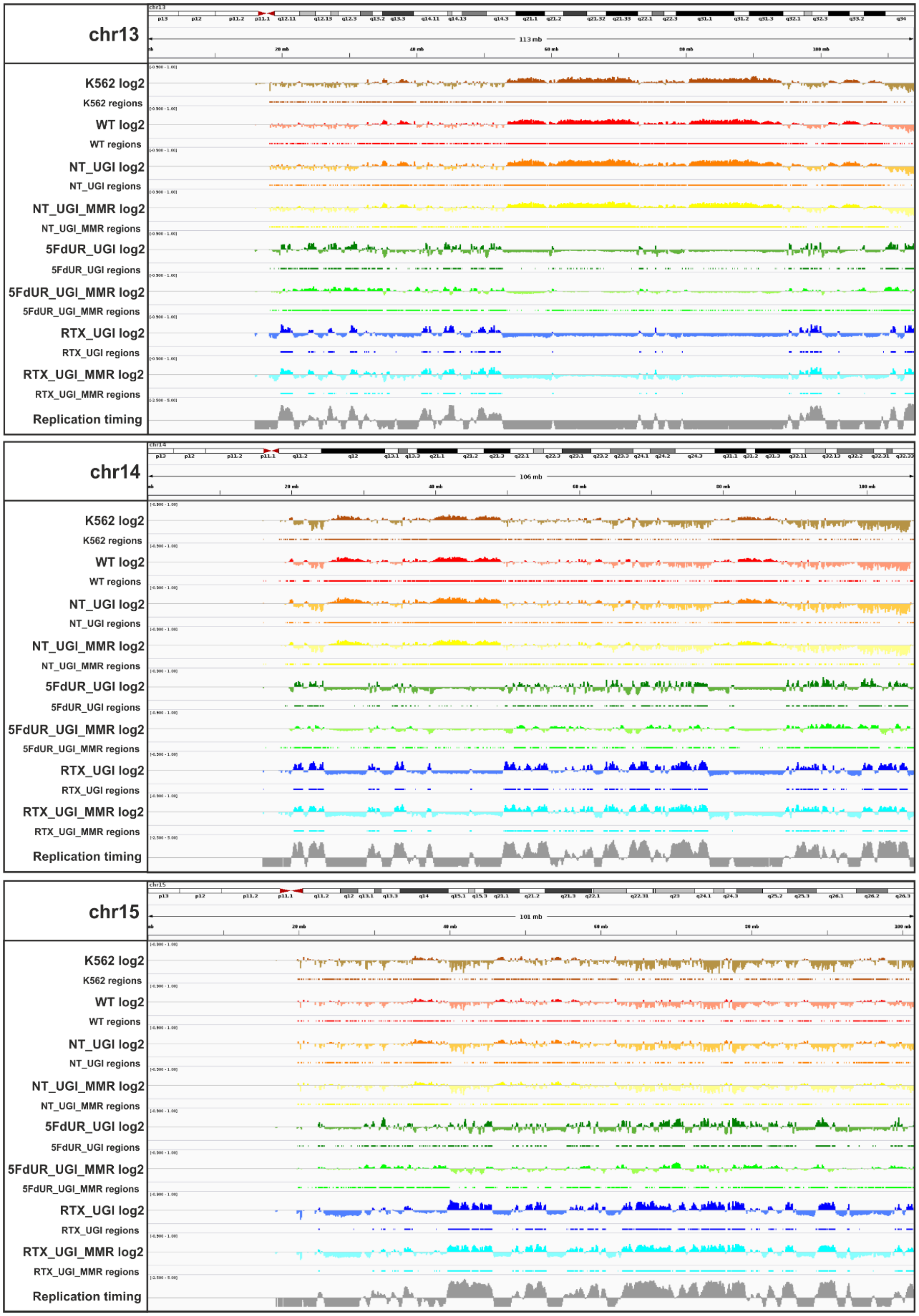
chr13
113 mb
K562 log2
K562 regions
WT log2
WT regions
NT_UGI log2
NT_UGI regions
NT_UGI_MMR log2
NT_UGI_MMR regions
5FdUR_UGI log2
5FdUR_UGI regions
5FdUR_UGI_MMR log2
5FdUR_UGI_MMR regions
RTX_UGI log2
RTX_UGI regions
RTX_UGI_MMR log2
RTX_UGI_MMR regions
Replication timing
chr14
106 mb
K562 log2
K562 regions
WT log2
WT regions
NT_UGI log2
NT_UGI regions
NT_UGI_MMR log2
NT_UGI_MMR regions
5FdUR_UGI log2
5FdUR_UGI regions
5FdUR_UGI_MMR log2
5FdUR_UGI_MMR regions
RTX_UGI log2
RTX_UGI regions
RTX_UGI_MMR log2
RTX_UGI_MMR regions
Replication timing
chr15
101 mb
K562 log2
K562 regions
WT log2
WT regions
NT_UGI log2
NT_UGI regions
NT_UGI_MMR log2
NT_UGI_MMR regions
5FdUR_UGI log2
5FdUR_UGI regions
5FdUR_UGI_MMR log2
5FdUR_UGI_MMR regions
RTX_UGI log2
RTX_UGI regions
RTX_UGI_MMR log2
RTX_UGI_MMR regions
Replication timing

8

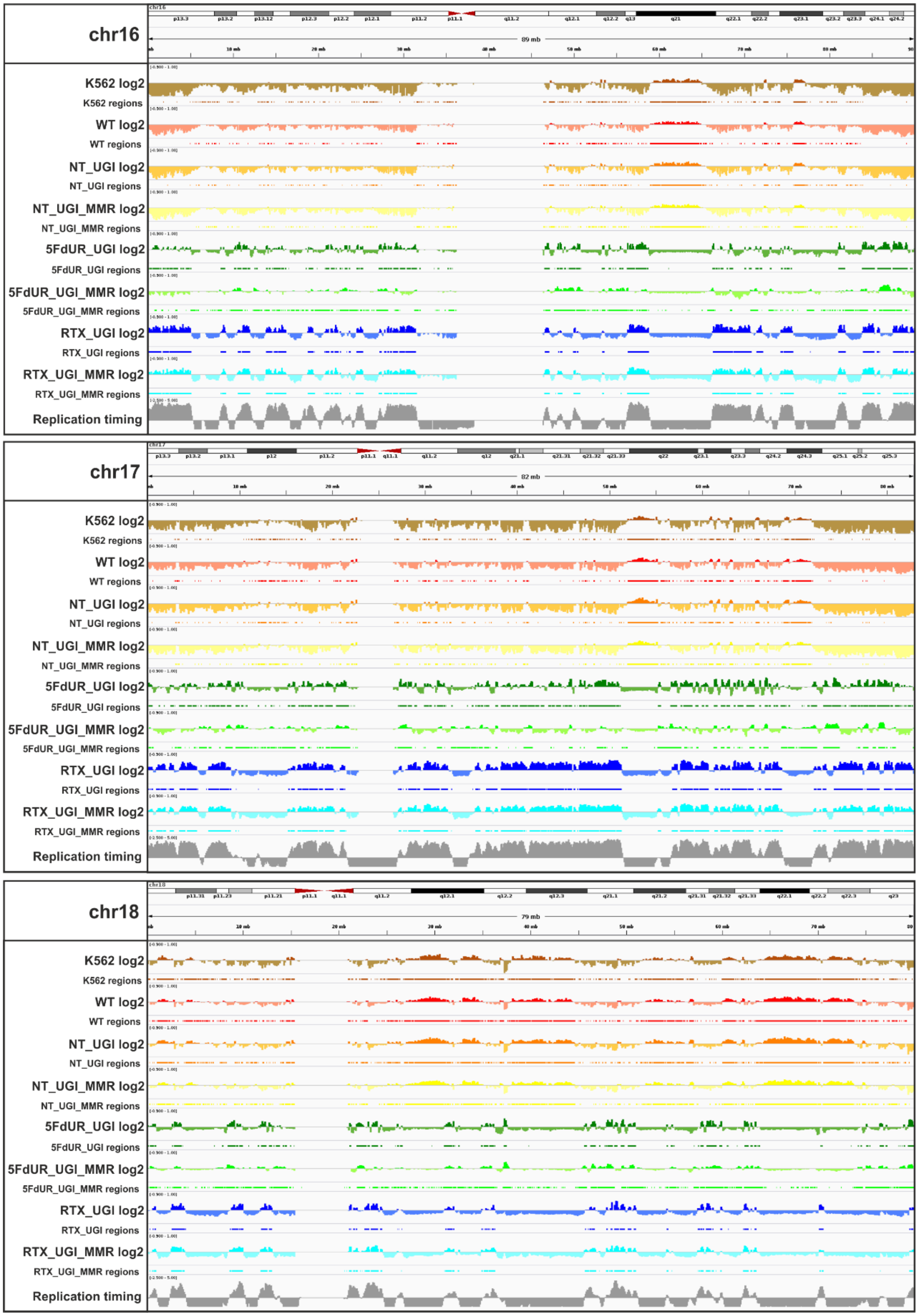
chr16
K562 log2
K562 regions
WT log2
WT regions
NT_UGI log2
NT_UGI regions
NT_UGI_MMR log2
NT_UGI_MMR regions
5FdUR_UGI log2
5FdUR_UGI regions
5FdUR_UGI_MMR log2
5FdUR_UGI_MMR regions
RTX_UGI log2
RTX_UGI regions
RTX_UGI_MMR log2
RTX_UGI_MMR regions
Replication timing
chr17
K562 log2
K562 regions
WT log2
WT regions
NT_UGI log2
NT_UGI regions
NT_UGI_MMR log2
NT_UGI_MMR regions
5FdUR_UGI log2
5FdUR_UGI regions
5FdUR_UGI_MMR log2
5FdUR_UGI_MMR regions
RTX_UGI log2
RTX_UGI regions
RTX_UGI_MMR log2
RTX_UGI_MMR regions
Replication timing
chr18
K562 log2
K562 regions
WT log2
WT regions
NT_UGI log2
NT_UGI regions
NT_UGI_MMR log2
NT_UGI_MMR regions
5FdUR_UGI log2
5FdUR_UGI regions
5FdUR_UGI_MMR log2
5FdUR_UGI_MMR regions
RTX_UGI log2
RTX_UGI regions
RTX_UGI_MMR log2
RTX_UGI_MMR regions
Replication timing

9

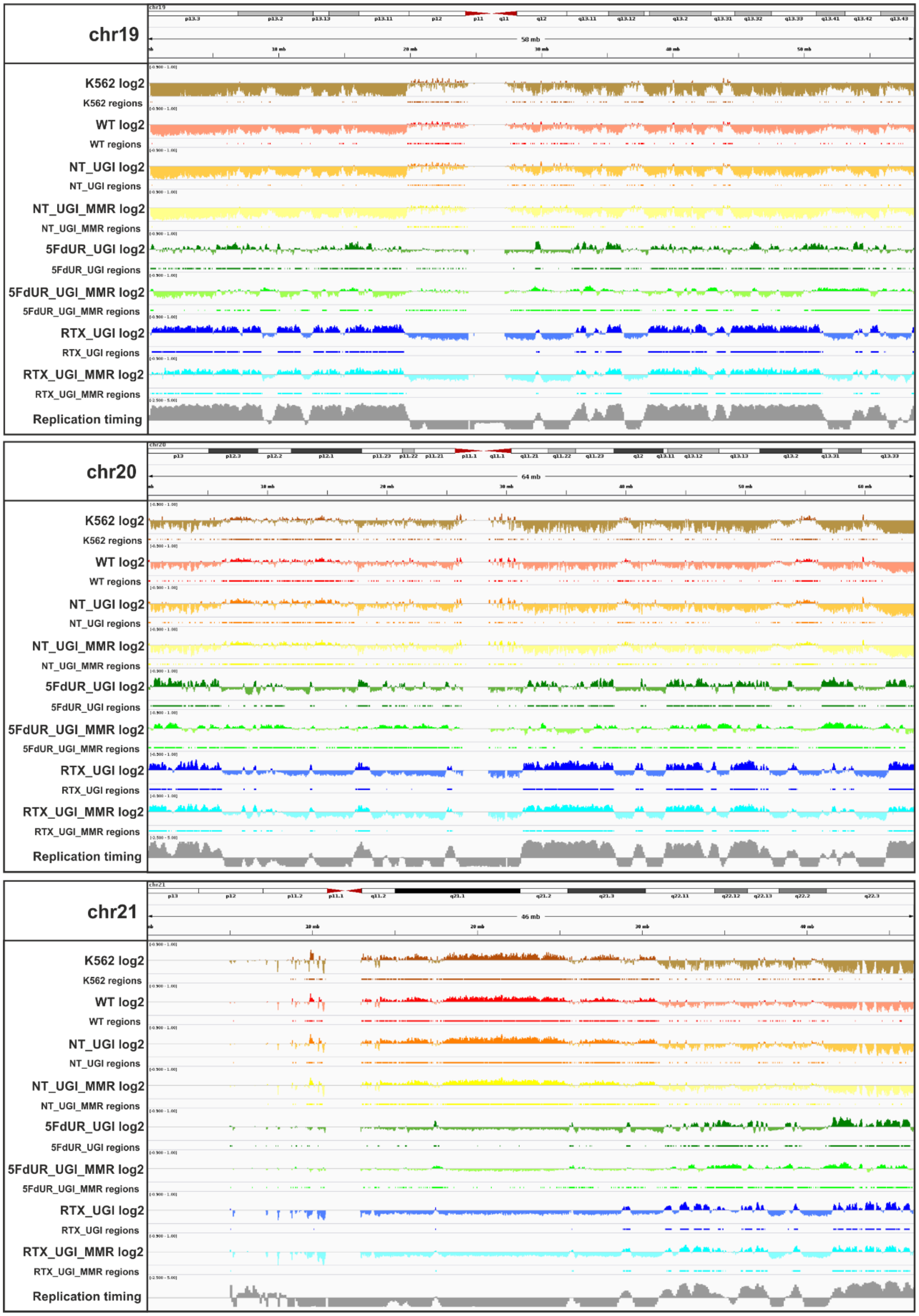
chr19
K562 log2
K562 regions
WT log2
WT regions
NT_UGI log2
NT_UGI regions
NT_UGI_MMR log2
NT_UGI_MMR regions
5FdUR_UGI log2
5FdUR_UGI regions
5FdUR_UGI_MMR log2
5FdUR_UGI_MMR regions
RTX_UGI log2
RTX_UGI regions
RTX_UGI_MMR log2
RTX_UGI_MMR regions
Replication timing
chr20
K562 log2
K562 regions
WT log2
WT regions
NT_UGI log2
NT_UGI regions
NT_UGI_MMR log2
NT_UGI_MMR regions
5FdUR_UGI log2
5FdUR_UGI regions
5FdUR_UGI_MMR log2
5FdUR_UGI_MMR regions
RTX_UGI log2
RTX_UGI regions
RTX_UGI_MMR log2
RTX_UGI_MMR regions
Replication timing
chr21
K562 log2
K562 regions
WT log2
WT regions
NT_UGI log2
NT_UGI regions
NT_UGI_MMR log2
NT_UGI_MMR regions
5FdUR_UGI log2
5FdUR_UGI regions
5FdUR_UGI_MMR log2
5FdUR_UGI_MMR regions
RTX_UGI log2
RTX_UGI regions
RTX_UGI_MMR log2
RTX_UGI_MMR regions
Replication timing

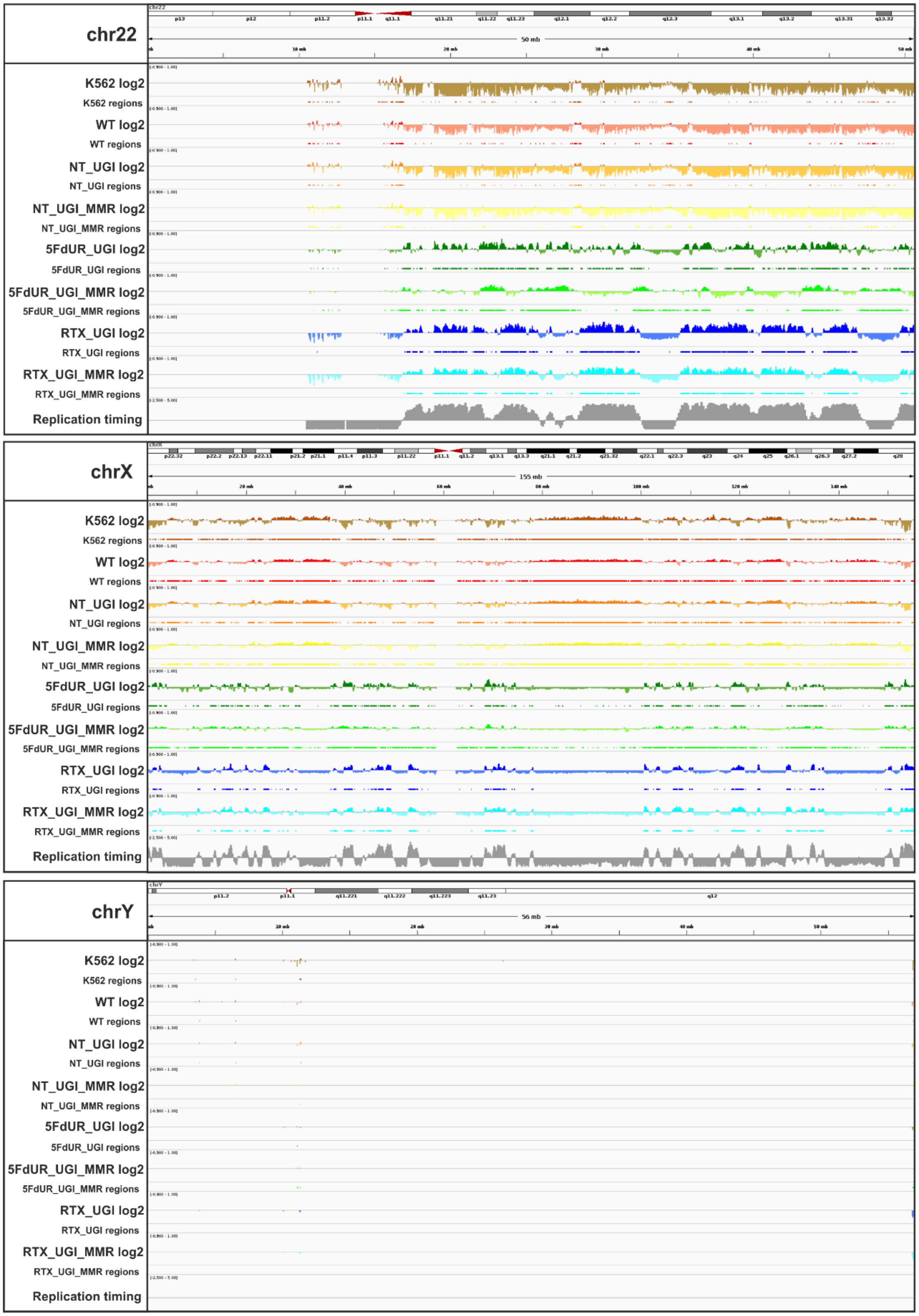
chr22
50 mb
K562 log2
K562 regions
WT log2
WT regions
NT_UGI log2
NT_UGI regions
NT_UGI_MMR log2
NT_UGI_MMR regions
5FdUR_UGI log2
5FdUR_UGI regions
5FdUR_UGI_MMR log2
5FdUR_UGI_MMR regions
RTX_UGI log2
RTX_UGI regions
RTX_UGI_MMR log2
RTX_UGI_MMR regions
Replication timing
chrX
155 mb
K562 log2
K562 regions
WT log2
WT regions
NT_UGI log2
NT_UGI regions
NT_UGI_MMR log2
NT_UGI_MMR regions
5FdUR_UGI log2
5FdUR_UGI regions
5FdUR_UGI_MMR log2
5FdUR_UGI_MMR regions
RTX_UGI log2
RTX_UGI regions
RTX_UGI_MMR log2
RTX_UGI_MMR regions
Replication timing
chrY
56 mb
K562 log2
K562 regions
WT log2
WT regions
NT_UGI log2
NT_UGI regions
NT_UGI_MMR log2
NT_UGI_MMR regions
5FdUR_UGI log2
5FdUR_UGI regions
5FdUR_UGI_MMR log2
5FdUR_UGI_MMR regions
RTX_UGI log2
RTX_UGI regions
RTX_UGI_MMR log2
RTX_UGI_MMR regions
Replication timing

11
