# Supplementary material for: Genome-wide alterations of uracil distribution patterns in human DNA upon chemotherapeutic treatments
Source: eLife. 2020 Sep 21;9:e60498. doi: 10.7554/eLife.60498 (PMC7505663; doi:10.7554/eLife.60498)
Supplement: Supplementary file 3. — Database information, applied command lines, full results of GIGGLE search (table 1), details of our own ChIP-seq (table 2), and list of files for Segway analysis (table 3) are provided. [file elife-60498-supp3.pdf]

Supplementary file 3

Genome-wide analysis of uracil-DNA pattern comparing to ChIP-seq and DNA accessibility data using either GIGGLE search or the Segway genome segmentation tool.

Collection of HCT116 related ChIP-seq and DNA accessibility data.

To find colocalizing binding factors and other genomic features, first a HCT116 specific or relevant set of data were collected. On the one hand, from Cistrome database (http://cistrome.org/db/#/, (Mei et al., 2017), data reflect the state of 17 July 2019), overall, 542 ChIP-seq data made in HCT116 for transcription factors or histone markers were downloaded as interval (bed) files. Although, these data are still heterogeneous regarding quality and the applied treatments, it is definitely more reasonable than searching in the whole Cistrome database without any restriction for cell types. Only those data were comprised that contained more than 400 intervals (471 files remained). It also has to be noted that no controls (such as input samples in our case) are considered in the evaluation pipeline of Cistrome. To strengthen this dataset, further HCT116-specific ChIP-seq data (36 bed files) were downloaded from ENCODE (https://www.encodeproject.org/search/?type=Experiment&status=released&replicates.library.biosample.donor.organism.scientific_name=Homo+sapiens&assembly=GRCh38&biosample_ontology.classification=cell+line&biosample_ontology.term_name=HCT116 (ENCODE Project Consortium, 2012)), where evaluation pipeline (https://www.encodeproject.org/pages/pipelines/) considers controls and includes many quality measures, and the resulted "replicated peaks" reflect only the consensus peaks of replicates and pseudo-replicates. Further 27 bed files were downloaded from the Ensembl database ((Zerbino et al., 2018), release 97, July 2019, ftp://ftp.ensembl.org/pub/release-97/regulation/homo_sapiens/Peaks/HCT116/). Moreover, for colorectal tissues, comprehensive epigenomic data focusing on five core histone marks (H3K4me3, H3K4me1, H3K27me3, H3K9me3, and H3K36me3) were constructed by Roadmap Epigenomics (https://egg2.wustl.edu/roadmap/web_portal/processed_data.html, (Kundaje et al., 2015)). From these data, overall 40 bed files (broad- and gappedPeaks) corresponding to E075 Colonic Mucosa (7 experiments); E076: Colon Smooth Muscle (7 experiments); and E106: Sigmoid Colon (6 experiments)) were also integrated to our dataset for comparison with U-DNA-Seq data. These files were originally aligned to the hg19 reference genome, therefore liftOver (https://github.com/ucscGenomeBrowser/kent) was applied to convert the coordinates to hg38 as follows.

```
$ liftOver NAME_of_DB_intervals.bed hg19ToHg38.over.chain
NAME_ofDB_intervals_hg38.bed unMapped
```

# The file hg19ToHg38.over.chain can be downloaded from the UCSC (http://hgdownload.soe.ucsc.edu/goldenPath/hg19/liftOver/hg19ToHg38.over.chain.gz).

Finally, to address the published centromeric localization of uracil (Shu et al., 2018), ChIP-seq data on CENPA in HuRef cells were also downloaded (GSM1105684, bw file (Hayden et al., 2013)). Note that CENPA data are not available for HCT116 cells. In this study, reads were aligned to human reference genome hg19, and enrichment was given in bedgraph format containing only the alpha satellite segments. Following the paper (Hayden et al., 2013), those data bins were selected that showed at least two-fold enrichment, and then bed files were generated (using the same procedure that we used for derivation of region interval files from the log2 coverage tracks). Then liftOver was applied to convert hg19 coordinates to hg38 (GSM1105684_2fold_enriched_merged_hg38.bed). Data from the same experiment appear also in the Cistrome database (40153), where data were simply realigned to hg38 reference genome, although the original paper reported much more careful procedure on mapping reads in the highly repetitive centromeres (Hayden et al., 2013). Other CENPA data in Cistrome database represent results on ectopically expressed CENPA, outside of the centromeres.

GIGGLE search with U-DNA-Seq regions in the established HCT116 related ChIP-seq dataset.

After neglecting files that contain less than 400 intervals, overall, 576 bed files remained in the dataset. Because of the limitation of the applied GIGGLE search tool ((Layer et al., 2018) version 1.0, cf. issue #46 https://github.com/ryanlayer/GIGGLE/issues/46), top 100,000 intervals were selected from those interval (bed) files that contained more than 100,000 intervals (overall 50 bed files were cut in this way).

Then GIGGLE search was performed on this set of relevant and good quality data with all the U-DNA-Seq samples corresponding to HCT116 cells. A digestion of the results is shown in Figure 4A, while the whole set of the combo scores is provided in Supplementary file 3-table 1. The GIGGLE search was done as follows:

# The database interval files as well as the query interval files have to be sorted and gzipped using a script belongs to the GIGGLE package using also bgzip tool that has to be installed in advance (https://github.com/samtools/htslib/releases/, htslib-1.9.tar.bz2).

```
$  {PATH}/GIGGLE/scripts/sort_bed "{PATH}/*.bed" bed_sorted 4
$  {PATH}/GIGGLE/scripts/sort_bed "{PATH}/*.region.bed" own_bed_sorted 4
```

# Indexing the database

```
$ GIGGLE index -i "bed_sorted/*gz" -o bed_sorted_b -f -s
```

# Running GIGGLE search in this indexed library

```
$ GIGGLE search -i bed_sorted_b -q
own_bed_sorted/NAME.filtered_blacklisted.bin100bp.smooth5k.RPGC.log2.0p2.region.bed.
gz -s > NAME.log2.0p2.regions.GIGGLE_results.csv
```

Note: In some cases, GIGGLE search might end in an error message “too many open files”. To solve this problem, a soft limit (-Sn) of the possible open files has to be checked and changed on the linux operating system (it is possible to do up to the hard limit (-Hn)).

# Checking:

```
$ ulimit -Sn
$ ulimit -Hn
```

# Changing:

```
$ ulimit -Sn 4096
```

ChIP-seq factors corresponding to the 10 best correlating hits for each U-DNA-Seq sample were selected. GIGGLE scores between all seven samples and all experiments corresponding to these factors were plotted excluding those, where data were found not informative (CNOT3, H2B, H3K27me1/2, SKP2, SIRT1, MCM2, and H4K20me1). All data are provided here in Supplementary file 3-table 1.

Supplementary file 3-table 1. Combo scores from GIGGLE search on the full dataset.

factor	WT regions	NT_UGI regions	NT_UGI MMR regions	5FdUR_UGI regions	5FdUR_ UGI MMR regions	RTX_ UGI regions	RTX_UGI MMR regions	number of intervals	primary DB	primary_DB ID
histone markers										
H2A	-662.4	-681.8	-663.3	386.1	0.0	338.6	6.8	16198	Cistrome	51143
H2A	-805.1	-855.3	-866.5	342.7	-11.3	316.8	0.0	7396	Cistrome	51159
H2AFZ	-411.8	-422.5	-414.6	443.7	105.7	375.9	226.6	16633	Cistrome	100929
H2AFZ	-463.5	-475.5	-484.8	469.1	199.3	341.5	201.6	12876	Cistrome	100930
H2AFZ	151.7	162.2	150.9	407.3	-5.2	329.8	182.5	70941	ENCODE	ENCFF549VEQ.bed.gz
H2AZ	-569.3	-589.3	-576.5	418.2	66.0	423.6	150.1	56439	Cistrome	84716
H2AZ	-523.2	-541.9	-534.2	414.8	82.3	417.0	140.8	59933	Cistrome	84715
H2AZ	-72.3	-83.5	-81.9	411.4	149.5	352.1	143.2	100000	Cistrome	83065
H2AZ	-735.2	-819.4	-799.8	460.5	81.2	352.4	91.6	16374	Cistrome	84720
H2AZ	-923.8	-943.5	-954.4	437.0	1.3	318.1	2.5	3440	Cistrome	84719
H2AZ	-915.8	-931.1	-997.6	457.5	23.1	322.7	17.8	9478	Cistrome	84717
H2AZ	3.4	5.0	2.3	374.7	143.8	294.7	120.5	100000	Cistrome	88446
H2B	-12.5	-19.7	-14.6	10.3	3.6	1.9	0.0	1007	Cistrome	40190
H2B	0.1	0.1	0.1	0.0	0.0	0.0	-10.0	571	Cistrome	40192
H2Bub	-37.1	-38.9	-40.3	85.0	3.0	202.3	105.4	5475	Cistrome	40191
H3.3	-557.8	-572.0	-578.4	278.7	0.0	298.5	0.2	12756	Cistrome	77204
H3.3	-47.8	-51.8	-46.9	45.0	0.0	14.4	-0.2	1016	Cistrome	74191
H3K18ac	-1057.4	-1029.4	-975.9	311.6	-48.7	346.7	-0.1	15021	Cistrome	81141
H3K18ac	-232.5	-237.2	-184.3	15.4	-17.9	49.7	-0.3	673	Cistrome	84122
H3K18cr	-760.2	-781.0	-751.3	359.1	-0.1	372.9	6.6	27293	Cistrome	88253
H3K18cr	-1026.8	-1007.8	-944.9	307.7	-50.4	335.0	-0.6	12848	Cistrome	88250
H3K18cr	-1204.4	-1189.0	-1113.9	322.3	-38.3	319.3	-1.3	6848	Cistrome	88251
H3K27ac	-775.5	-815.0	-777.1	467.8	154.0	587.3	309.7	100000	Roadm. Epi.	E075-H3K27ac.narrowPeak.bed.gz
H3K27ac	-819.8	-856.9	-821.8	436.3	109.5	575.0	314.2	100000	Roadm. Epi.	E106-H3K27ac.narrowPeak.bed.gz
H3K27ac	-464.0	-473.2	-474.6	278.9	-497.8	580.6	209.9	45508	ENCODE	ENCFF349LKU.bed.gz
H3K27ac	-530.0	-544.7	-540.8	322.6	-7.5	528.8	129.8	40985	ENCODE	ENCFF083ADY.bed.gz
H3K27ac	-470.1	-474.3	-465.8	364.6	164.5	521.8	382.9	100000	Roadm. Epi.	E075-H3K27ac.broadPeak.bed.gz
H3K27ac	-535.0	-553.0	-536.7	344.1	103.8	515.9	374.6	100000	Roadm. Epi.	E106-H3K27ac.broadPeak.bed.gz
H3K27ac	-558.2	-583.1	-551.4	370.5	109.7	482.6	267.1	100000	Roadm. Epi.	E076-H3K27ac.narrowPeak.bed.gz
H3K27ac	-610.4	-615.6	-617.6	361.7	-3.9	450.5	53.7	57085	Cistrome	61965
H3K27ac	-569.6	-570.2	-576.5	281.1	-4.3	460.8	2.9	14808	Ensemble	homo_sapiens.GRCh38.HCT116.H3K27ac.SWEmbl_ R0005.peaks.20190329.bed.gz
H3K27ac	-472.4	-476.7	-466.7	396.7	28.3	450.1	135.8	67892	Cistrome	66813
H3K27ac	-499.3	-505.1	-508.4	318.1	-5.7	437.3	89.2	50049	Cistrome	66991
H3K27ac	-478.8	-488.6	-483.0	294.1	-18.6	429.3	116.2	36552	Cistrome	101737
H3K27ac	-564.6	-564.2	-566.8	362.4	-0.2	417.4	26.3	51101	Cistrome	83014
H3K27ac	-546.5	-553.0	-560.2	315.4	-12.8	413.1	13.9	45759	Cistrome	45272
H3K27ac	-447.0	-450.7	-453.9	310.0	-3.5	414.2	79.3	49495	Cistrome	66992
H3K27ac	-480.1	-486.5	-484.5	306.6	-8.3	411.2	44.5	42374	Cistrome	66993
H3K27ac	-450.7	-454.2	-449.9	299.3	-2.5	402.4	41.8	41740	Cistrome	85866
H3K27ac	-291.8	-295.5	-283.0	267.8	120.1	422.2	333.9	100000	Roadm. Epi.	E076-H3K27ac.broadPeak.bed.gz
H3K27ac	-587.6	-623.9	-611.5	280.8	-18.9	392.1	3.4	35902	Cistrome	61942
H3K27ac	-558.1	-557.6	-573.1	256.2	-82.1	390.6	0.5	22363	Cistrome	101739
H3K27ac	-496.9	-504.2	-503.8	326.4	-0.8	388.6	41.0	39573	Cistrome	83018
H3K27ac	-661.2	-667.2	-675.9	298.8	-25.9	380.4	0.3	36874	Cistrome	62110
H3K27ac	-671.0	-674.9	-678.3	247.1	-154.8	378.7	-0.7	23506	Cistrome	61940
H3K27ac	-631.7	-641.3	-636.2	289.7	-22.6	376.5	0.9	35218	Cistrome	62112
H3K27ac	-466.9	-479.3	-463.8	340.2	0.4	384.7	57.8	45825	Cistrome	85293
H3K27ac	-703.9	-693.4	-765.9	200.8	-40.5	379.0	2.1	4400	Cistrome	85867
H3K27ac	-558.9	-532.3	-571.3	240.3	-108.6	379.2	0.0	24442	Cistrome	61939
H3K27ac	-689.6	-695.2	-694.2	300.6	-40.1	376.0	0.1	32883	Cistrome	62109
H3K27ac	-499.7	-509.6	-511.3	336.3	0.0	380.7	33.4	38279	Cistrome	85283
H3K27ac	-390.9	-398.3	-395.2	271.3	-0.2	379.8	62.6	44200	Cistrome	82335
H3K27ac	-723.4	-702.9	-724.7	334.6	-35.5	367.6	0.0	25172	Cistrome	66757
H3K27ac	-753.2	-747.0	-756.8	254.0	-132.7	365.2	0.0	11136	Cistrome	66393
H3K27ac	-600.3	-582.4	-599.3	265.1	-100.1	365.2	-0.4	20517	Cistrome	42163
H3K27ac	-373.2	-377.1	-377.5	285.0	0.2	372.4	105.9	50279	Cistrome	82334
H3K27ac	-637.2	-656.4	-650.8	279.4	-42.5	363.4	0.0	30630	Cistrome	62111
H3K27ac	-530.6	-536.9	-529.0	343.6	-0.5	371.6	5.8	5418	Cistrome	51146
H3K27ac	-859.8	-819.2	-830.4	302.9	-93.3	359.8	0.0	15455	Cistrome	81343
H3K27ac	-660.4	-652.0	-667.9	297.5	-75.9	360.4	0.2	16665	Cistrome	81342
H3K27ac	-499.7	-496.7	-501.4	275.2	-8.4	365.7	3.4	34708	Cistrome	84767
H3K27ac	-438.4	-434.9	-441.3	266.0	-1.1	362.3	25.4	42466	Cistrome	88909
H3K27ac	-542.9	-531.0	-560.3	300.4	-33.4	359.6	0.1	22842	Cistrome	81344
H3K27ac	-787.2	-797.3	-785.2	285.0	-81.1	356.7	0.0	13243	Cistrome	81345
H3K27ac	-592.3	-577.3	-610.2	269.1	-93.4	351.2	0.0	21201	Cistrome	66394
H3K27ac	-615.0	-632.6	-623.2	294.5	-1.9	359.9	3.3	35090	Cistrome	49529
H3K27ac	-756.6	-740.3	-727.8	251.8	-47.8	354.5	0.0	7375	Cistrome	84766
H3K27ac	-494.7	-481.8	-498.7	241.7	-37.4	351.2	-1.1	24024	Cistrome	61941
H3K27ac	-555.1	-558.2	-574.1	257.2	-66.6	345.0	-0.1	21349	Cistrome	66395
H3K27ac	-485.1	-472.3	-495.8	265.8	-8.5	354.3	16.0	16412	Cistrome	83017
H3K27ac	-763.7	-728.0	-746.1	273.3	-62.9	349.3	-0.1	12964	Cistrome	81347
H3K27ac	-526.3	-560.1	-571.3	292.7	-26.1	346.5	2.5	22907	Cistrome	57096
H3K27ac	-499.5	-511.5	-502.3	277.3	-2.9	347.4	19.7	20922	Cistrome	87277
H3K27ac	-510.7	-504.5	-508.6	296.6	-0.2	346.3	0.4	33159	Cistrome	42164

H3K27ac	-570.8	-566.9	-566.4	274.4	-3.0	351.0	15.5	12793	Cistrome	87276
H3K27ac	-736.3	-729.9	-732.7	259.3	-50.6	336.8	-0.2	13255	Cistrome	85454
H3K27ac	-624.3	-621.5	-624.8	260.6	-31.7	335.7	0.0	13528	Cistrome	81346
H3K27ac	-716.3	-763.0	-748.0	291.3	-4.1	335.8	0.0	23025	Cistrome	49530
H3K27ac	-494.1	-501.5	-499.7	278.0	-6.0	336.6	0.5	25411	Cistrome	85455
H3K27ac	-351.9	-361.4	-395.2	230.4	-3.2	319.6	6.5	6904	Cistrome	87275
H3K27ac	-611.2	-621.6	-615.8	258.6	-3.9	282.1	1.9	7309	Cistrome	87281
H3K27ac	-112.6	-80.9	-88.9	-3.4	-0.3	-21.7	-79.9	876	Cistrome	67551
H3K27me1	10.4	10.2	6.2	-9.7	-59.7	6.2	3.9	3757	Cistrome	69463
H3K27me1	-3.3	-10.7	-6.2	-2.4	-10.7	0.1	-0.3	431	Cistrome	69460
H3K27me1	-2.6	-1.2	-4.3	-1.9	-13.9	0.0	-0.2	574	Cistrome	69466
H3K27me2	3.0	1.5	1.2	-0.1	-0.1	0.0	0.0	1246	Cistrome	69459
H3K27me3	-779.3	-818.1	-799.5	730.4	116.1	484.4	267.5	100000	Roadm. Epi.	E075-H3K27me3.narrowPeak.bed.gz
H3K27me3	-580.5	-607.8	-590.1	507.2	106.9	313.7	199.6	65497	Roadm. Epi.	E076-H3K27me3.narrowPeak.bed.gz
H3K27me3	-395.1	-452.7	-388.1	57.1	-0.1	227.7	12.2	2705	Cistrome	51162
H3K27me3	-405.3	-410.2	-403.1	367.6	135.1	272.8	218.2	100000	Roadm. Epi.	E075-H3K27me3.broadPeak.bed.gz
H3K27me3	-485.6	-509.7	-497.8	469.0	3.9	229.6	171.9	100000	Roadm. Epi.	E106-H3K27me3.narrowPeak.bed.gz
H3K27me3	-281.1	-293.5	-283.9	281.0	115.3	174.7	148.8	100000	Roadm. Epi.	E076-H3K27me3.broadPeak.bed.gz
H3K27me3	-232.9	-234.8	-231.7	194.5	11.0	66.9	96.5	100000	Roadm. Epi.	E106-H3K27me3.broadPeak.bed.gz
H3K27me3	-65.5	-57.5	-64.2	42.5	0.3	30.4	1.6	699	Cistrome	51147
H3K27me3	-159.1	-155.8	-154.0	103.1	ND	-168.7	-76.1	199001	Ensemble	homo_sapiens.GRCh38.HCT116.H3K27me3.ccat_histone.peaks.20190329.mergedd1000.bed.gz
H3K27me3	-279.5	-289.5	-281.1	-31.4	104.2	-341.4	-244.9	100000	ENCODE	ENCFF806AYM.bed.gz
H3K36me3	-499.5	-519.2	-502.5	208.7	105.8	837.8	634.1	100000	Roadm. Epi.	E075-H3K36me3.narrowPeak.bed.gz
H3K36me3	-397.7	-414.4	-409.2	97.1	-23.5	771.3	645.1	100000	Roadm. Epi.	E106-H3K36me3.narrowPeak.bed.gz
H3K36me3	-329.0	-341.3	-339.2	122.7	6.3	740.9	615.4	100000	Roadm. Epi.	E076-H3K36me3.narrowPeak.bed.gz
H3K36me3	-261.3	-269.3	-272.3	48.6	8.7	717.1	599.4	72341	ENCODE	ENCFF029GQD.bed.gz
H3K36me3	-186.6	-188.5	-190.7	44.8	-164.0	530.2	502.2	43916	Ensemble	homo_sapiens.GRCh38.HCT116.H3K36me3.ccat_histone.peaks.20190329.mergedd1000.bed.gz
H3K36me3	-202.0	-207.3	-206.0	182.7	32.8	491.3	419.9	100000	Roadm. Epi.	E076-H3K36me3.broadPeak.bed.gz
H3K36me3	-246.2	-251.3	-250.5	191.1	106.7	492.8	416.4	100000	Roadm. Epi.	E075-H3K36me3.broadPeak.bed.gz
H3K36me3	-194.0	-198.7	-203.2	77.7	-0.3	462.1	439.6	100000	Roadm. Epi.	E106-H3K36me3.broadPeak.bed.gz
H3K36me3	-35.1	-44.2	-37.0	2.7	-22.3	137.8	32.1	563	Cistrome	51149, 51164
H3K4me1	-1067.6	-1119.8	-1058.1	573.3	28.6	718.4	331.8	100000	Roadm. Epi.	E075-H3K4me1.narrowPeak.bed.gz
H3K4me1	-457.7	-477.3	-463.7	438.2	ND	712.2	290.8	100000	ENCODE	ENCFF986BGX.bed.gz
H3K4me1	-446.2	-456.4	-449.3	331.5	6.7	629.8	185.5	99735	Ensemble	homo_sapiens.GRCh38.HCT116.H3K4me1.ccat_histone.peaks.20190329.bed.gz
H3K4me1	-418.0	-426.3	-419.4	445.5	90.2	590.5	246.2	100000	Cistrome	83177
H3K4me1	-588.2	-613.8	-602.6	442.3	119.9	578.7	263.2	100000	Cistrome	70068
H3K4me1	-442.6	-463.1	-448.4	333.8	ND	575.7	186.6	82838	ENCODE	ENCFF963BLP.bed.gz
H3K4me1	-421.0	-435.7	-421.1	417.8	93.7	553.9	232.9	100000	Cistrome	101556
H3K4me1	-458.5	-479.1	-464.4	401.2	52.5	550.3	180.5	97772	Cistrome	74630
H3K4me1	-399.7	-412.6	-399.4	431.8	98.8	553.2	229.9	100000	Cistrome	85458
H3K4me1	-494.2	-504.1	-493.0	470.2	133.6	543.8	214.4	97947	Cistrome	51161
H3K4me1	-597.6	-621.3	-608.2	433.2	81.0	538.4	210.0	87601	Cistrome	82750
H3K4me1	-543.4	-548.6	-531.4	428.5	62.2	571.1	416.8	100000	Roadm. Epi.	E075-H3K4me1.broadPeak.bed.gz
H3K4me1	-464.1	-479.0	-465.9	424.4	105.6	519.2	207.0	100000	Cistrome	82751
H3K4me1	-445.5	-467.6	-448.5	428.7	116.3	514.0	226.9	100000	Cistrome	82748
H3K4me1	-570.0	-600.9	-591.1	430.1	116.9	525.1	238.6	86089	Cistrome	70076
H3K4me1	-611.9	-639.4	-621.0	353.5	-202.5	516.5	306.0	100000	Roadm. Epi.	E106-H3K4me1.narrowPeak.bed.gz
H3K4me1	-451.4	-468.9	-458.9	414.6	99.4	518.3	230.8	88273	Cistrome	70075
H3K4me1	-449.8	-467.4	-453.4	393.0	91.8	504.4	219.6	91410	Cistrome	70067
H3K4me1	-360.2	-371.4	-360.5	400.7	110.6	496.1	215.9	100000	Cistrome	88015
H3K4me1	-344.7	-358.5	-349.1	403.3	112.5	488.6	226.5	100000	Cistrome	88811
H3K4me1	-465.9	-485.4	-474.6	383.7	66.7	494.5	196.1	78930	Cistrome	9246
H3K4me1	-432.9	-448.9	-433.6	321.2	0.0	485.1	337.1	100000	Roadm. Epi.	E106-H3K4me1.broadPeak.bed.gz
H3K4me1	-409.1	-418.8	-415.9	389.0	103.8	476.7	198.3	86738	Cistrome	84634
H3K4me1	-334.8	-367.7	-356.6	270.2	-0.9	482.3	257.1	79205	Cistrome	57095
H3K4me1	-557.7	-576.3	-565.6	454.8	123.9	474.9	194.3	48818	Cistrome	51145
H3K4me1	-917.8	-919.0	-902.7	467.5	63.2	442.3	145.3	37547	Cistrome	42909
H3K4me1	-509.3	-516.7	-516.5	365.3	64.8	435.0	194.6	41288	Cistrome	101557
H3K4me1	-673.3	-714.7	-691.2	431.7	164.2	422.8	199.3	29827	Cistrome	42908
H3K4me1	-351.9	-335.7	-334.3	395.7	20.0	418.6	74.0	4238	Cistrome	72104
H3K4me1	-407.6	-423.6	-398.3	268.8	6.5	391.8	256.3	100000	Roadm. Epi.	E076-H3K4me1.narrowPeak.bed.gz
H3K4me1	-464.4	-526.0	-466.0	398.3	26.3	406.6	90.0	4839	Cistrome	72106
H3K4me1	-469.0	-485.7	-486.5	370.5	112.4	402.0	172.6	47917	Cistrome	88979
H3K4me1	-622.3	-647.3	-643.1	403.7	1.7	380.1	76.2	35174	Cistrome	86455
H3K4me1	-264.0	-271.0	-255.9	270.3	65.7	375.7	276.4	100000	Roadm. Epi.	E076-H3K4me1.broadPeak.bed.gz
H3K4me1	-282.1	-291.7	-287.3	265.9	12.0	350.0	174.3	34853	Cistrome	101558
H3K4me1	-31.1	-45.8	-36.4	87.4	3.5	86.6	11.1	567	Cistrome	84587
H3K4me1	-63.1	-55.1	-53.3	16.5	-0.3	54.3	2.3	422	Cistrome	45270
H3K4me2	-449.7	-459.2	-447.7	403.0	59.0	536.0	229.9	69442	ENCODE	ENCFF915XUY.bed.gz
H3K4me2	-417.2	-426.3	-414.3	389.9	78.8	464.5	192.3	79557	Cistrome	101374
H3K4me2	-784.9	-809.3	-813.7	396.2	0.2	459.7	135.5	45297	Cistrome	82749
H3K4me2	-478.0	-484.2	-481.4	380.5	39.4	423.2	139.1	62083	Cistrome	101375
H3K4me2	-913.1	-928.0	-906.4	427.5	0.2	417.0	73.6	40745	Cistrome	42911
H3K4me2	-461.0	-462.9	-452.1	400.8	31.5	411.7	113.3	64397	Cistrome	42910
H3K4me2	-530.7	-539.2	-534.8	364.8	1.2	408.0	104.6	52922	Cistrome	70073
H3K4me2	-462.1	-469.6	-456.4	370.4	89.9	404.8	143.1	69348	Cistrome	83378
H3K4me2	-792.5	-820.5	-810.3	345.9	-0.6	401.4	47.7	39615	Cistrome	70066
H3K4me2	-640.8	-652.7	-653.6	361.4	0.0	396.1	35.9	47492	Cistrome	83353
H3K4me2	-638.5	-667.8	-645.6	367.7	0.0	393.7	35.4	42419	Cistrome	70074
H3K4me2	-634.2	-650.1	-647.1	338.4	-0.2	387.1	41.1	40335	Cistrome	70065

H3K4me2	-866.8	-880.0	-845.2	395.5	-0.2	375.0	11.5	23301	Cistrome	83307
H3K4me2	-955.8	-968.7	-952.1	384.0	-3.6	368.5	1.7	29310	Cistrome	70079
H3K4me2	-897.0	-929.1	-926.1	405.9	0.0	365.7	7.5	17134	Cistrome	88737
H3K4me2	-1277.3	-1242.0	-1202.8	355.7	-41.5	357.1	0.0	21206	Cistrome	70081
H3K4me2	-1708.3	-1711.7	-1707.6	452.3	-0.1	329.8	-1.9	7869	Cistrome	83376
H3K4me3	-762.4	-792.4	-772.3	481.1	111.7	509.2	212.2	84678	Roadm. Epi.	E075-H3K4me3.narrowPeak.bed.gz
H3K4me3	-607.3	-628.3	-618.9	384.9	159.6	437.0	96.8	31924	ENCODE	ENCFF023MGT.bed.gz
H3K4me3	-861.7	-900.1	-870.1	406.5	335.5	422.7	15.7	26743	ENCODE	ENCFF575AUU.bed.gz
H3K4me3	-454.8	-463.1	-452.9	368.7	98.7	443.1	275.1	100000	Roadm. Epi.	E075-H3K4me3.broadPeak.bed.gz
H3K4me3	-1000.1	-1107.1	-1034.0	392.5	ND	403.8	17.1	17658	Ensemble	homo_sapiens.GRCh38.HCT116.H3K4me3.SWEmbl_R0005.peaks.20190329.bed.gz
H3K4me3	-929.4	-946.7	-898.2	396.7	-1.5	384.1	10.8	26517	Cistrome	51144
H3K4me3	-793.3	-813.5	-794.2	397.4	-0.8	379.9	2.7	34446	Cistrome	45271
H3K4me3	-877.5	-892.1	-887.4	391.2	3.3	372.2	2.2	31205	Cistrome	92369
H3K4me3	-476.9	-485.1	-476.9	365.1	5.2	373.8	62.2	48602	Cistrome	85292
H3K4me3	-663.8	-686.3	-672.7	378.1	0.2	377.2	18.0	33338	Cistrome	102153
H3K4me3	-854.8	-831.0	-816.8	381.7	-4.3	369.9	1.8	29334	Cistrome	42912
H3K4me3	-590.4	-600.5	-596.3	361.0	0.0	374.5	28.0	31737	Cistrome	102154
H3K4me3	-890.3	-906.2	-871.3	397.2	-4.8	366.7	0.8	30486	Cistrome	42161
H3K4me3	-951.8	-974.8	-943.6	390.3	-5.8	368.9	0.7	28373	Cistrome	89284
H3K4me3	-876.5	-903.5	-866.9	387.6	-1.5	367.9	2.3	29898	Cistrome	92368
H3K4me3	-976.1	-997.0	-973.7	386.6	-5.8	368.1	0.8	27614	Cistrome	89285
H3K4me3	-1105.8	-1139.3	-1089.0	386.2	-8.6	367.7	0.7	25156	Cistrome	42913
H3K4me3	-841.8	-868.2	-848.7	391.8	-1.1	371.5	3.0	26578	Cistrome	81264
H3K4me3	-966.0	-1123.7	-1388.2	246.6	-17.3	370.4	7.4	2983	Cistrome	81266
H3K4me3	-840.6	-854.4	-827.1	378.9	-3.2	366.2	3.4	24080	Cistrome	88713
H3K4me3	-1124.9	-1201.8	-1134.9	376.7	-10.9	362.6	2.2	19739	Cistrome	88725
H3K4me3	-1027.5	-1086.1	-1039.8	378.2	-8.5	358.3	2.6	20453	Cistrome	88698
H3K4me3	-987.5	-1024.7	-989.7	388.2	-10.2	352.6	0.1	27726	Cistrome	42160
H3K4me3	-1004.3	-1066.4	-1018.9	397.1	-5.0	353.8	0.2	27346	Cistrome	81633
H3K4me3	-611.6	-640.5	-656.2	385.6	2.9	368.8	50.7	39820	Cistrome	51160
H3K4me3	-998.4	-1042.3	-1009.5	396.8	-7.0	351.1	0.2	26055	Cistrome	56017
H3K4me3	-1169.5	-1234.0	-1236.0	391.8	-6.4	353.4	0.1	22478	Cistrome	57094
H3K4me3	-1059.0	-1076.0	-1081.2	391.5	-4.5	353.9	0.1	25236	Cistrome	81265
H3K4me3	-1014.3	-1060.9	-1033.0	415.8	0.8	360.4	15.8	27993	Cistrome	42162
H3K4me3	-959.6	-1009.1	-958.0	388.8	-7.8	350.7	0.4	25988	Cistrome	56016
H3K4me3	-768.8	-779.9	-771.1	374.0	-3.3	352.9	3.5	22224	Cistrome	88697
H3K4me3	-1188.4	-1247.6	-1184.9	334.9	-43.4	350.5	0.4	16524	Cistrome	70064
H3K4me3	-1053.6	-1081.8	-1083.2	392.2	-7.3	343.0	0.0	24912	Cistrome	81625
H3K4me3	-876.9	-915.9	-893.6	398.2	-1.1	345.6	0.3	25654	Cistrome	56019
H3K4me3	-1073.8	-1174.4	-1082.7	357.5	-18.9	347.5	0.5	18426	Cistrome	70071
H3K4me3	-1174.0	-1239.4	-1157.5	334.2	-40.8	341.3	0.2	16204	Cistrome	70063
H3K4me3	-1078.5	-1140.5	-1079.7	366.0	-18.6	344.8	0.2	17739	Cistrome	85447
H3K4me3	-1054.1	-1148.6	-1046.3	376.7	-15.6	344.9	0.3	19186	Cistrome	85451
H3K4me3	-907.4	-889.7	-859.0	327.6	-80.5	336.0	1.9	19549	Cistrome	38319
H3K4me3	-907.4	-889.7	-859.0	327.6	-80.5	336.0	1.9	19549	Cistrome	8440
H3K4me3	-1176.4	-1234.7	-1155.6	363.8	-21.5	338.6	0.3	19113	Cistrome	81635
H3K4me3	-1143.4	-1180.0	-1129.9	355.2	-19.3	342.4	0.2	19410	Cistrome	70072
H3K4me3	-1176.5	-1219.3	-1136.7	357.4	-26.2	341.9	0.2	16561	Cistrome	85449
H3K4me3	-530.8	-546.0	-530.1	359.6	138.8	356.5	168.1	72243	Roadm. Epi.	E076-H3K4me3.narrowPeak.bed.gz
H3K4me3	-1234.7	-1254.3	-1188.5	340.7	-56.7	333.2	0.0	18978	Cistrome	72103
H3K4me3	-1248.3	-1268.7	-1274.5	346.3	-38.3	336.6	0.1	15522	Cistrome	85446
H3K4me3	-1196.1	-1226.9	-1185.6	350.6	-31.0	334.5	0.0	16408	Cistrome	85452
H3K4me3	-1137.1	-1223.9	-1128.2	361.2	-24.3	334.6	0.0	17932	Cistrome	85453
H3K4me3	-1053.0	-1067.9	-1035.8	335.9	-55.9	329.0	0.0	15627	Cistrome	72105
H3K4me3	-1229.8	-1222.5	-1201.3	342.0	-39.5	333.5	0.1	15943	Cistrome	85448
H3K4me3	-1249.0	-1267.8	-1236.7	354.5	-29.1	332.3	0.0	16383	Cistrome	85450
H3K4me3	-975.9	-1000.3	-946.7	379.7	-9.0	324.4	0.0	23519	Cistrome	56018
H3K4me3	-863.5	-860.9	-853.8	346.1	-12.6	322.3	2.1	21480	Cistrome	38321
H3K4me3	-660.4	-688.0	-693.7	317.2	-28.8	317.4	2.5	17967	Cistrome	49532
H3K4me3	-409.0	-425.8	-411.3	245.4	172.5	280.3	114.7	68861	Roadm. Epi.	E106-H3K4me3.narrowPeak.bed.gz
H3K4me3	-271.5	-277.3	-267.1	237.3	130.6	285.9	201.1	100000	Roadm. Epi.	E076-H3K4me3.broadPeak.bed.gz
H3K4me3	-147.2	-149.2	-142.5	132.5	1.1	223.6	151.2	100000	Roadm. Epi.	E106-H3K4me3.broadPeak.bed.gz
H3K79me2	-852.1	-903.2	-890.3	259.1	-155.6	440.9	137.8	10541	Cistrome	101708
H3K79me2	-630.1	-700.2	-644.3	225.8	-144.0	418.7	214.8	8655	Cistrome	101707
H3K79me2	-46.9	-45.9	-45.3	25.2	-19.3	245.6	6.6	2368	Cistrome	83603
H3K79me2	0.0	0.0	-0.1	160.0	1.9	195.0	40.0	20855	Cistrome	88455
H3K9ac	-781.0	-791.5	-785.6	539.8	330.6	674.8	282.0	100000	Roadm. Epi.	E075-H3K9ac.narrowPeak.bed.gz
H3K9ac	-416.4	-424.9	-422.6	372.4	270.2	540.1	360.8	100000	Roadm. Epi.	E075-H3K9ac.broadPeak.bed.gz
H3K9ac	-596.9	-613.3	-604.6	349.0	157.9	510.0	125.0	36097	ENCODE	ENCFF724JXS.bed.gz
H3K9ac	-546.1	-544.9	-553.2	367.3	-0.6	417.9	25.9	49287	Cistrome	87280
H3K9ac	-501.4	-516.8	-492.6	349.9	363.8	426.2	227.4	60918	Roadm. Epi.	E076-H3K9ac.narrowPeak.bed.gz
H3K9ac	-722.5	-733.0	-726.1	353.8	-11.2	408.8	14.9	32466	Cistrome	100642
H3K9ac	-566.9	-567.6	-580.4	362.4	-1.7	393.6	12.7	44150	Cistrome	87279
H3K9ac	-687.9	-687.5	-697.4	334.2	-22.4	392.5	4.2	29925	Cistrome	100641
H3K9ac	-515.3	-520.4	-522.5	356.6	0.0	391.3	52.2	40603	Cistrome	87278
H3K9ac	-568.7	-581.4	-577.8	369.2	0.2	376.7	42.0	40039	Cistrome	87273
H3K9ac	-510.3	-511.1	-523.4	328.9	0.1	337.4	41.2	24383	Cistrome	82746
H3K9ac	-535.5	-529.2	-539.2	313.0	0.0	335.6	37.2	18541	Cistrome	87272
H3K9ac	-156.2	-152.2	-148.1	199.1	219.4	354.7	285.3	100000	Roadm. Epi.	E076-H3K9ac.broadPeak.bed.gz
H3K9ac	-621.7	-643.6	-623.8	315.6	-0.5	318.0	13.2	20226	Cistrome	82747
H3K9ac	-835.8	-857.0	-847.6	337.7	-0.8	298.0	3.0	13135	Cistrome	82745

H3K9ac	-649.1	-677.5	-657.7	278.0	-1.3	284.8	5.1	5854	Cistrome	82744
H3K9me2	-646.1	-707.2	-690.4	522.2	273.2	332.6	211.9	16338	Cistrome	85108
H3K9me2	-0.6	-0.3	-0.2	0.6	7.5	0.0	0.0	1493	Cistrome	54504
H3K9me2	290.7	334.9	323.0	-1.0	-10.9	-3.7	0.0	4735	ENCODE	ENCFF045ELD.bed.gz
H3K9me2	-107.3	-96.9	-66.4	-97.4	-46.4	-132.3	-142.8	612	Cistrome	93700
H3K9me2	-109.6	-84.8	-68.2	-108.3	-50.6	-200.0	-215.5	726	Cistrome	93701
H3K9me3	-11.0	-14.9	-7.6	-43.1	-27.2	-25.0	-38.8	471	Cistrome	51148
H3K9me3	-32.1	-26.8	-25.2	-57.7	-34.5	-55.6	-119.5	715	Cistrome	51163
H3K9me3	-17.1	-14.8	-15.1	4.5	-10.4	-45.7	-63.0	100000	Roadm. Epi.	E075-H3K9me3.broadPeak.bed.gz
H3K9me3	2.2	8.5	1.9	-0.3	-93.2	-103.1	-83.7	100000	Roadm. Epi.	E076-H3K9me3.broadPeak.bed.gz
H3K9me3	-50.2	-20.6	-42.4	-17.9	-113.2	-122.4	-56.0	64003	Roadm. Epi.	E076-H3K9me3.narrowPeak.bed.gz
H3K9me3	-169.0	-180.4	-180.1	-4.8	-81.8	-185.6	-162.8	100000	Roadm. Epi.	E075-H3K9me3.narrowPeak.bed.gz
H3K9me3	85.2	95.0	87.0	-146.1	-63.4	-248.3	-204.0	100000	Roadm. Epi.	E106-H3K9me3.broadPeak.bed.gz
H3K9me3	-31.2	-38.6	-44.6	-233.9	0.0	-329.2	-245.5	100000	Roadm. Epi.	E106-H3K9me3.narrowPeak.bed.gz
H3K9me3	198.1	208.8	199.8	-407.1	ND	-420.4	-383.1	116955	Ensemble	homo_sapiens.GRCh38.HCT116.H3K9me3.ccat_histone.peaks.20190329.mergedd1000.bed.gz
H3K9me3	312.5	327.6	329.7	-664.1	121.0	-645.0	-611.4	100000	ENCODE	ENCFF580MMU.bed.gz
H4K16ac	-595.3	-607.4	-624.7	457.0	48.4	503.2	390.2	2290	Cistrome	83887
H4K16ac	-842.0	-847.7	-899.3	383.7	0.4	382.0	62.8	25148	Cistrome	83526
H4K20me1	-27.0	-34.5	-37.7	0.0	-1933.6	481.4	425.1	20508	ENCODE	ENCFF937WLG.bed.gz
H4K20me3	-219.9	-227.2	-223.6	237.6	6.3	222.4	66.1	39942	Cistrome	88457
**polymerase II - active transcription**										
POLR2A	-809.5	-684.6	-670.6	143.4	-53.5	568.1	155.3	3029	Cistrome	82412
POLR2A	-515.1	-535.9	-534.7	276.6	-27.5	507.2	306.1	66413	Cistrome	34398
POLR2A	-760.5	-775.3	-763.7	389.4	-1.7	463.8	147.1	31718	Cistrome	82333
POLR2A	-542.0	-549.1	-543.1	392.0	14.6	452.9	166.0	55528	Cistrome	46201
POLR2A	-951.7	-857.4	-767.0	94.8	-123.0	469.0	60.5	4543	Cistrome	55679
POLR2A	-452.0	-388.3	-421.3	34.8	-65.2	461.3	12.5	1700	Cistrome	86621
POLR2A	-924.7	-929.8	-919.8	366.9	-21.4	443.1	74.8	24151	Cistrome	92091
POLR2A	-652.8	-656.2	-652.2	400.9	1.3	438.1	121.8	40923	Cistrome	39917
POLR2A	-819.1	-807.4	-804.5	351.8	-27.9	439.6	118.3	23335	Cistrome	88903
POLR2A	-644.3	-649.4	-652.1	395.9	1.1	438.9	121.9	41125	Cistrome	39921
POLR2A	-963.2	-962.3	-955.4	360.7	-28.4	436.8	50.4	23301	Cistrome	89219
POLR2A	-940.5	-950.6	-954.2	399.7	-4.1	434.6	49.2	24689	Cistrome	93206
POLR2A	-1014.6	-1051.9	-1017.0	362.7	-32.0	429.4	27.6	20360	Cistrome	89218
POLR2A	-903.8	-909.0	-904.4	350.1	-40.8	429.6	101.3	20196	Cistrome	83958
POLR2A	-841.8	-849.6	-844.5	380.7	-7.4	419.2	25.1	25213	Cistrome	88093
POLR2A	-874.5	-864.5	-859.4	338.6	-57.3	420.7	70.2	19821	Cistrome	83956
POLR2A	-857.3	-845.8	-844.8	348.6	-31.3	415.3	36.4	20421	Cistrome	83953
POLR2A	-919.5	-918.3	-918.0	380.1	-9.1	414.1	18.4	21544	Cistrome	93205
POLR2A	-843.9	-922.3	-921.9	429.4	15.8	417.8	160.2	45426	Cistrome	57088
POLR2A	-601.9	-573.6	-402.9	26.7	-156.7	417.0	9.2	2749	Cistrome	55677
POLR2A	-936.3	-945.2	-938.1	330.0	-45.3	409.7	30.6	17288	Cistrome	83950
POLR2A	-949.2	-1010.8	-1020.3	395.7	-0.5	402.0	20.0	27176	Cistrome	5661
POLR2A	-793.7	-794.4	-781.8	350.6	-15.7	404.8	14.6	21269	Cistrome	83959
POLR2A	-742.6	-731.6	-732.2	341.6	-9.0	404.3	20.6	23176	Cistrome	88100
POLR2A	-743.5	-732.8	-735.3	352.1	-7.5	401.4	12.9	24764	Cistrome	82337
POLR2A	-724.8	-701.9	-708.1	341.4	-5.9	399.5	27.9	23854	Cistrome	88099
POLR2A	-911.1	-945.4	-953.4	373.6	-7.9	394.0	13.8	20284	Cistrome	83342
POLR2A	-1097.7	-1135.9	-1100.4	341.8	151.5	389.1	1.5	16764	ENCODE	ENCFF271RGE.bed.gz
POLR2A	-865.7	-869.2	-879.6	345.1	-32.3	391.7	9.8	18859	Cistrome	83957
POLR2A	-1180.4	-1255.8	-1171.2	339.8	-20.7	387.4	1.5	15512	ENCODE	ENCFF786BQV.bed.gz
POLR2A	-710.6	-701.0	-697.4	340.5	-5.1	396.0	24.2	22757	Cistrome	83951
POLR2A	-1014.6	-1007.6	-1005.5	371.0	-14.9	387.3	9.4	18540	Cistrome	88094
POLR2A	-835.7	-820.7	-838.2	364.4	-5.5	387.7	6.6	21013	Cistrome	88096
POLR2A	-670.8	-647.1	-665.2	329.2	-6.6	385.3	9.4	22512	Cistrome	88091
POLR2A	-608.6	-617.7	-617.4	352.9	-1.0	389.1	38.9	22283	Cistrome	86928
POLR2A	-999.5	-1042.7	-1001.5	364.9	-15.0	374.5	5.0	17656	Cistrome	45715
POLR2A	-853.3	-851.4	-841.3	375.3	-2.3	372.0	1.8	27623	Cistrome	68779
POLR2A	-736.6	-719.4	-728.9	313.2	-27.5	379.1	5.2	18860	Cistrome	83955
POLR2A	-909.4	-892.4	-917.5	378.8	-2.7	375.7	0.8	19702	Cistrome	49535
POLR2A	-654.6	-663.4	-662.4	370.6	0.9	375.4	16.8	30595	Cistrome	88095
POLR2A	-1188.1	-1177.4	-1225.7	338.6	-43.6	371.9	3.1	9895	Cistrome	88098
POLR2A	-758.9	-742.9	-747.1	362.6	-1.5	368.4	1.5	26413	Cistrome	68785
POLR2A	-583.6	-584.6	-598.5	391.0	1.7	377.5	36.2	25215	Cistrome	86927
POLR2A	-768.8	-742.1	-754.4	336.8	-14.7	373.1	1.5	19072	Cistrome	84236
POLR2A	-967.8	-1107.6	-1049.3	275.6	-24.3	371.3	9.9	5224	Cistrome	82395
POLR2A	-1539.8	-1491.4	-1684.9	341.1	-55.4	356.7	0.4	7560	Cistrome	92088
POLR2A	-837.5	-882.5	-848.4	347.9	-12.8	365.0	5.3	21087	Cistrome	55671
POLR2A	-765.7	-777.9	-776.0	354.3	-0.3	367.4	14.8	22292	Cistrome	82336
POLR2A	-891.2	-904.0	-896.4	383.9	-0.2	360.5	2.1	26872	Cistrome	1152
POLR2A	-996.3	-1034.1	-1038.7	349.9	-10.9	363.9	6.4	15181	Cistrome	83344
POLR2A	-957.7	-979.2	-1002.3	358.0	-12.9	359.2	0.1	17619	Cistrome	83343
POLR2A	-907.3	-869.2	-852.6	357.8	-4.4	357.3	0.3	23166	Cistrome	68784
POLR2A	-1013.0	-1026.5	-987.0	338.0	-18.4	362.3	1.5	11472	Cistrome	84237
POLR2A	-1165.9	-1226.7	-1200.2	356.5	-20.6	355.0	0.6	14701	Cistrome	55662
POLR2A	-881.5	-862.9	-887.3	369.8	-5.5	355.3	2.5	19125	Cistrome	57089
POLR2A	-1232.7	-1249.3	-1242.7	352.9	-26.7	351.3	0.1	14697	Cistrome	55672
POLR2A	-1252.6	-1323.7	-1270.2	323.8	-34.7	345.3	1.5	9002	Cistrome	55663
POLR2A	-1012.4	-1065.7	-1091.5	351.1	-13.3	356.1	2.4	14058	Cistrome	83349
POLR2A	-706.6	-774.6	-826.3	119.4	-20.3	353.7	4.8	2841	Cistrome	82396
POLR2A	-560.4	-577.3	-585.2	386.9	2.5	361.4	35.4	24672	Cistrome	86929

POLR2A	-890.8	-919.3	-897.0	343.6	-11.5	345.9	0.3	19957	Cistrome	68778
POLR2A	-1254.4	-1243.7	-1222.6	314.6	-57.9	340.0	0.2	10710	Cistrome	55673
POLR2A	-670.3	-685.3	-679.0	347.7	0.0	349.6	6.8	24837	Cistrome	84239
POLR2A	-1085.5	-1124.3	-1076.7	331.0	-37.0	343.1	0.4	14541	Cistrome	55675
POLR2A	-1020.0	-1018.4	-1004.7	303.2	-56.7	339.3	0.5	10930	Cistrome	55674
POLR2A	-1108.2	-1112.5	-1170.2	276.7	-23.4	342.3	15.0	5158	Cistrome	82394
POLR2A	-1113.9	-1170.5	-1228.2	271.3	-34.9	341.8	8.6	6486	Cistrome	83350
POLR2A	-1118.6	-1173.1	-1210.7	314.8	-16.0	323.0	1.7	9147	Cistrome	49533
POLR2A	-1182.7	-1215.6	-1241.3	323.8	-17.3	311.9	0.4	9599	Cistrome	49534
POLR2A	-735.2	-742.2	-742.7	346.2	3.5	321.7	94.8	21629	Cistrome	89206
POLR2A	-740.6	-780.3	-773.5	307.3	-7.0	303.9	0.1	21388	Cistrome	1151
POLR2A	-149.5	-115.9	-112.8	9.6	-47.4	275.3	13.2	587	Cistrome	86619
POLR2A	-242.7	-267.5	-240.6	39.3	-16.9	189.0	6.4	1241	Cistrome	86354
POLR2A	-133.5	-135.0	-132.1	34.6	-7.5	127.5	2.0	429	Cistrome	82332
POLR2A	-324.0	-331.7	-321.6	51.9	-17.4	105.3	0.1	1387	Cistrome	55670
POLR2A	-117.4	-131.7	-181.0	27.2	-8.1	79.9	4.0	746	Cistrome	92642
POLR2A	-256.4	-209.0	-254.7	48.4	-18.7	75.1	0.0	705	Cistrome	88092
POLR2A	-143.3	-129.3	-126.1	33.0	-2.8	83.9	2.0	681	Cistrome	92641
POLR2A	-257.3	-211.4	-187.7	0.4	-108.9	90.6	0.1	967	Cistrome	55678
POLR2A	-148.5	-150.0	-147.0	32.1	-2.4	42.5	0.0	455	Cistrome	76192
POLR2A	-109.1	-91.9	-109.0	14.7	-4.7	16.2	-0.9	485	Cistrome	76190
POLR2A	-130.0	-132.6	-70.1	-0.2	-78.6	11.8	-0.7	525	Cistrome	55680
POLR2A-5Sp	-720.0	-699.2	-703.5	347.4	44.1	478.3	122.5	40045	ENCODE	ENCFF910KOG.bed.gz
POLR2A-5Sp	-759.6	-741.0	-750.8	345.8	ND	474.6	92.1	36786	ENCODE	ENCFF934KIL.bed.gz
**DNA accessibility**										
ATAC-seq	-462.3	-474.3	-463.6	398.5	134.5	384.5	161.3	61825	Cistrome	105339
ATAC-seq	-486.0	-488.5	-472.4	397.5	107.2	381.0	159.1	47323	Cistrome	105341
ATAC-seq	-441.0	-443.5	-436.9	384.2	110.0	368.7	149.8	51038	Cistrome	105340
ATAC-seq	-488.2	-493.3	-481.9	377.2	112.4	358.0	138.9	43777	Cistrome	105338
ATAC-seq	-513.5	-488.8	-495.1	305.3	0.0	337.7	11.9	4594	Cistrome	105336
ATAC-seq	-380.7	-386.0	-380.0	331.3	129.8	262.2	33.3	52706	Cistrome	66174
ATAC-seq	-347.1	-351.8	-347.4	284.6	121.0	187.1	0.2	38536	Cistrome	78866
ATAC-seq	-367.6	-376.4	-366.8	288.2	53.7	181.2	0.0	27142	Cistrome	81122
ATAC-seq	-315.4	-321.2	-312.9	273.2	94.2	165.5	-0.4	36453	Cistrome	81121
ATAC-seq	-298.1	-308.0	-300.3	266.2	116.7	151.5	-0.3	40498	Cistrome	78867
ATAC-seq	-355.2	-363.0	-350.8	267.8	129.3	144.5	-1.1	38154	Cistrome	80026
ATAC-seq	-384.5	-394.7	-386.2	275.3	128.9	137.0	-3.4	34053	Cistrome	80027
ATAC-seq	-346.3	-352.9	-345.5	264.0	123.9	135.3	-2.9	34750	Cistrome	80024
ATAC-seq	-151.3	-143.2	-186.2	67.0	0.1	80.9	0.1	1432	Cistrome	105335
ATAC-seq	-110.6	-103.7	-146.3	56.4	0.6	61.4	0.1	1148	Cistrome	105337
ATAC-seq	-90.8	-93.1	-120.3	18.8	0.4	18.6	-0.4	844	Cistrome	105334
ATAC-seq	-485.2	-483.4	-469.8	279.5	15.6	4.5	-15.9	7797	Cistrome	80025
DNase	-353.6	-361.7	-349.3	356.9	104.8	390.2	152.8	80101	Cistrome	44956
DNase	-347.8	-356.7	-343.1	359.0	114.1	384.3	155.7	78833	Cistrome	42166
DNAse	-344.8	-352.8	-339.5	352.4	107.3	384.0	156.3	76277	Cistrome	42165
DNase	-383.0	-388.3	-377.4	356.2	101.9	372.7	139.3	67217	Cistrome	44955
DNase	-395.6	-406.8	-387.9	384.5	113.3	375.1	158.6	59718	Cistrome	42167
DNAse	-441.3	-448.1	-435.9	369.6	130.7	353.1	144.4	56316	Cistrome	42168
DNAseI	-449.9	-452.3	-443.5	339.4	-0.1	376.3	129.9	50504	Ensemble	
**Transcription factors**										
AFF4	-143.7	-147.9	-102.7	14.5	-19.5	65.3	0.0	1126	Cistrome	55639
AFF4	-30.0	-25.0	-29.9	0.0	-9.6	50.1	-0.1	483	Cistrome	39916
AFF4	-147.3	-174.6	-111.4	4.0	-17.5	22.5	-0.5	1331	Cistrome	55641
AFF4	-44.9	-76.3	-69.6	12.3	2.0	5.8	0.4	3112	Cistrome	1140
AFF4	-83.9	-78.3	-57.8	1.5	-14.5	5.8	-6.1	658	Cistrome	55640
AFF4	-29.8	-45.9	-54.3	4.4	0.0	3.4	0.0	969	Cistrome	1141
ARID1A	-588.2	-614.6	-605.0	430.2	56.5	455.0	185.9	46436	Cistrome	88976
ARID1A	-603.8	-616.6	-612.4	404.6	46.4	410.5	176.9	21805	Cistrome	88981
ARID1A	-35.6	-34.5	-30.0	-0.1	0.9	-3.2	-1.4	1872	Cistrome	85741
ARID1A	-46.1	-41.7	-39.6	-23.8	-5.7	-52.1	-68.3	768	Cistrome	85740
ATF3	-490.4	-491.7	-477.9	371.1	72.9	390.7	151.5	49476	Cistrome	46217
ATF3	-513.6	-509.0	-500.9	304.0	ND	366.3	63.2	25474	Ensemble	homo_sapiens.GRCh38.HCT116.ATF3.SWEmbl_R0005.peaks.20190329.bed.gz
ATF3	-332.9	-341.7	-332.1	309.8	108.8	357.3	161.0	60225	Cistrome	68464
ATF3	-357.8	-359.5	-360.7	278.0	20.1	306.7	55.1	25930	Cistrome	68460
ATF3	-190.4	-196.7	-166.7	21.0	-7.1	44.0	0.6	827	Cistrome	68463
BANP	-18.4	-18.9	-23.4	54.2	24.3	3.3	-0.7	4666	Cistrome	71951
BANP	-25.3	-22.9	-39.4	40.7	13.4	1.3	-5.2	6183	Cistrome	71950
BRD4	-613.6	-583.6	-603.9	268.0	-1.4	339.4	0.1	7401	Cistrome	68777
BRD4	-578.6	-561.6	-578.6	296.1	-0.2	331.6	2.1	10531	Cistrome	68783
BRD4	-760.6	-694.2	-719.4	309.1	-5.4	267.3	0.0	3725	Cistrome	67539
BRD4	-387.5	-420.2	-383.8	145.9	-0.6	245.9	0.1	3745	Cistrome	68782
BRD4	-86.2	-79.3	-80.1	8.1	-2.8	61.3	-0.1	975	Cistrome	68776
BRDU	-138.2	-126.6	-81.5	-94.3	-24.1	-124.7	-177.3	734	Cistrome	91681
CBX3	-356.8	-356.0	-352.2	394.0	122.8	445.4	200.4	97142	Cistrome	46209
CBX3	-279.7	-293.0	-285.5	10.1	-51.5	378.7	265.9	16531	Cistrome	9228
CBX3	-290.2	-316.2	-309.2	3.1	-137.4	369.2	286.4	41056	Cistrome	9230
CBX3	-30.3	-34.9	-23.8	78.9	37.3	23.2	0.0	1278	Cistrome	9237
CBX3	-12.9	-7.5	-6.3	2.3	2.2	1.7	0.0	1491	Cistrome	9231
CBX3	0.0	0.0	-0.5	0.3	0.0	0.6	0.0	2594	Cistrome	9232

CBX3	-19.8	-27.4	-19.4	5.0	-27.6	0.6	-2.6	574	Cistrome	9236
CDK9	-850.4	-937.0	-939.0	408.6	25.4	264.1	6.8	12576	Cistrome	57093
CEBPB	-391.6	-396.3	-385.2	359.2	98.2	400.2	174.4	66557	Cistrome	46206
CEBPB	-418.3	-424.2	-410.3	289.8	ND	375.1	125.0	30413	Ensemble	homo_sapiens.GRCh38.HCT116.CEBPB.SWEmbl_R0005.peaks.20190329.bed.gz
CENPA_lift Over	-352.5	-362.7	-351.1	-666.9	-674.6	-657.0	-656.3	1056		CENPA_GSM1105684_2fold_enriched_merged_hg38.bed.gz
CENPA-HuRef*	-432.3	-442.1	-324.0	-889.4	-658.1	-866.3	-755.5	1598	Cistrome	40153
CNOT3	-775.1	-786.6	-812.2	475.4	152.3	320.1	49.7	47784	Cistrome	76964
CTCF	-312.7	-327.0	-306.4	332.9	141.9	330.5	199.2	54768	Cistrome	46218
CTCF	-328.8	-345.7	-323.7	326.9	150.9	312.4	199.9	54059	Cistrome	85285
CTCF	-288.7	-304.2	-283.9	320.6	-290.8	302.2	190.7	49581	ENCODE	ENCFF850PKJ.bed.gz
CTCF	-321.1	-339.5	-316.9	316.6	146.7	300.8	195.5	41280	Cistrome	85284
CTCF	-250.6	-263.6	-242.5	307.5	3.7	300.2	196.0	58981	ENCODE	ENCFF518MQA.bed.gz
CTCF	-286.0	-297.8	-274.8	324.1	144.6	303.6	195.7	36079	Cistrome	42151
CTCF	-278.8	-292.7	-272.4	319.9	56.3	300.5	188.2	52155	ENCODE	ENCFF171SNH.bed.gz
CTCF	-293.2	-308.2	-288.4	316.9	143.7	300.2	201.8	29057	Cistrome	72779
CTCF	-330.7	-332.7	-326.3	314.9	143.4	300.0	189.7	23805	Cistrome	85286
CTCF	-249.6	-259.3	-238.0	314.7	154.2	292.3	185.6	51750	Cistrome	42152
CTCF	-294.1	-308.1	-285.7	307.9	153.5	294.1	193.9	42363	Cistrome	45717
CTCF	-294.1	-307.8	-285.4	307.7	153.3	294.0	193.9	42385	Cistrome	42148
CTCF	-255.2	-273.0	-247.5	323.5	-54.7	293.0	232.1	30116	ENCODE	ENCFF056ESE.bed.gz
CTCF	-269.1	-284.5	-264.4	303.6	152.5	285.8	183.6	52042	Cistrome	42149
CTCF	-268.9	-284.1	-264.2	303.5	152.4	285.7	183.4	52021	Cistrome	45716
CTCF	-273.9	-287.0	-268.2	316.6	156.9	283.8	180.6	45381	Cistrome	42154
CTCF	-242.8	-255.4	-235.7	306.0	149.5	283.0	186.0	45369	Cistrome	42150
CTCF	-239.7	-250.6	-235.0	315.9	-59.0	284.4	227.3	35571	ENCODE	ENCFF549PGC.bed.gz
CTCF	-269.5	-285.7	-264.8	317.5	155.7	283.0	176.6	49084	Cistrome	42153
CTCF	-274.6	-291.0	-268.5	277.8	ND	264.4	176.5	50595	Enseble	homo_sapiens.GRCh38.HCT116.CTCF.SWEmbl_R0005.peaks.20190329.bed.gz
EGR1	-584.6	-587.7	-578.0	403.0	46.8	409.9	151.1	47145	Cistrome	46214
Egr1	-800.6	-780.5	-799.7	341.8	0.0	393.0	6.1	17084	Ensemble	homo_sapiens.GRCh38.HCT116.Egr1.SWEmbl_R0005.peaks.20190329.bed.gz
ELF1	-554.5	-563.6	-554.3	393.0	66.9	400.8	146.2	45158	Cistrome	46202
ELF1	-679.7	-685.1	-669.2	323.6	ND	385.4	33.6	24133	Ensemble	homo_sapiens.GRCh38.HCT116.ELF1.SWEmbl_R0005.peaks.20190329.bed.gz
ELL2	-599.4	-606.4	-546.7	230.5	-3.7	356.5	7.5	4198	Cistrome	55645
ELL2	-261.2	-250.9	-241.6	61.6	-2.9	139.3	0.3	1977	Cistrome	55647
ELL2	-157.2	-170.0	-163.5	46.3	-0.8	89.6	0.5	1387	Cistrome	55646
ELL2	-152.7	-191.3	-151.1	39.3	-0.6	69.0	0.5	1098	Cistrome	55648
ELL2	-133.0	-153.0	-131.8	28.9	0.1	26.5	0.3	1683	Cistrome	1143
ELL2	-30.5	-23.5	-31.9	1.1	-0.1	8.2	0.1	1036	Cistrome	1144
EP300	-259.9	-250.9	-257.9	250.5	28.5	273.8	17.6	19798	Cistrome	42907
EZH2	-69.4	-60.1	-42.9	0.1	2.7	-1.8	-14.4	672	Cistrome	101554
EZH2	-25.1	-22.1	-19.4	2.8	-33.3	-5.1	-7.0	660	ENCODE	ENCFF806ESQ.bed.gz
EZH2	-18.9	-9.4	-9.9	0.9	ND	-7.1	-7.5	867	ENCODE	ENCFF926EZW.bed.gz
EZH2	-94.9	-87.3	-37.9	-21.5	-8.4	-53.6	-63.9	616	Cistrome	101555
EZH2	-265.8	-315.2	-231.5	-11.0	ND	-240.6	-163.6	5222	Ensemble	homo_sapiens.GRCh38.HCT116.EZH2.SWEmbl_R0005.peaks.20190329.bed.gz
EZH2-T487p	-71.9	-84.8	-40.9	-36.8	-13.8	-91.4	-106.0	560	Cistrome	100246
EZH2-T487p	-4.6	-4.1	-4.4	7.5	125.9	-0.5	-0.1	425	ENCODE	ENCFF325ZGB.bed.gz
FOSL1	-388.8	-397.4	-386.8	366.3	109.4	401.9	174.0	70141	Cistrome	46212
FOSL1	-376.0	-376.1	-383.3	279.6	-615.0	352.2	87.0	31315	Ensemble	homo_sapiens.GRCh38.HCT116.FOSL1.SWEmbl_R0005.peaks.20190329.bed.gz
HEXIM1	-593.4	-631.4	-620.8	460.5	161.7	325.7	138.0	55397	Cistrome	57092
HNF4A	-214.7	-193.7	-197.2	227.0	2.1	348.6	35.7	3800	Cistrome	54666
HNF4A	-390.9	-391.6	-376.9	275.2	45.5	340.9	184.7	25783	Cistrome	54673
HNF4A	-311.9	-269.2	-246.3	294.5	2.2	336.6	29.7	4471	Cistrome	54665
HNF4A	-396.1	-416.5	-388.0	272.3	29.7	331.0	177.8	15595	Cistrome	54674
HNF4A	-132.9	-109.2	-119.1	137.3	1.3	241.2	20.2	2286	Cistrome	54660
HSF1	-115.8	-119.3	-91.6	2.6	-1.0	9.0	0.0	851	Cistrome	51108
HSF1	-128.9	-109.9	-82.8	2.6	-1.1	6.9	-0.4	772	Cistrome	51110
HSF1	-80.6	-117.7	-69.3	-0.4	-9.8	0.1	-2.9	486	Cistrome	51107
ICE1	-473.8	-469.4	-464.1	356.2	103.1	365.1	159.2	41898	Cistrome	39920
ICE1	-443.4	-451.1	-444.4	349.3	91.0	356.7	155.0	39819	Cistrome	39914
ICE1	-59.9	-49.5	-66.0	10.1	-1.2	50.1	1.2	548	Cistrome	39919
ICE2	-123.2	-92.9	-98.5	173.9	5.6	204.1	18.9	2316	Cistrome	39924
JUND	-420.8	-427.6	-416.1	377.0	108.6	412.3	172.0	69908	Cistrome	46213
Jund	-440.3	-437.6	-442.5	291.0	ND	375.5	79.2	31993	Ensemble	homo_sapiens.GRCh38.HCT116.Jund.SWEmbl_R0005.peaks.20190329.bed.gz
JUND	-260.8	-254.5	-255.1	222.5	ND	275.0	74.1	23965	ENCODE	ENCFF998KDQ.bed.gz
JUND	-261.3	-261.6	-268.8	221.2	-2.4	269.5	54.9	21907	ENCODE	ENCFF333VCK.bed.gz
KAT2B	-153.6	-109.7	-117.6	-21.5	-12.1	-57.4	-88.8	728	Cistrome	85025
KDM3B	-412.9	-401.7	-395.0	313.6	57.5	310.7	173.9	15346	Cistrome	82127
KDM3B	-289.4	-262.0	-240.6	337.5	32.6	312.9	125.6	5043	Cistrome	82126
KDM5A	-1055.0	-1298.9	-1138.1	146.8	-26.2	363.5	32.8	4675	Cistrome	92366
KDM5A	-1036.4	-1241.3	-1118.4	173.6	-2.1	358.0	28.4	5429	Cistrome	92367
KMT2B	-260.0	-255.2	-259.1	262.6	25.1	299.3	27.7	21353	Cistrome	42906
LARP7	-530.1	-568.0	-571.3	422.3	181.8	255.8	122.0	39326	Cistrome	57091
LEO1	-331.4	-244.1	-261.0	16.0	-19.1	242.7	13.1	1500	Cistrome	55661

MAX	-504.0	-512.3	-499.0	417.9	118.8	446.1	176.8	74266	Cistrome	46216
Max	-719.8	-735.1	-720.0	380.4	ND	438.6	118.9	33616	Ensemble	homo_sapiens.GRCh38.HCT116.Max.SWEmbl_R0005.peaks.20190329.bed.gz
MCM2	-322.6	-334.4	-326.6	242.2	234.7	148.3	137.4	65110	Cistrome	83886
MECP2	-458.7	-465.9	-462.9	316.3	39.8	322.7	163.8	34007	Cistrome	34399
MECP2	-310.3	-342.2	-261.8	255.3	20.4	239.1	51.5	8828	Cistrome	34400
MTA2	-59.9	-47.3	-45.2	-37.4	-21.8	-77.1	-77.1	474	Cistrome	87604
MYC	-1065.3	-1051.0	-1083.3	358.0	-7.6	361.9	1.0	13446	Cistrome	70809
MYC	-1008.2	-1035.8	-1016.0	371.7	-1.9	343.1	2.8	15844	Cistrome	70807
MYC	-888.2	-879.5	-893.3	404.6	0.0	328.1	1.5	17904	Cistrome	70806
MYC	-1150.6	-1141.9	-1195.5	381.5	-6.7	318.3	0.0	8287	Cistrome	70805
MYC	-966.3	-940.6	-954.0	426.7	0.0	301.3	-0.5	11599	Cistrome	70810
MYC	-501.7	-538.9	-540.1	357.7	116.5	301.3	127.9	48637	Cistrome	70808
MYC	-407.9	-412.4	-403.5	163.6	-0.3	107.5	0.5	1306	Cistrome	76186
NIPBL	-578.5	-575.8	-568.9	357.0	0.8	355.4	18.0	29832	Cistrome	84953
NIPBL	-570.7	-562.9	-555.6	346.5	0.3	349.8	17.7	28604	Cistrome	88450
NR0B2	-133.1	-118.3	-101.0	4.6	-4.0	33.5	0.0	603	Cistrome	55660
PAF1	-713.0	-671.5	-658.2	279.0	-12.3	411.6	28.0	9761	Cistrome	88906
PAF1	-716.2	-735.7	-720.3	365.6	-2.7	389.7	10.0	26835	Cistrome	55667
PAF1	-738.2	-733.7	-719.3	334.2	-1.0	365.9	17.7	20824	Cistrome	84915
PAF1	-1236.5	-1214.6	-1235.9	182.7	-73.3	347.1	6.3	5762	Cistrome	55666
PAF1	-1061.6	-1066.0	-1043.8	339.7	-41.9	348.1	0.2	15947	Cistrome	55668
PAF1	-1017.9	-1019.0	-1016.2	328.0	-50.6	341.5	0.0	16156	Cistrome	55664
PAF1	-1261.5	-1233.2	-1184.1	311.9	-46.3	335.7	0.4	10352	Cistrome	55669
PAF1	-1180.3	-1262.1	-1233.7	278.7	-62.6	327.8	2.1	7895	Cistrome	55665
PHIP	-487.5	-491.6	-496.2	400.5	39.5	386.7	128.3	41242	Cistrome	85463
PHIP	-500.7	-507.5	-519.6	380.4	34.0	355.8	97.9	36060	Cistrome	85459
PHIP	-591.6	-612.5	-612.7	396.1	12.2	344.7	34.8	15366	Cistrome	85462
PHIP	-489.6	-585.1	-437.4	391.1	1.1	326.7	6.9	3676	Cistrome	87886
PHIP	-911.1	-977.0	-929.8	388.1	0.0	326.1	0.8	7263	Cistrome	87884
PHIP	-849.7	-885.9	-900.2	386.2	0.0	317.4	0.3	4636	Cistrome	85464
PHIP	-191.3	-176.5	-141.4	11.3	-23.5	67.8	-0.1	891	Cistrome	85465
RAD21	-333.5	-343.7	-329.8	365.2	138.3	380.4	172.6	91714	Cistrome	46207
RAD21	-709.2	-760.2	-743.3	430.4	174.4	384.7	208.0	67731	Cistrome	71320
RAD21	-669.5	-713.1	-709.9	439.5	202.9	373.6	217.8	98855	Cistrome	71322
RAD21	-439.8	-379.1	-342.8	380.1	58.8	365.7	264.1	6718	Cistrome	85290
RAD21	-644.1	-698.2	-685.1	419.1	201.4	348.2	197.7	90949	Cistrome	71321
Rad21	-314.6	-326.4	-303.6	316.7	ND	329.1	183.8	49942	Ensemble	homo_sapiens.GRCh38.HCT116.Rad21.SWEmbl_R0005.peaks.20190329.bed.gz
RAD21	-615.9	-664.5	-649.3	384.9	194.1	307.1	169.7	84470	Cistrome	71319
RAD21	-302.7	-313.5	-293.2	306.2	141.7	295.6	188.3	36976	Cistrome	81366
REST	-500.8	-511.1	-529.0	313.2	17.7	323.1	56.3	14210	Cistrome	46203
REST	-126.0	-132.9	-117.3	124.9	69.5	55.6	17.8	3483	Ensemble	homo_sapiens.GRCh38.HCT116.REST.SWEmbl_R0005.peaks.20190329.bed.gz
SIN3A	-710.8	-704.0	-695.4	337.4	-10.0	413.0	31.2	27435	Ensemble	homo_sapiens.GRCh38.HCT116.SIN3A.SWEmbl_R0005.peaks.20190329.bed.gz
SIN3A	-637.4	-636.8	-635.4	370.8	3.5	380.2	72.4	33224	Cistrome	46208
SIRT1	-684.0	-706.3	-707.2	334.6	252.0	252.3	163.9	39329	Cistrome	83885
SKP2	-636.7	-641.8	-669.3	348.0	282.9	277.2	162.3	100000	Cistrome	93781
SMARCA4	-152.2	-114.3	-127.6	107.0	2.8	242.1	1.0	7586	Cistrome	72102
SMARCA4	-22.5	-19.3	-12.4	0.1	-1.1	9.2	-0.5	767	Cistrome	72100
SMARCC1	-87.6	-74.9	-74.8	49.7	1.6	149.3	0.2	6118	Cistrome	72101
SMARCC1	-2.2	-0.8	-0.9	0.4	-0.5	13.9	-0.8	1081	Cistrome	72099
SMC1A	-454.4	-433.1	-396.4	331.4	17.3	324.4	39.9	7426	Cistrome	85288
SMC1A	-273.8	-286.0	-270.7	305.1	132.7	285.3	161.6	43636	Cistrome	85289
SP1	-1040.6	-1141.5	-1112.3	597.6	225.7	500.9	232.5	82625	Cistrome	5395
SP1	-441.7	-443.3	-438.7	391.5	110.2	427.3	173.0	72471	Cistrome	46215
SP1	-525.7	-524.2	-532.0	324.3	10.9	401.0	66.8	34242	Ensemble	homo_sapiens.GRCh38.HCT116.SP1.SWEmbl_R0005.peaks.20190329.bed.gz
SP1	-1147.5	-1179.0	-1125.4	420.6	0.1	387.6	23.3	21322	Cistrome	70813
SP1	-1078.5	-1143.4	-1125.6	452.5	11.9	386.6	44.3	24694	Cistrome	70811
SP1	-1255.1	-1387.0	-1295.7	438.2	0.2	382.0	10.1	19784	Cistrome	70812
SP1	-433.7	-462.8	-467.2	415.5	172.8	376.0	218.0	100000	Cistrome	70814
SP1	-557.1	-581.9	-592.5	421.5	39.3	304.1	15.2	30938	Cistrome	70815
SP1	-455.9	-494.8	-508.3	346.9	82.0	297.7	119.8	46179	Cistrome	70816
SRF	-463.2	-464.5	-455.0	379.4	101.1	402.6	167.9	57748	Cistrome	46211
Srf	-581.9	-563.4	-562.7	321.5	-142.5	399.9	112.4	28421	Ensemble	homo_sapiens.GRCh38.HCT116.Srf.SWEmbl_R0005.peaks.20190329.bed.gz
TCF4	-30.6	-16.5	-18.1	47.3	3.7	55.8	-0.1	3003	Cistrome	54661
TCF4	-26.4	-32.5	-26.0	33.4	2.3	43.5	0.0	1508	Cistrome	54669
TCF4	-27.3	-17.1	-18.9	34.7	4.0	36.1	0.0	1898	Cistrome	54664
TCF4	-17.4	-12.8	-14.5	27.0	2.6	34.8	0.0	1990	Cistrome	54667
TCF4	-35.6	-23.6	-20.2	23.4	2.1	28.0	-0.2	1825	Cistrome	54663
TCF4	-15.3	-18.3	-13.4	14.4	1.0	19.9	0.0	1043	Cistrome	54670
TCF4	-17.3	-10.2	-9.3	10.2	0.6	16.0	-0.2	872	Cistrome	54662
TCF7L1	-597.7	-645.1	-647.4	420.3	131.2	347.0	128.5	47979	Cistrome	70804
TCF7L1	-362.2	-372.5	-375.3	326.1	1.1	269.1	0.0	11321	Cistrome	70803
TCF7L1	-219.6	-218.8	-210.2	241.5	0.2	267.7	0.0	8191	Cistrome	70802
TCF7L2	-428.4	-417.7	-409.9	357.1	88.8	340.0	103.4	45234	Cistrome	45714
TCF7L2	-472.8	-460.2	-457.6	328.2	-133.7	323.2	3.1	20454	ENCODE	ENCFF199EHQ.bed.gz
TCF7L2	-461.7	-450.0	-446.8	328.0	-5.6	314.1	0.8	15431	ENCODE	ENCFF736EVD.bed.gz
TCF7L2	-347.6	-280.3	-252.6	290.4	272.6	288.7	0.0	4946	Ensemble	homo_sapiens.GRCh38.HCT116.TCF7L2.SWEmbl_R0005.peaks.20190329.bed.gz

TEAD4	-449.3	-450.6	-447.9	340.1	48.0	360.2	123.8	39773	Cistrome	46210
TEAD4	-423.1	-412.1	-408.6	251.5	-197.9	301.6	0.4	11796	Ensemble	homo_sapiens.GRCh38.HCT116.TEAD4.SWEmbl_R0005.peaks.20190329.bed.gz
TET2	-691.8	-748.7	-669.0	160.5	-26.6	267.0	0.2	3570	Cistrome	50981
TOP1	-339.6	-310.6	-298.8	309.6	88.3	266.6	126.4	11177	Cistrome	68774
TOP1	-183.9	-183.2	-187.6	264.0	27.2	236.9	50.1	7423	Cistrome	68781
TOP1	-168.5	-183.5	-184.0	302.1	25.7	168.6	36.0	4395	Cistrome	68775
TOP1	-255.0	-246.6	-252.7	321.9	16.5	123.7	17.4	3755	Cistrome	68780
TP53	-78.2	-78.6	-74.7	214.1	14.3	236.6	41.1	9393	Cistrome	82544
TP53	-21.2	-23.3	-26.1	45.2	5.0	47.4	2.8	3353	Cistrome	50345
TP53	-33.6	-30.0	-25.2	22.2	0.4	18.6	0.2	1004	Cistrome	92110
TP53	-14.4	-15.4	-15.3	29.7	2.0	12.5	0.5	781	Cistrome	53285
TP53	-17.9	-17.6	-23.9	30.4	2.7	12.5	ND	717	Cistrome	53286
TP53	-24.1	-24.5	-23.6	13.8	0.5	13.1	0.2	1368	Cistrome	82545
TP53	-14.0	-17.8	-31.9	32.7	4.4	5.9	0.0	1900	Cistrome	68462
TP53	-15.8	-19.2	-21.8	2.2	0.2	0.2	-0.2	807	Cistrome	50344
TP53	-5.0	-7.5	-17.9	5.9	1.1	0.0	-1.1	880	Cistrome	68461
TRIM28	-799.4	-878.3	-838.9	408.5	78.4	258.4	15.9	24209	Cistrome	57090
USF1	-474.1	-476.9	-481.6	384.6	118.5	397.2	173.0	57530	Cistrome	46205
USF1	-703.1	-689.1	-718.1	332.1	-0.2	387.6	61.2	9985	Ensemble	homo_sapiens.GRCh38.HCT116.USF1.SWEmbl_R0005.peaks.20190329.bed.gz
USP49	-134.5	-131.3	-99.0	108.1	16.5	75.3	0.8	2124	Cistrome	39345
USP49	-99.3	-109.3	-80.3	16.8	5.3	5.8	0.9	1350	Cistrome	39347
YY1	-675.4	-667.8	-681.1	353.8	40.7	392.2	66.5	17804	ENCODE	ENCFF094BQZ.bed.gz
YY1	-720.9	-716.2	-739.7	309.3	172.4	390.6	34.3	7329	ENCODE	ENCFF279EVE.bed.gz
YY1	-503.8	-507.8	-502.6	381.6	72.2	381.9	143.4	45823	Cistrome	46204
Yy1	-857.0	-855.5	-817.9	284.5	-18.3	387.3	15.0	4670	Ensemble	homo_sapiens.GRCh38.HCT116.Yy1.SWEmbl_R0005.peaks.20190329.bed.gz
ZBTB33	-776.5	-775.7	-737.2	293.9	-19.3	374.4	0.7	5886	ENCODE	ENCFF422MCZ.bed.gz
ZBTB33	-660.5	-637.7	-649.0	313.2	-1.3	365.2	8.3	13174	Cistrome	46200
ZBTB33	-362.0	-371.1	-334.6	115.1	55.2	302.3	0.4	2206	ENCODE	ENCFF795HVQ.bed.gz
ZBTB33	-252.4	-273.9	-174.7	23.1	-321.5	96.7	0.1	1435	Ensemble	homo_sapiens.GRCh38.HCT116.ZBTB33.SWEmbl_R0005.peaks.20190329.bed.gz
ZC3H8	-38.6	-58.3	-28.9	222.1	6.6	247.1	32.2	5255	Cistrome	39918
ZFX	-1164.7	-1207.1	-1181.3	388.8	-5.8	396.9	10.5	23446	ENCODE	ENCFF215SIC.bed.gz
ZFX	-768.2	-747.7	-771.8	345.0	-3.6	336.6	0.8	17139	Cistrome	81159
ZFX	-1377.6	-1448.1	-1507.3	399.4	0.0	323.6	0.9	15109	ENCODE	ENCFF897IRP.bed.gz
ZFX	-362.4	-393.0	-388.2	145.2	95.0	151.0	108.9	100000	Cistrome	81160
ZNF274	-28.4	-24.8	-20.5	-170.4	2.6	-65.4	-55.6	1079	Ensemble	homo_sapiens.GRCh38.HCT116.ZNF274.SWEmbl_R0005.peaks.20190329.bed.gz

ChIP-seq for H3K36me3 histone marker in non-treated and RTX treated UGI-expressing HCT116 cells.

To check, if drug treatment affects the histone marker distribution, a ChIP-seq experiment for H3K36me3 marker was performed on non-treated and RTX treated HCT116 cells that were also expressing UGI (cf. Materials and Methods). The pre-processing of the raw data was performed in the same way as in case of the U-DNA-Seq applying the HCT116 specific blacklist (cf. Supplementary file 1 and Figure 2-figure supplement 2). The statistics on these pre-processing steps are provided here in Supplementary file 3-table 2.

Supplementary file 3-table 2. Statistics on pre-processing of H3K36me3 ChIP-seq data.

sample	number of raw reads	number of mapped reads	unmapped reads		uniquely mapped reads		uniquely mapped reads after blacklisting	
			number	%	number	%	number	%
**NT_UGI H3K36me3 ChIP-seq**	126640549	126515616	124933	0.10	119567538	94.41	114315230	90.27
**RTX_UGI H3K36me3 ChIP-seq**	170421288	170127897	293391	0.17	162137011	95.14	155810499	91.43

The peak calling was performed by MACS2 using broad peak option as given below using an input control selected from the ENCODE (ENCFF489VMD).

```
$ macs2 callpeak -t NAME(IP).filtered_blacklisted.bam -c
ENCFF489VMD.filtered_blacklisted.bam --broad -g 2830361285 --broad-cutoff 0.05 -n
NAME_ChIPvsCtr_0p05 --outdir {PATH} --nomodel -f BAMPE --cutoff-analysis
```

The fold change tracks were also calculated comparing to an input control as given below.

```
$ bamCoverage -b NAME.filtered_blacklisted.bam -o NAME.bin100.RPGC.bw --binSize 100
--verbose --normalizeUsing RPGC --effectiveGenomeSize 2792809237 -p 32 --extendReads
```

```
$ bigwigCompare -b1 NAME.bin100.RPGC.bw -b2
ENCFF489VMD.filtered_blacklisted.bin100.RPGC.bw -o
NAME_foldChange.bin100bp.RPGC.ratio.bw -of bigwig --binSize 100 -v -p 32 --skipNAs -
-operation ratio
```

These processed files as well as the corresponding raw data were uploaded to the GEO (GSE153407, that is combined with the U-DNA-Seq data under GSE153408). These were compared to each other as well as to the H3K36me3 ChIP-seq foldChange tracks downloaded from the ENCODE using multiBigWigSummary (Figure 4-figure supplement 1).

```
$ multiBigwigSummary bins -b ENCFF238GBP.bigWig  ENCFF334KFI.bigWig
ENCFF514ZYW.bigWig NT_UGI_H3K36me3_ChIP_foldChange.bin100bp.RPGC.ratio.bw
RTX_UGI_H3K36me3_ChIP_foldChange.bin100bp.RPGC.ratio.bw -o
mbws_Fig4FigSup1_H3K36me3ChIPseq.npz -v -p 32
```

```
$ plotCorrelation --corData mbws_Fig4FigSup1_H3K36me3ChIPseq.npz --corMethod pearson
--whatToPlot heatmap -o mbws_Fig4FigSup1_H3K36me3ChIPseq.png -T
mbws_Fig4FigSup1_H3K36me3ChIPseq --skipZeros --removeOutliers --plotNumbers --
colorMap RdPu
```

GIGGLE scores between U-DNA-seq results and these H3K36me3 ChIP-seq peaks were calculated as it was described for other ChIP-seq data above (Figure 4A, Figure 4-source data 1).

The fold change track files were also included in the Segway analysis (Figure 4B, Figure 4-source data 2).

Genome segmentation analysis of U-DNA-Seq results and ChIP-seq data from the ENCODE using Segway genome segmentation algorithm.

22 independent ChIP-seq experiments available for HCT116 cells in the ENCODE database were selected for this type of analysis (see Supplementary file 3-table 3). The corresponding 'fold change over control' tracks were combined with our ChIP-seq data on H3K36me3 as well as with our U-DNA-Seq data to a single genomedata file using Genomedata software (Hoffman, Buske, & Noble, 2010). In this analysis, merged ratio tracks of U-DNA enrichment were used that were calculated by bigwigCompare of the deepTools package (Ramírez et al., 2016) in the format of bw files and converted to BedGraph format. File conversions were done using BigWigToWig tool (Kuhn, Haussler, & Kent, 2013). The genome

segmentation analysis was confined to the core chromosomes (1 to 22, X, and Y) of GRCh38 reference genome, and the regions of our HCT116 specific blacklist were also excluded. The applied command lines are provided below.

File conversion and filtration in case of bigWig files from the ENCODE database:

```
$ bigWigToWig NAME.bigwig NAME.wig
```

```
$ awk '{ if ($1 == "chr1" || $1 == "chr2" || $1 == "chr3" || $1 == "chr4" || $1 ==
"chr5" || $1 == "chr6" || $1 == "chr7" || $1 == "chr8" || $1 == "chr9" || $1 ==
"chr10" || $1 == "chr11" || $1 == "chr12" || $1 == "chr13" || $1 == "chr14" || $1 ==
"chr15"  || $1 == "chr16" || $1 == "chr17" || $1 == "chr18" || $1 == "chr19" || $1
== "chr20" || $1 == "chr21" || $1 == "chr22" || $1 == "chrX" || $1 == "chrY") {print
$0} }' NAME.wig > NAME.filtered.wig
```

File conversion and filtration in case of bw files of U-DNA-Seq ratio tracks:

```
$ bigWigToWig NAME.bw NAME.bdg
```

```
$ awk '{ if ($1 == "chr1" || $1 == "chr2" || $1 == "chr3" || $1 == "chr4" || $1 ==
"chr5" || $1 == "chr6" || $1 == "chr7" || $1 == "chr8" || $1 == "chr9" || $1 ==
"chr10" || $1 == "chr11" || $1 == "chr12" || $1 == "chr13" || $1 == "chr14" || $1 ==
"chr15"  || $1 == "chr16" || $1 == "chr17" || $1 == "chr18" || $1 == "chr19" || $1
== "chr20" || $1 == "chr21" || $1 == "chr22" || $1 == "chrX" || $1 == "chrY") {print
$0} }' NAME.bdg > NAME.filtered.bdg
```

Preparing genomedata file using genomedata-load (Hoffman et al., 2010):

```
$ genomedata-load -t {SAMPLE1}={FILE1} -t {SAMPLE2}={FILE2} … -s core_regions.fna
mergedload
```

The genome data was trained by the segway train (Chan et al., 2017; Hoffman et al., 2012) where the number of labels (types of characteristic patterns) was defined as 25, also providing the same blacklist that was used for the U-DNA-Seq data.

```
$ SEGWAY_CLUSTER=local segway train mergedload --resolution=100 --num-labels=25 --
minibatch-fraction=0.01 --exclude-coords=blacklist_merged_d500.bed
train25_merged_blacklisted
```

Then genomic segments with these labels were identified with segway annotate (Chan et al., 2017; Hoffman et al., 2012):

```
$ SEGWAY_CLUSTER=local segway annotate mergedload train25_merged_blacklisted
Segway_annot_out
```

The signal distribution was calculated using Segtools (Buske, Hoffman, Ponts, Le Roch, & Noble, 2011) and was plotted by Seaborn (MatPlotLib modul in python, (Hunter, 2007)) (cf. Figure 4B).

```
$ segtools-signal-distribution segway.bed mergedload

import pandas as pd
import seaborn as sb
```

```
df = pd.read_csv("Segway norm_data.txt", delimiter="\t")
df = df.set_index(df.columns[0])
s=sb.heatmap(df,xticklabels=True,yticklabels=True,linewidths=0.5,linecolor="black",cmap="RdYlBu_r",annot=False,annot_kws={'size':4})
figure = s.get_figure()
figure.savefig('plot_v.png', dpi=400)
exit()
```

Supplementary file 3-table 3: The composition of the genomedata file.

{LABEL}	{FILE}
WT	WT_HCT116_merged_IP_vs_son.bin100bp.smooth5000.RPGC.ratio.filtered.bdg
NT_UGI	NT_UGI_HCT116_merged_IP_vs_son.bin100bp.smooth5000.RPGC.ratio.filtered.bdg
NT_UGI_MMR	NT_UGI_HCT116MMR_merged_IP_vs_son.bin100bp.smooth5000.RPGC.ratio.filtered.bdg
5FdUR_UGI	5FdUR_UGI_HCT116_merged_IP_vs_son.bin100bp.smooth5000.RPGC.ratio.filtered.bdg
5FdUR_UGI_MMR	5FdUR_UGI_HCT116MMR_merged_IP_vs_son.bin100bp.smooth5000.RPGC.ratio.filtered.bdg
RTX_UGI	RTX_UGI_HCT116_merged_IP_vs_son.bin100bp.smooth5000.RPGC.ratio.filtered.bdg
RTX_UGI_MMR	RTX_UGI_HCT116MMR_merged_IP_vs_son.bin100bp.smooth5000.RPGC.ratio.filtered.bdg
EZH2_pT487	ENCFF011JWE.filtered.wig
H3K79me2	ENCFF127XQD.filtered.wig
H3K4me3	ENCFF144ZRX.filtered.wig
ZFX	ENCFF168KEG.filtered.wig
H3K9ac	ENCFF187IFT.filtered.wig
H2AFZ	ENCFF236ZOJ.filtered.wig
TCF7L2	ENCFF241JHM.filtered.wig
YY1	ENCFF293WBY.filtered.wig
JUND	ENCFF415KJH.filtered.wig
H4K20me1	ENCFF431PNC.filtered.wig
H3K36me3	ENCFF514ZYW.filtered.wig
H3K9me3	ENCFF542HPZ.filtered.wig
EZH2	ENCFF604ZUW.filtered.wig
ZBTB33	ENCFF616IIZ.filtered.wig
CTFC	ENCFF620LDT.filtered.wig
H3K4me1	ENCFF774BWO.filtered.wig
H3K4me2	ENCFF783QRO.filtered.wig
RNApol2A_pS5	ENCFF794LVU.filtered.wig
H3K9me2	ENCFF807PSQ.filtered.wig
RNApol2A	ENCFF856HUR.filtered.wig
H3K27me3	ENCFF984BVG.filtered.wig
H3K27ac	ENCFF984WLE.filtered.wig
NT_H3K36me3	NT_UGI_H3K36me3_ChIP_foldChange.bin100bp.RPGC.ratio.filtered.bdg
RTX_H3K36me3	RTX_UGI_H3K36me3_ChIP_foldChange.bin100bp.RPGC.ratio.filtered.bdg

References

Buske, O. J., Hoffman, M. M., Ponts, N., Le Roch, K. G., & Noble, W. S. (2011). Exploratory analysis of genomic segmentations with Segtools. *BMC Bioinformatics*, *12*(1), 415. https://doi.org/10.1186/1471-2105-12-415

Chan, R. C. W., Libbrecht, M. W., Roberts, E. G., Bilmes, J. A., Noble, W. S., & Hoffman, M. M. (2017). Segway 2.0: Gaussian mixture models and minibatch training. *Bioinformatics*, *34*(4), 669–671. https://doi.org/10.1093/bioinformatics/btx603

ENCODE Project Consortium. (2012). An integrated encyclopedia of DNA elements in the human genome. *Nature*, *489*(7414), 57–74. https://doi.org/10.1038/nature11247

Hayden, K. E., Strome, E. D., Merrett, S. L., Lee, H.-R., Rudd, M. K., & Willard, H. F. (2013). Sequences Associated with Centromere Competency in the Human Genome. *Molecular and Cellular Biology*, *33*(4), 763–772. https://doi.org/10.1128/MCB.01198-12

Hoffman, M. M., Buske, O. J., & Noble, W. S. (2010). The Genomedata format for storing large-scale functional genomics data. *Bioinformatics (Oxford, England)*, *26*(11), 1458–1459. https://doi.org/10.1093/bioinformatics/btq164

Hoffman, M. M., Buske, O. J., Wang, J., Weng, Z., Bilmes, J. A., & Noble, W. S. (2012). Unsupervised pattern discovery in human chromatin structure through genomic segmentation. *Nature Methods*, *9*(5), 473–476. https://doi.org/10.1038/nmeth.1937

Hunter, J. D. (2007). Matplotlib: A 2D graphics environment. *Computing in Science & Engineering*, *9*(3), 90–95.

Kuhn, R. M., Haussler, D., & Kent, W. J. (2013). The UCSC genome browser and associated tools. *Briefings in Bioinformatics*, *14*(2), 144–161. https://doi.org/10.1093/bib/bbs038

Kundaje, A., Meuleman, W., Ernst, J., Bilenky, M., Yen, A., Heravi-Moussavi, A., … Kellis, M. (2015). Integrative analysis of 111 reference human epigenomes. *Nature*, *518*(7539), 317–330. https://doi.org/10.1038/nature14248

Layer, R. M., Pedersen, B. S., DiSera, T., Marth, G. T., Gertz, J., & Quinlan, A. R. (2018). GIGGLE: a search engine for large-scale integrated genome analysis. *Nature Methods*, *15*(2), 123–126. https://doi.org/10.1038/nmeth.4556

Mei, S., Qin, Q., Wu, Q., Sun, H., Zheng, R., Zang, C., … Liu, X. S. (2017). Cistrome Data Browser: a data portal for ChIP-Seq and chromatin accessibility data in human and mouse. *Nucleic Acids Research*, *45*(D1), D658–D662. https://doi.org/10.1093/nar/gkw983

Ramírez, F., Ryan, D. P., Grüning, B., Bhardwaj, V., Kilpert, F., Richter, A. S., … Manke, T. (2016). deepTools2: a next generation web server for deep-sequencing data analysis. *Nucleic Acids Research*, *44*(W1), W160-5. https://doi.org/10.1093/nar/gkw257

Shu, X., Liu, M., Lu, Z., Zhu, C., Meng, H., Huang, S., … Yi, C. (2018). Genome-wide mapping reveals that deoxyuridine is enriched in the human centromeric DNA. *Nature Chemical Biology*, *14*(7), 680–687. https://doi.org/10.1038/s41589-018-0065-9

Zerbino, D. R., Achuthan, P., Akanni, W., Amode, M. R., Barrell, D., Bhai, J., … Flicek, P. (2018). Ensembl 2018. *Nucleic Acids Research*, *46*(D1), D754–D761. https://doi.org/10.1093/nar/gkx1098
