# Supplementary material for: Genome-wide alterations of uracil distribution patterns in human DNA upon chemotherapeutic treatments
Source: eLife. 2020 Sep 21;9:e60498. doi: 10.7554/eLife.60498 (PMC7505663; doi:10.7554/eLife.60498)
Supplement: Supplementary file 4. — Database information, applied command lines, full results (table 1), and calculation of replication timing scores and AT content on genomic segments (from the Segway analysis) are provided. [file elife-60498-supp4.pdf]

Supplementary file 4

Genome-wide analysis of uracil-DNA pattern comparing to other genomic features using bedtools annotate

We also wanted to measure colocalization with other genomic features such as cytogenetic bands, coding vs non-coding regions, repeat classes, or replication timing. Data were collected from UCSC Table Browser ((Karolchik et al., 2004) http://genome.ucsc.edu/cgi-bin/hgTables), as it is labelled in Supplementary file 4-table 1. These tab delimited text files were rearranged to fulfil the minimum requirements of the bed format, and sub-categories were also selected into separated bed files using awk.

Replication timing data specific for HCT116 cells were obtained from Replication Domain database (Int90617792 and Int97243322, https://www2.replicationdomain.com/database.php (Weddington et al., 2008)). From these wiggle files, early, middle and late replicating segments were extracted to bed files as follows:

```
$ awk ' $4 > 2 ' Int90617792.wig > Int90617792_early_RT.bed
$ awk ' $4 < 0 ' Int90617792.wig > Int90617792_neg_RT.wig
$ awk ' $4 > -2 ' Int90617792_neg_RT.wig > Int90617792_late_RT.wig
```

# Note that awk use ”>” for the absolute value of the negative numbers.

```
$ awk ' $4 > 0 ' Int90617792.wig > Int90617792_pos_RT.wig
$ awk ' $4 <= 2 ' Int90617792_pos_RT.bed > Int90617792_mid_pos_RT.bed
$ awk ' $4 <= -2 ' Int90617792_neg_RT.bed > Int90617792_mid_neg_RT.bed
$ cat Int90617792_mid_pos_RT.bed Int90617792_mid_neg_RT.bed | sort -k1,1 -k2,2n >
Int90617792_mid_RT_sorted.bed
```

# From this state, the processing steps are the same, as in case of the log2 track derived bed files involving bedtools merge, bigWigAverageOverBed, sort and awk.

Additional data were collected from Ensembl database (Zerbino et al., 2018): genomic annotations (ftp://ftp.ensembl.org/pub/release-97/gff3/homo_sapiens/Homo_sapiens.GRCh38.97.gff3.gz) and HCT116 specific regulatory features corresponding to transcriptional activity (ftp://ftp.ensembl.org/pub/release-97/regulation/homo_sapiens/RegulatoryFeatureActivity/HCT116/homo_sapiens.GRCh38.HCT116.Regulatory_Build.regulatory_activity.20190329.gff.gz). From this latter file, relevant interval files corresponding to different categories (e. g. promoter, enhancer, etc., cf. Supplementary file 4-table 1) were derived as follows:

```
$ awk '{gsub(/\;/, "\t", $9)} {print "chr"$1 "\t" $4 "\t"$5 "\t"$3 "\t" $9}'
homo_sapiens.GRCh38.HCT116.Regulatory_Build.regulatory_activity.20190329.gff >
homo_sapiens.GRCh38.HCT116.Regulatory_Build.regulatory_activity_separated.20190329.gff.bed
```

```
$ awk '$5=="activity=ACTIVE" {print $1 "\t" $2 "\t"$3 "\t"$4 "\t" $5}'
homo_sapiens.GRCh38.HCT116.Regulatory_Build.regulatory_activity_separated.20190329.gff.bed
> homo_sapiens.GRCh38.HCT116.Regulatory_Build.regulatory_activity_ACTIVE.20190329.gff.bed
```

```
$ awk '$4=="promoter" {print $1 "\t" $2 "\t"$3 "\t"$4 "\t" $5}'
homo_sapiens.GRCh38.HCT116.Regulatory_Build.regulatory_activity_ACTIVE.20190329.gff.bed >
homo_sapiens.GRCh38.HCT116.Regulatory_Build.regulatory_activity_PROMOTER.20190329.gff.bed
```

Data for RNA genes (cf. long non-coding RNAs (lnc_RNA)) were similarly derived from the Homo_sapiens.GRCh38.97.gff3 file.

To further access the alpha satellites and the assembled higher order repeat segments (HORs), another interval file corresponding to the publication (Uralsky et al., 2019) was also downloaded (https://genome.ucsc.edu/cgi-bin/hgTrackUi?hgsid=771843343_kD7KrH9deXCkpCCcKNTjvq4t3jOi&c=chr1&g=ct_HMMERSF1HORst281_7253).

These features often contain too many and/or too large intervals for which, GIGGLE 1.0 (Layer et al., 2018) was not proved to be efficient (cf. issue #46 https://github.com/ryanlayer/GIGGLE/issues/46). Therefore, bedtools annotate (Quinlan & Hall, 2010) was applied to count the overlaps between the U-DNA-Seq and the database intervals. The numbers of overlapping bases between each sample and each database interval file were summarized and scores were calculated according to the following formula:

$$\frac{N^{o}\ of\ overlaping\ bases\ *\ 100}{N^{o}\ of\ bases\ in\ sample\ intervals} * \frac{N^{o}\ of\ overlaping\ bases\ *\ 100}{N^{o}\ of\ bases\ in\ feature\ intervals}$$

A systematic selection of the tested features is shown in Figure 4C, while the results of the full analysis are provided in Supplementary file 4-table 1. Bedtools annotate was used as follows:

```
$ bedtools annotate –names {short names for the database interval files} -both -i
NAME.filtered_blacklisted.bin100bp.smooth5k.RPGC.log2.0p2.region.bed -files {list of
database interval files} > bedtools_annot.NAME.bed
```

# For one representative database interval file, the number of overlapping bases is calculated as follows:

```
$ awk -v OFS="\t" '{$8=$3-$2}1' bedtools_annot_NAME.bed | awk '{($7=$7*$8)}1' | awk
'{(sum1 += $7)} END {print sum1}' > bedtools_annot_NAME_results.csv
```

		WT	NT_UGI	NT_UGI MMR	5FdUR_UGI	5FdUR_UGI MMR	RTX_UGI	RTX_UGI MMR	
	baseNo in intervals	*3.19E+08*	*3.28E+08*	*3.19E+08*	*5.25E+08*	*5.26E+08*	*5.21E+08*	*5.18E+08*	
**Protein Gene**	*1.72E+09*	**4.39E+02**	**4.43E+02**	**4.37E+02**	**8.51E+02**	**8.85E+02**	**1.28E+03**	**1.30E+03**	
**INTRON**	*1.64E+09*	**4.35E+02**	**4.41E+02**	**4.33E+02**	**7.60E+02**	**8.34E+02**	**1.15E+03**	**1.18E+03**	
**EXON**	*1.46E+08*	**1.26E+01**	**1.18E+01**	**1.22E+01**	**2.03E+02**	**9.32E+01**	**3.02E+02**	**2.61E+02**	[1]
**CDS**	*3.97E+07*	**2.83E+00**	**2.72E+00**	**2.78E+00**	**5.60E+01**	**2.16E+01**	**9.35E+01**	**7.71E+01**	
**UTR**	*1.26E+08*	**1.11E+01**	**1.04E+01**	**1.08E+01**	**1.73E+02**	**7.91E+01**	**2.66E+02**	**2.31E+02**	
**RNA genes**	*1.29E+06*	**1.32E-01**	**1.39E-01**	**1.22E-01**	**1.29E+00**	**5.60E-01**	**1.77E+00**	**1.63E+00**	
**srpRNA**	*2.91E+05*	**1.68E-02**	**2.13E-02**	**1.66E-02**	**3.64E-01**	**1.31E-01**	**7.31E-01**	**6.66E-01**	
**snRNA**	*3.66E+05*	**6.43E-02**	**7.33E-02**	**5.83E-02**	**3.40E-01**	**1.27E-01**	**5.98E-01**	**5.87E-01**	
**scRNA**	*1.40E+05*	**1.15E-02**	**8.90E-03**	**1.04E-02**	**1.60E-01**	**3.76E-02**	**3.58E-01**	**3.02E-01**	[2]
**tRNA**	*1.22E+05*	**8.10E-03**	**8.89E-03**	**7.80E-03**	**2.12E-01**	**8.63E-02**	**1.26E-01**	**7.26E-02**	
**rRNA**	*2.55E+05*	**1.59E-02**	**1.36E-02**	**1.36E-02**	**1.79E-01**	**1.14E-01**	**9.26E-02**	**9.40E-02**	
**RNA**	*1.18E+05*	**2.53E-02**	**2.48E-02**	**2.51E-02**	**6.31E-02**	**7.47E-02**	**5.87E-02**	**7.14E-02**	
**lincRNA (colon)**	*2.01E+08*	**9.81E+01**	**1.03E+02**	**9.69E+01**	**1.23E+02**	**1.83E+02**	**5.78E+01**	**5.77E+01**	[3]
**lnc_RNA**	*1.08E+09*	**3.51E+02**	**3.61E+02**	**3.49E+02**	**5.16E+02**	**6.25E+02**	**7.12E+02**	**7.15E+02**	[4]
**miRNA**	*3.03E+04*	**1.99E-03**	**1.77E-03**	**1.86E-03**	**6.51E-02**	**3.24E-02**	**6.72E-02**	**3.78E-02**	[5]
**mi_snoRNA**	*1.95E+05*	**1.81E-02**	**1.67E-02**	**1.54E-02**	**3.33E-01**	**1.53E-01**	**4.47E-01**	**2.88E-01**	[6]
**tRNAs**	*4.68E+04*	**5.02E-05**	**1.62E-04**	**2.11E-04**	**2.30E-01**	**6.45E-02**	**1.10E-01**	**3.22E-02**	[7]
**CpGIsland (unmasked)**	*3.36E+07*	**2.34E-01**	**1.99E-01**	**1.99E-01**	**9.60E+01**	**2.34E+01**	**5.04E+01**	**1.54E+01**	[8]
**rmsk_LINE**	*6.72E+08*	**3.81E+02**	**4.33E+02**	**3.73E+02**	**1.77E+02**	**1.96E+02**	**2.00E+02**	**2.09E+02**	
**rmsk_SINE**	*4.17E+08*	**3.59E+01**	**3.33E+01**	**3.24E+01**	**4.54E+02**	**1.99E+02**	**6.99E+02**	**5.82E+02**	
**rmsk_LTR**	*2.82E+08*	**3.68E+01**	**3.52E+01**	**3.74E+01**	**1.58E+02**	**2.52E+02**	**9.70E+01**	**1.24E+02**	
**rmsk_Retroposon**	*4.54E+06*	**1.74E-02**	**1.20E-02**	**7.35E-03**	**7.06E+00**	**1.71E+00**	**5.45E+00**	**3.69E+00**	
**rmsk_DNA**	*1.07E+08*	**4.28E+01**	**4.07E+01**	**4.28E+01**	**5.66E+01**	**6.87E+01**	**5.55E+01**	**6.78E+01**	
**rmsk_DNAq**	*4.59E+05*	**2.81E-01**	**2.74E-01**	**2.95E-01**	**1.21E-01**	**3.25E-01**	**7.61E-02**	**1.11E-01**	[1]
**rmsk_Helitron**	*3.81E+05*	**2.34E-01**	**2.09E-01**	**2.26E-01**	**1.82E-01**	**2.10E-01**	**1.42E-01**	**1.75E-01**	
**rmsk_Satellite**	*7.89E+07*	**3.02E-01**	**2.64E-01**	**3.19E-01**	**1.60E-01**	**6.29E-01**	**7.28E-02**	**5.65E-02**	
**rmsk_Simple_repeat**	*3.95E+07*	**2.39E+01**	**2.31E+01**	**2.30E+01**	**2.67E+01**	**2.40E+01**	**1.97E+01**	**1.37E+01**	
**rmsk_Low_complexity**	*6.39E+06*	**2.31E+00**	**2.33E+00**	**2.07E+00**	**4.81E+00**	**3.69E+00**	**3.53E+00**	**2.36E+00**	
**rmsk_Unknown**	*7.53E+05*	**3.57E-01**	**3.48E-01**	**3.95E-01**	**2.93E-01**	**6.35E-01**	**1.73E-01**	**2.39E-01**	
**simpleRepeat**	*1.49E+08*	**7.15E+00**	**6.71E+00**	**6.83E+00**	**3.48E+01**	**2.33E+01**	**3.64E+01**	**2.24E+01**	[9]
**nestedRepeat**	*8.89E+08*	**3.13E+02**	**3.43E+02**	**3.03E+02**	**4.26E+02**	**3.73E+02**	**4.99E+02**	**4.91E+02**	[10]
**microsatellites**	*1.70E+06*	**8.26E-01**	**8.14E-01**	**7.53E-01**	**1.01E+00**	**9.57E-01**	**9.28E-01**	**8.66E-01**	[11]
**centromeres**	*5.95E+07*	**1.02E-05**	**8.01E-06**	**9.95E-05**	**0.00E+00**	**7.37E-05**	**0.00E+00**	**7.78E-06**	[12]
**Satellite_centro_repeats**	*7.42E+07*	**2.76E-04**	**6.25E-05**	**3.74E-04**	**5.89E-03**	**3.64E-02**	**5.27E-04**	**1.14E-03**	[1]
**HORs**	*2.04E+07*	**1.53E-08**	**3.59E-08**	**3.69E-08**	**0.00E+00**	**4.65E-05**	**2.26E-08**	**2.36E-05**	(23)
**cytoBand_gneg**	*1.47E+09*	**1.33E+02**	**1.33E+02**	**1.31E+02**	**1.42E+03**	**1.09E+03**	**1.53E+03**	**1.43E+03**	
**cytoBand_gpos25**	*2.14E+08*	**1.07E+01**	**1.07E+01**	**1.07E+01**	**3.05E+02**	**2.10E+02**	**4.21E+02**	**3.65E+02**	
**cytoBand_gpos50**	*4.10E+08*	**6.45E+01**	**6.52E+01**	**6.55E+01**	**2.12E+02**	**3.00E+02**	**1.78E+02**	**2.06E+02**	[13]
**cytoBand_gpos75**	*4.11E+08*	**3.33E+02**	**3.41E+02**	**3.33E+02**	**8.41E+01**	**1.58E+02**	**4.09E+01**	**6.50E+01**	
**cytoBand_gpos100**	*4.96E+08*	**1.07E+03**	**1.12E+03**	**1.07E+03**	**1.86E+01**	**7.19E+01**	**1.00E+01**	**1.41E+01**	
**early_RT1**	*5.80E+08*	**6.03E+00**	**5.37E+00**	**5.16E+00**	**1.85E+03**	**2.70E+02**	**4.04E+03**	**3.43E+03**	
**early_RT2**	*5.74E+08*	**5.96E+00**	**5.21E+00**	**5.09E+00**	**1.80E+03**	**2.53E+02**	**4.03E+03**	**3.40E+03**	
**middle_RT1**	*1.02E+09*	**1.66E+02**	**1.65E+02**	**1.65E+02**	**8.64E+02**	**1.87E+03**	**5.10E+01**	**1.77E+02**	[14]
**middle_RT2**	*1.03E+09*	**1.75E+02**	**1.74E+02**	**1.73E+02**	**8.83E+02**	**1.86E+03**	**5.33E+01**	**1.84E+02**	
**late_RT1**	*6.19E+08*	**5.01E+02**	**5.23E+02**	**5.04E+02**	**1.50E+00**	**1.65E+02**	**1.08E-04**	**2.41E-02**	
**late_RT2**	*6.09E+08*	**4.85E+02**	**5.01E+02**	**4.87E+02**	**1.76E+00**	**1.79E+02**	**1.14E-04**	**1.69E-02**	
**CTCF_bind**	*4.86E+07*	**3.97E+00**	**3.70E+00**	**4.00E+00**	**9.90E+01**	**5.87E+01**	**8.88E+01**	**7.42E+01**	
**TF_bind**	*3.25E+06*	**3.95E-01**	**3.74E-01**	**3.94E-01**	**4.43E+00**	**3.67E+00**	**3.74E+00**	**3.36E+00**	
**ENHANCER**	*1.25E+06*	**8.68E-02**	**1.05E-01**	**9.81E-02**	**1.41E+00**	**4.68E-01**	**3.98E+00**	**1.51E+00**	[15]
**PROMOTER**	*2.28E+07*	**1.05E-01**	**9.87E-02**	**9.53E-02**	**6.50E+01**	**1.51E+01**	**5.58E+01**	**1.82E+01**	
**PROMOTER_flank**	*2.48E+07*	**6.77E-01**	**6.89E-01**	**6.08E-01**	**3.04E+01**	**1.45E+01**	**7.47E+01**	**2.78E+01**	
**OPEN**	*9.71E+04*	**1.52E-02**	**1.80E-02**	**2.64E-02**	**1.12E-01**	**1.22E-01**	**1.15E-01**	**1.33E-01**	
**DNAseHS_HCT116**	*2.35E+07*	**7.47E-01**	**7.14E-01**	**7.57E-01**	**4.78E+01**	**1.82E+01**	**4.80E+01**	**2.11E+01**	[16]
**RegDnaseCluster**	*4.58E+08*	**4.70E+01**	**4.63E+01**	**4.74E+01**	**6.64E+02**	**4.93E+02**	**6.03E+02**	**5.00E+02**	[17]

Supplementary file 4-table 1. **Full collection of genomic features compared to samples of U-DNA-Seq.** Genomic features were downloaded from the UCSC (Karolchik et al., 2004), the Ensembl (Zerbino et al., 2018), and the Replication Domain (Weddington et al., 2008) databases. The detailed sources are indicated in the last column as follows: [1] UCSC, Table Browser: Genes and Gene Predictions / GENCODE v32 / knownGene, [2] UCSC Table Browser: Repeats / RepeatMasker / rmsk, [3] UCSC Table Browser: Genes and Gene Predictions / lincRNA RNA-Seq / Colon (lincRNAsCTColon), [4] Ensembl, [5] UCSC Table Browser: Expression / miRNA Tissue Atlas / sample1 (miRnaAtlasSample1BarChart), [6] UCSC Table Browser: Genes and Gene Predictions / sno/miRNA / wgRna, [7] UCSC Table Browser: Genes and Gene Predictions / tRNA Genes / tRNAs, [8] UCSC Table Browser: Regulation / Unmasked CpG / cpgIslandExtUnmasked, [9] UCSC Table Browser: Repeats / Simple Repeats / simpleRepeat, [10] UCSC Table Browser: Repeats / Interrupted Rpts / nestedRepeats, [11] UCSC Table Browser: Repeats / Microsatellite / microsat, [12] UCSC Table Browser: Mapping and Sequencing / Centromeres / centromeres, [13] UCSC Table Browser: Mapping and Sequencing / Chromosome Band / cytoBand, [14] Replication Domain Database, [15] Ensembl, regulatory build for HCT116, [16] UCSC Table Browser: Regulation / DNase HS / HCT-116 Pk (wgEncodeRegDnaseUwHct116Peak), [17] UCSC Table Browser: Regulation / DNase Clusters / wgEncodeRegDnaseClustered. Higher order repeat segments (HORs) were downloaded from UCSC (HMMERSF1HORst281top100k.bed, (Uralsky et al., 2019)). Scores were calculated according the formula given in the text. Abbreviated features are the following: coding sequences (CDS), untranslated regions (UTR), signal recognition particle RNAs (srpRNA), small nuclear (snRNA), small conditional (scRNA), long non-coding RNA (lncRNA), long intergenic non-coding RNAs found in colon tissues (lincRNA (colon)), micro-RNA (miRNA), micro and small nucleolar (mi_snoRNA) RNAs, short interspersed nuclear elements (SINE) and long interspersed nuclear elements (LINE), long terminal repeat element (LTR), putative DNA repeat elements (DNAq), cytological bands stained by Giemsa (cytoBand_gneg: non-stained, cytoBand_pos25 up to pos100: show increasing staining intensity), early, middle and late replication timing (RT), CCCTC-Binding factor binding sites that might correspond to DNA loops, insulators, chromatin anchoring point and borders between hetero- and euchromatin (CTCF-binding), opened chromatin structure (OPEN), DNase hypersensitive sites (DNaseHS), transcription factor binding sites (TF_binding_site).

Based on the revealed correlation between uracil distribution of non-treated cells and heterochromatin, as well as between uracil patterns of drug-treated cells and replication timing, AT content and the replication timing scores were also calculated on the genomic segments provided by the Segway analysis (Figure 4-figure supplement 3). Average replication timing scores were calculated using both replicates (Int90617792 and Int97243322) available in Replication Domain Database (Weddington et al., 2008). The applied command lines were the following:

```
$ awk '$4==0 {print $0}' segway.bed | sort -k1,1 -k2,2n | awk '{print $1 "\t" $2 "\t"
$3 "\t" NR}' > segway.0_ready.bed

$ awk '($2=$2-1) {print $1 "\t" $2 "\t" $3 "\t" $4}' Int90617792.bed >
Int90617792_0start.bed

$ awk '($2=$2-1) {print $1 "\t" $2 "\t" $3 "\t" $4}' Int97243322.bed >
Int97243322_0start.bed

$ awk '{print $1 "\t" $2 "\t" $3 "\t" NR}' segway.0.RTintersect.bed >
segway.0.RTintersect.ready.bed
```

```
$ bigWigAverageOverBed -bedOut=segway.0.RTscoreAverage.bed Int90617792.bw segway.0.RTintersect.ready.bed DEL.tab

$ awk '{($6=$3-$2)}1' segway.0.RTscoreAverage.bed > segway.0.RTscoreAverage.length.bed

$ awk '{($7=$5*$6)}1' segway.0.RTscoreAverage.length.bed > segway.0.RTscoreAverage.averBase.bed

$ awk '{(sum1 += $7) (sum2 += $6)} END {print (sum1/sum2)}' segway.0.RTscoreAverage.averBase.bed

$ bedtools nuc -fi refGenome_core_regions.fna -bed segway25.0_ready.bed > segway25.0.nuc.bed

$ awk '{(sum1+=$7) (sum2+=$10) (sum3+=$8) (sum4+=$9) (sum5+=$11) (sum6+=$12) (sum7+=$13)} END {print sum1 "\t" sum2"\t" sum3 "\t" sum4 "\t" sum5 "\t" sum6 "\t" sum7}' segway25.0.nuc.bed >> segway25.AT_cont_nuc.scoreSumma.csv
```

References

Karolchik, D., Hinrichs, A. S., Furey, T. S., Roskin, K. M., Sugnet, C. W., Haussler, D., & Kent, W. J. (2004). The UCSC Table Browser data retrieval tool. *Nucleic Acids Research*, *32*(90001), 493D–496. http://doi.org/10.1093/nar/gkh103

Layer, R. M., Pedersen, B. S., DiSera, T., Marth, G. T., Gertz, J., & Quinlan, A. R. (2018). GIGGLE: a search engine for large-scale integrated genome analysis. *Nature Methods*, *15*(2), 123–126. http://doi.org/10.1038/nmeth.4556

Quinlan, A. R., & Hall, I. M. (2010). BEDTools: a flexible suite of utilities for comparing genomic features. *Bioinformatics*, *26*(6), 841–842. http://doi.org/10.1093/bioinformatics/btq033

Uralsky, L. I., Shepelev, V. A., Alexandrov, A. A., Yurov, Y. B., Rogaev, E. I., & Alexandrov, I. A. (2019). Classification and monomer-by-monomer annotation dataset of suprachromosomal family 1 alpha satellite higher-order repeats in hg38 human genome assembly. *Data in Brief*, *24*, 103708. http://doi.org/10.1016/j.dib.2019.103708

Weddington, N., Stuy, A., Hiratani, I., Ryba, T., Yokochi, T., & Gilbert, D. M. (2008). ReplicationDomain: a visualization tool and comparative database for genome-wide replication timing data. *BMC Bioinformatics*, *9*, 530. http://doi.org/10.1186/1471-2105-9-530

Zerbino, D. R., Achuthan, P., Akanni, W., Amode, M. R., Barrell, D., Bhai, J., … Flicek, P. (2018). Ensembl 2018. *Nucleic Acids Research*, *46*(D1), D754–D761. http://doi.org/10.1093/nar/gkx1098
