# Supplementary material for: Genome-wide alterations of uracil distribution patterns in human DNA upon chemotherapeutic treatments
Source: eLife. 2020 Sep 21;9:e60498. doi: 10.7554/eLife.60498 (PMC7505663; doi:10.7554/eLife.60498)
Supplement: Supplementary file 5. [file elife-60498-supp5.pdf]

Supplementary file 5

Detailed comparison of uracil-DNA pattern to replication timing data

Detailed correlation analysis between uracil-DNA enrichment and replication timing was also done using the R (1) script below (cf. Figure 4D and Appendix 1-figure 2C). Replication timing data (bigWig files with 5000 bp binsize) were downloaded from ReplicationDomain database: Int90617792 for HCT116; Int57383924 for HEK293; Int37482971 for K562. The R script applied for the Figure 4D generation is given below.

Library structure was the following:

```
├── bigwig
│   ├── 5FdUR_UGI_HCT116_merged_IP_vs_son.bin100bp.smooth5000.RPGC.log2.bw
│   ├── 5FdUR_UGI_HCT116MMR_merged_IP_vs_son.bin100bp.smooth5000.RPGC.log2.bw
│   ├── NT_UGI_HCT116_merged_IP_vs_son.bin100bp.smooth5000.RPGC.log2.bw
│   ├── NT_UGI_HCT116MMR_merged_IP_vs_son.bin100bp.smooth5000.RPGC.log2.bw
│   ├── RTX_UGI_HCT116_merged_IP_vs_son.bin100bp.smooth5000.RPGC.log2.bw
│   ├── RTX_UGI_HCT116MMR_merged_IP_vs_son.bin100bp.smooth5000.RPGC.log2.bw
│   └── WT_HCT116_merged_IP_vs_son.bin100bp.smooth5000.RPGC.log2.bw
├── color codes.xlsx
├── Int90617792
├── plots
│   └── Uracil_overrepresentation_0521.pdf
└── Uracil_plot_1521.R
```

```
#used packages
library(tidyverse)
library(BSgenome.Hsapiens.NCBI.GRCh38)
#input the replication timing data
rt <- read.table(„bigwig/Int90617792”)
names(rt) <- c(„Chr”, „Start”, „End”, „RT”)
GRanges(rt) -> rtgr
rtgr <- keepSeqlevels(x = rtgr, value = seqlevels(rtgr)[1:24], pruning.mode = "coarse")
#list of bigwig files
list.files(path = " bigwig/", pattern = "*.bw") -> bwlist
gsub("_merged_IP_vs_son.bin100bp.smooth5000.RPGC.log2.bw", "", bwlist) -> namelist
colours <- c(rgb(1, 0, 0), rgb(1, 0.5, 0), rgb(1, 1, 0),
             rgb(0, 0.5, 0), rgb(0, 1, 0), rgb(0, 0, 1),
             rgb(0, 1, 1))
dir.create("plots/")
#list to store the aggregated data
ll <- vector(mode = "list", length = length(bwlist))
names(ll) <- namelist
#the for cycle will match each of the U-DNA-Seq data to RT and make pictures for all one by one
```

```
for (b in seq_along(bwlist)) {
  #input bigwig files
  bw1 <- import.bw(paste0("readfiltered_bwcomp/", bwlist[b]))
  GRanges(rt) -> rtgr
  #ensuring that only the common chromosomes and scaffolds will be considered
  bw1 <- import.bw(paste0("Adam_RTvsUracil//", bwlist[b]))
  bw1 <- keepSeqlevels(x = bw1, value = seqlevels(rtgr), pruning.mode = "coarse")
  #the RT data are given in bins of 5000 bases, while the uracil enrichment was calculated for bins of 100 bases
  #the principle of the comparison is first to define the overlaps between the intervals of the two datasets, then to
average the uracil enrichment scores for the bins of RT data. In this way, the object tt will contain both RT and the
corresponding average of uracil enrichment scores for each bin of 5000 bases.
  olaps <- findOverlaps(subject = rtgr, query = bw1)
  as.data.frame(olaps) %>% mutate(U_score = bw1$score[queryHits]) %>% group_by(subjectHits) %>%
summarize(U_score_mean = mean(U_score))  -> oo
  oo %>%
    mutate(RT = rtgr$RT[subjectHits]) -> tt
  #the resulting data frame is copied to the appropriate position of the summarizing list
  ll[[namelist[b]]] <- tt
  rm(bw1)
  gc()
  print(b)
}
  #labelling the RT values to have 10 equal-sized groups for the further plotting
  .bincode(tt$RT, breaks = quantile(tt$RT, probs = seq(0, 1, length.out = 21))) -> tt$RT_Cut
#labelling the 10 equal sized groups according to the RT data on each elements of the summarizing list.
ll2 <- lapply(ll, function(x) mutate(x, RT_Cut = .bincode(RT, breaks = quantile(RT, probs = seq(0, 1, length.out =
11)))))
do.call(rbind.data.frame, ll2) -> df
#additional information on formatting
df$Sample <- rep(names(ll2), sapply(ll2, nrow))
df <- df[-which(is.na(df$RT_Cut)),]
df <- mutate(df,
             Sample = gsub("MMR", "_MMR", Sample),
             Sample = gsub("_HCT116", "", Sample),
             Sample = factor(Sample,
                    levels = c("WT", "NT_UGI",
                               "NT_UGI_MMR", "5FdUR_UGI",
                               "5FdUR_UGI_MMR", "RTX_UGI",
                               "RTX_UGI_MMR")))
#plot and save
ggplot(df, aes(x = factor(11-RT_Cut, levels = 1:10), y = U_score_mean, fill = Sample)) + geom_boxplot(size = .3,
outlier.shape = NA) + theme_classic() + coord_cartesian(ylim = c(-1.5, 1.5), clip = "off") + theme(axis.text =
element_text(size = 12, face = "bold"), axis.title = element_text(size = 16), axis.line = element_line(size = .5)) +
ylab(bquote(log[2] * "(Uracil enrichment)")) + xlab("")  + scale_y_continuous(breaks = round(seq(-1.6, 1.6, by = .4),
digits = 1)) + theme(legend.text = element_text(size = 12), legend.title = element_blank()) + scale_fill_manual(values
= colours)
ggsave(filename = "plots/Uracil_overrepresentation.pdf", width = 8, height = 5)
```

References

R Core Team (2018) R: A language and environment for statistical computing. *R Found. Comput. Vienna, Austria. URL https//www.R-project.org/*.
